# Optimizing Longitudinal Tobacco Cessation Treatment in Lung Cancer Screening: A Sequential, Multiple Assignment, Randomized Trial

**DOI:** 10.1001/jamanetworkopen.2023.29903

**Published:** 2023-08-24

**Authors:** Steven S. Fu, Alexander J. Rothman, David M. Vock, Bruce R. Lindgren, Daniel Almirall, Abbie Begnaud, Anne C. Melzer, Kelsey L. Schertz, Mariah Branson, David Haynes, Patrick Hammett, Anne M. Joseph

**Affiliations:** 1 Veterans Affairs Health Services Research and Development Center for Care Delivery and Outcomes Research, Minneapolis VA Health Care System, Minneapolis, Minnesota; 2 Department of Medicine, University of Minnesota, Minneapolis; 3 Department of Psychology, University of Minnesota, Minneapolis; 4 Division of Biostatistics, University of Minnesota, Minneapolis; 5 Biostatistics Core, Masonic Cancer Center, University of Minnesota, Minneapolis; 6 Survey Research Center, Institute for Social Research, University of Michigan, Ann Arbor; 7 Institute for Health Informatics, University of Minnesota, Minneapolis

## Abstract

**Importance:**

Nearly half of the 14.8 million US adults eligible for lung cancer screening (LCS) smoke cigarettes. The optimal smoking cessation program components for the LCS setting are unclear.

**Objective:**

To assess the effect of adding a referral to prescription medication therapy management (MTM) to the tobacco longitudinal care (TLC) program among patients eligible for LCS who smoke and do not respond to early tobacco treatment and to assess the effect of decreasing the intensity of TLC among participants who do respond to early treatment.

**Design, Setting, and Participants:**

This randomized clinical trial included patients who currently smoked cigarettes daily and were eligible for LCS. Recruitment took place at primary care centers and LCS programs at 3 large health systems in the US and began in October 2016, and 18-month follow-up was completed April 2021.

**Interventions:**

(1) TLC comprising intensive telephone coaching and combination nicotine replacement therapy for 1 year with at least monthly contact; (2) TLC with MTM, MTM offered pharmacist-referral for prescription medications; and (3) Quarterly TLC, intensity of TLC was decreased to quarterly contact. Intervention assignments were based on early response to tobacco treatment (abstinence) that was assessed either 4 weeks or 8 weeks after treatment initiation.

**Main outcomes and Measures:**

Self-reported, 6-month prolonged abstinence at 18-month.

**Results:**

Of 636 participants, 228 (35.9%) were female, 564 (89.4%) were White individuals, and the median (IQR) age was 64.3 (59.6-68.8) years. Four weeks or 8 weeks after treatment initiation, 510 participants (80.2%) continued to smoke (ie, early treatment nonresponders) and 126 participants (19.8%) had quit (ie, early treatment responders). The 18 month follow-up survey response rate was 83.2% (529 of 636). Across TLC groups at 18 months follow-up, the overall 6-month prolonged abstinence rate was 24.4% (129 of 529). Among the 416 early treatment nonresponders, 6-month prolonged abstinence for TLC with MTM vs TLC was 17.8% vs 16.4% (adjusted odds ratio [aOR] 1.13; 95% CI, 0.67-1.89). In TLC with MTM, 98 of 254 participants (39%) completed at least 1 MTM visit. Among 113 early treatment responders, 6-month prolonged abstinence for Quarterly TLC vs TLC was 24 of 55 (43.6%) vs 34 of 58 (58.6%) (aOR, 0.54; 95% CI, 0.25-1.17).

**Conclusions and Relevance:**

In this randomized clinical trial, adding referral to MTM with TLC for participants who did not respond to early treatment did not improve smoking abstinence. Stepping down to Quarterly TLC among early treatment responders is not recommended. Integrating longitudinal tobacco cessation care with LCS is feasible and associated with clinically meaningful quit rates.

**Trial Registration:**

ClinicalTrials.gov Identifier: NCT02597491

## Key Points

**Question:**

Does adding a referral to pharmacy for prescription medication therapy management (MTM) to the evidence-based tobacco longitudinal care (TLC) program improve smoking abstinence rates compared with TLC without MTM among patients who are eligible for lung cancer screening (LCS), who smoke, and who do not respond to early tobacco treatment?

**Findings:**

In this randomized clinical trial of 636 patients who were eligible for LCS and smoked daily, adding a referral to MTM with TLC for participants who did not respond to initial TLC treatment did not improve long-term smoking abstinence (17.8% vs 16.4%).

**Meaning:**

These findings suggest that TLC was most effective when implemented without modification.

## Teaser

This randomized clinical trial assesses the effect of adding a referral to prescription medication therapy management to the tobacco longitudinal care program among patients eligible for lung cancer screening.

## Introduction

The landmark National Lung Screening Trial demonstrated that annual low-dose computerized tomography (LDCT) for lung cancer screening (LCS) reduces lung cancer death by 20%.^1,2,3^ Since 2013, annual LDCT has been recommended for adults aged 55 to 80 years with a 30 pack-year or more smoking history and who currently smoke or have quit within the past 15 years.^4,5,6,7^ To address lung cancer disparities by sex and race or ethnicity^8,9^ the eligible population was expanded in 2021 to adults aged 50 to 80 years and a 20 pack-year or more smoking history (14.8 million estimated).^10^ About 48% of patients eligible for LCS currently smoke cigarettes.^11^ Annual LCS creates a clinical obligation to develop and implement efficient and effective programs to address smoking in the LCS setting. Furthermore, patients who quit smoking and complete annual LDCT screening experience the greatest mortality reduction.^12^

Clinical trials have established the efficacy of behavioral treatment, medication treatment, and combination behavioral and medication treatment for long-term smoking cessation.^13^ However, the optimal smoking cessation program components for the LCS setting are unclear.^14,15^ One approach to improving long-term abstinence rates is the use of adaptive interventions. Adaptive interventions describe how to sequence treatment options (such as type, frequency, delivery mode) using information about individuals, including response to previous interventions. For example, for individuals showing early signs of difficulty with quitting, adaptive interventions might dictate which additional treatment to deliver and its timing. Adaptive interventions can also suggest treatment options for individuals who experience early success with quitting.^16^ Interventions that adapt to the changing needs and experiences of the individual are key to the management of chronic diseases like smoking.^17^

Incorporating chronic care model^18,19^ principles, the Tobacco Longitudinal Care (TLC) program is tobacco treatment comprised of intensive telephone coaching and combination nicotine replacement therapy (NRT).^17^ A unique feature of TLC is that the program encourages participants who relapse to set a new quit date and if they are unwilling to do so, to try to reduce their smoking in anticipation of setting a new quit date.

TLC delivered for 1 year was previously demonstrated to be efficacious in a confirmatory, randomized clinical trial.^17^ The current study reports the main results of the Program for Lung Cancer Screening and Tobacco Cessation (PLUTO) trial.^20^ PLUTO was designed to test different adaptive interventions based on TLC, which health systems could implement to address smoking in the LCS setting. The PLUTO trial participates in the Smoking Cessation at Lung Examination (SCALE) collaboration sponsored by the National Cancer Institute (NCI), a set of clinical trials evaluating smoking cessation interventions delivered in the LCS setting.^21^

PLUTO used a 2-stage, sequential, multiple assignment, randomized trial (SMART) design.^22,23,24^ SMART trials are not confirmatory randomized trials; rather, they are designed to provide evidence for how best to construct an adaptive intervention.^25^ The primary aim was to assess the effect of adding referral to prescription medication therapy management (MTM) to TLC among participants who did not respond early to smoking cessation treatment (hereafter referred to as early treatment nonresponders, defined as any smoking, even a puff, in the last 7 days).^26^ We hypothesized that among early treatment nonresponders, long-term abstinence rates (primary outcome) would be higher in participants randomized to TLC with MTM compared with TLC alone without MTM. Our secondary aim assessed the effect of decreasing the intensity of the TLC program from at least monthly contact to at least quarterly contact among participants who did respond to early treatment (hereafter referred to as early treatment responders, defined as no smoking, even a puff, in the last 7 days). We hypothesized that more intensive follow-up would improve long-term abstinence rates.

## Methods

### Study Design Overview

**Figure 1.**
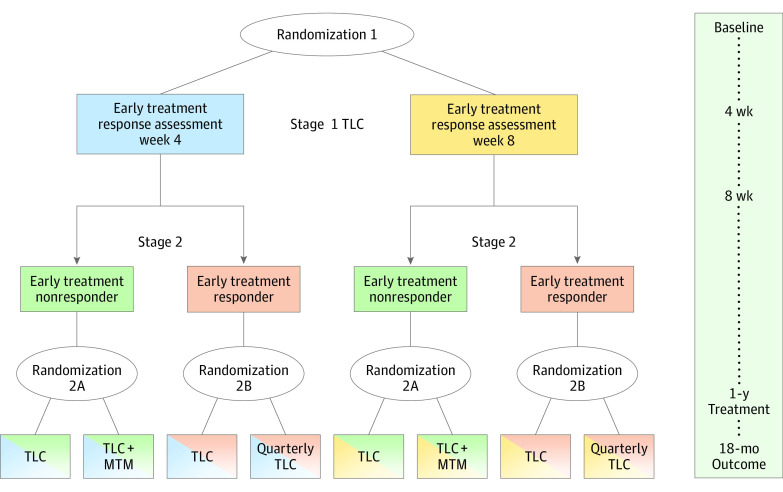
Sequential, Multiple Assignment, Randomized TrialDesign MTM indicates medication therapy management; TLC, tobacco longitudinal care; TLC + MTM, tobacco longitudinal care with medication therapy management.

The PLUTO trial was approved by the institutional review boards at the University of Minnesota, the Minneapolis Veterans Affairs Health Care System, and the Allina Health System. Written informed consent was obtained. The study protocol and methods have been previously described (Supplement 1).^20^ Participants received 1 of 3 TLC-based interventions based on defined decision rules (Figure 1) for 1 year. Each participant was randomized twice. In stage 1, all participants initiated TLC and were randomized to assess early response to TLC treatment at either 4 weeks vs 8 weeks (randomization 1, the results of which are not included in this article). In stage 2, participants were randomized to different interventions. Early treatment nonresponders were randomized to continue TLC with a minimum of monthly contact or TLC with MTM (randomization 2A). MTM provided access to prescription medications delivered by a pharmacist with prescribing privileges. Early treatment responders were randomized to continue TLC with a minimum of monthly contact or TLC with less intensive quarterly contact (Quarterly TLC; randomization 2B). PLUTO started recruitment October 2016 and completed the 18-month survey in April 2021 during the COVID-19 pandemic. Electronic medical record (EMR) data extraction, data cleaning, and analysis occurred from June 2021 to December 2022.

### Study Participants

Inclusion criteria were having LDCT for LCS scheduled or ordered or being LCS eligible, currently smoking daily, being 55 to 80 years old, and being willing to choose a quit date within the next 12 weeks. Exclusion criteria were having an unstable psychiatric disease or cognitive impairment, currently participating in smoking cessation treatment, not having a phone, or not speaking English.

### Interventions

#### TLC

TLC is a counseling-based intervention formulated on a chronic care model for tobacco treatment, including recycling treatment tools and relapse prevention. The TLC protocol^16,17^ includes contingencies for whether participants are abstinent, have relapsed but reduced smoking, have resumed the original amount of smoking, or have not quit at all.

Six scheduled counseling calls were provided during the first 4 weeks and a seventh call at week 8. After this initial sequence of calls, participants were contacted at least monthly by their coach, but the frequency of calls may have been higher around quit dates after relapse. The focus of counseling was cessation. Combination NRT was recommended^27^ and mailed free of charge by the coach.

#### TLC With MTM

In TLC with MTM, TLC was expanded to include referral and consultation with a pharmacist to prescribe bupropion, varenicline, nicotine spray, nicotine inhaler, or combination medications (eg, bupropion with NRT). Because this was a pragmatic trial, participants could decline referral. If referred, participants were not obliged to use medications, but pharmacists promoted their use. Prescribed medications were covered by participant’s health insurance. The first visit with the pharmacist was in-person, if possible, and about 1 hour in duration. Typically, 3 to 6 follow-up phone calls (5 to 10 minutes duration) were offered throughout a 12-week period but depended on participant interest and progress. The pharmacist was available until the end of the 1-year intervention period. While the TLC counselor continued to interact with the participant during MTM, the pharmacist had primary responsibility for medication management.

#### Quarterly TLC

Early treatment responders were assigned to receive continued TLC or Quarterly TLC and counselors called participants every 3 months for 1 year. They provided the same coaching options as TLC, but on a less intensive schedule. Participants could initiate additional calls.

### Research Data Sources and Data Collection

Data sources included telephone surveys, the EMR, and counselor records. PLUTO provided financial incentives for completing research surveys ($20 to $25) but not for treatment participation. Research assistants involved in data collection were blinded to intervention assignments.

### Outcome Measures

The primary outcome was self-reported, 6-month prolonged abstinence at 18 months^26^; smoking at least once on 7 consecutive days or at least once on 2 consecutive weekends in the 6-month period defined nonabstinence. Initial biochemical verification of smoking abstinence with salivary cotinine was disrupted by the COVID-19 pandemic in March 2020. In May 2020, we attempted to use CO (carbon monoxide) smokelyzers to verify smoking abstinence^28^; however, product availability was limited and the data safety and management board approved a protocol change to self-reported 6-month prolonged abstinence as the primary outcome. Secondary outcomes included 7-day point prevalence abstinence at 12 months and 18 months; any smoking in the past 7 days, even a puff, defined nonabstinence.

### Sample Size and Power Analysis

The sample size was chosen to ensure sufficient statistical power for the primary outcome, comparing TLC with TLC with MTM among early treatment nonresponders. Sample size formula for the number of early treatment nonresponders needed for the primary analysis was equivalent to standard calculations for 2-group randomized trials. We reestimated the sample size in April 2019 using blinded data to estimate the proportion of early treatment nonresponders and proportion achieving prolonged abstinence across both groups. Seven hundred participants provided approximately 90% power to detect a 10% difference in 6-month prolonged abstinence between TLC and TLC with MTM, assuming 80% of the sample would be early treatment nonresponders and 10% would achieve prolonged abstinence with TLC.

### Statistical Analysis

All analyses were intention-to-treat (ITT). All hypothesis tests were 2-sided with *P* < .05 considered statistically significant. Baseline characteristics were summarized overall and by the 2 comparison groups for the primary aim (defined by randomization 2A) and by the 2 comparisons groups for the secondary aim (defined by randomization 2B). To assess the primary aim (comparison of TLC with MTM vs TLC) we fit logistic regression models among early treatment nonresponders with covariates for age, baseline cigarettes per day, site, timing of assessment to early treatment (4-week vs 8-week assessment), in addition to an indicator for TLC with MTM vs TLC. A similar set of logistic regression models among early treatment responders were fit to assess secondary aim 1 (comparison of TLC to Quarterly TLC). The amount of treatment delivered was summarized by the frequencies or the median. Comparisons between randomized groups were conducted by χ^2^ or Wilcoxon rank-sum test. We addressed missing data using multiple imputation for the primary outcome and reported complete cases and additional sensitivity analyses for secondary outcomes (eTable in Supplement 2).

## Results

**Figure 2.**
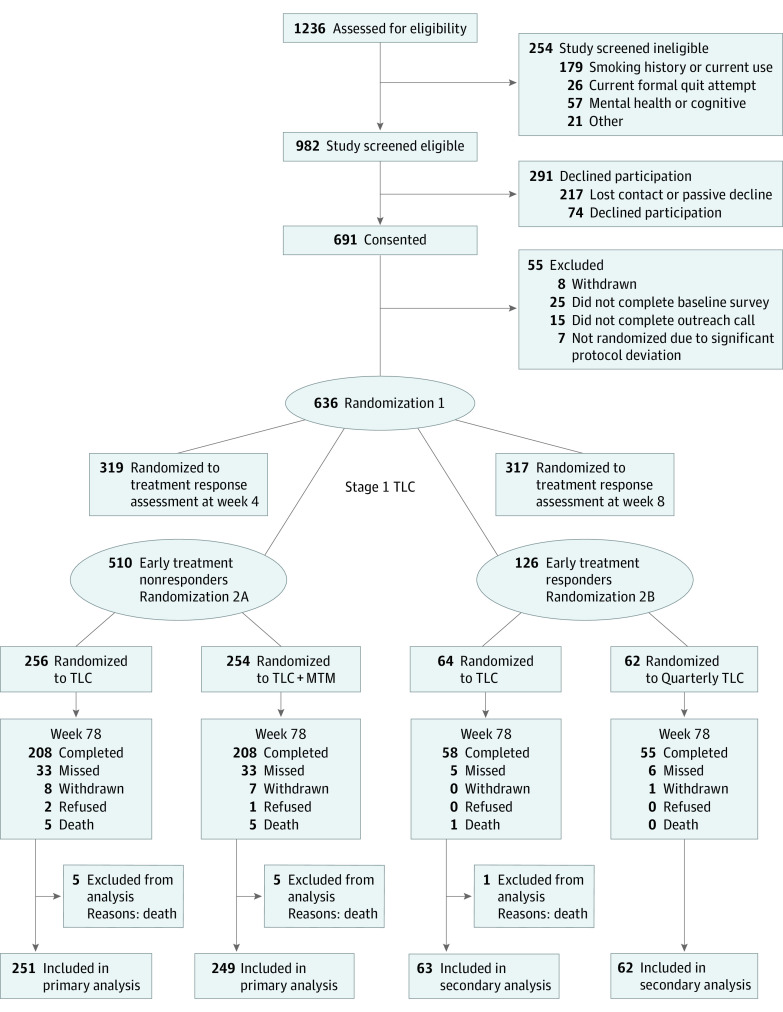
Study Flow Diagram MTM indicates medication therapy management; TLC, tobacco longitudinal care.

The ITT sample used for data analysis included 636 participants. The CONSORT^29^ diagram is shown in Figure 2. Of 1236 individuals screened, 982 were eligible, 691 consented, and 636 completed an outreach call and were randomized. After stage 1, 510 were early treatment nonresponders (80.2%) and 126 (19.8%) were early treatment responders. Data collection completion rates at 4, 8, 12, 26, 52, and 78 weeks (primary end point) for the 636 participants were 92.1% (586 participants), 90.6% (576 participants), 89.3% (568 participants), 85.8% (546 participants), 85.4% (543 participants), and 83.2% (529 participants), respectively.

### Baseline Characteristics

Baseline characteristics are presented in Table 1. The median (IQR) age of participants was 64.3 (59.6-68.8) years and 228 (35.9%) of the cohort was female. Median (IQR) cigarettes per day was 20.0 (10-20), and 485 participants (76.3%) smoked within 30 minutes of waking. Median (IQR) pack-years was 47.1 (36.3-59.8), and 271 (42.6%) had tried to quit in the past year. Most participants had previously tried NRT (542 [85.2%]) and prescription cessation medications (379 [59.6%]).

**Table 1.** Selected Baseline Characteristics by TLC Intervention Group (N = 636)^a^

Characteristics	Participants, No (%) (N = 636)			
	Randomization 2A: primary aim		Randomization 2B: secondary aim	
	TLC (n = 256)	TLC + MTM (n = 254)	TLC (n = 64)	Quarterly TLC (n = 62)
Age, median (IQR), y	63.1 (58.9-68.5)	64.4 (59.8-68.6)	66.0 (60.2-69.8)	66.1 (60.7-69.7)
Sex				
Female	92 (35.9)	97 (38.2)	21 (32.8)	39 (31.0)
Male	164 (64.1)	157 (61.8)	43 (67.2)	23 (69.0)
Race				
Black	12 (4.7)	16 (6.4)	2 (3.1)	4 (3.2)
White	226 (88.6)	220 (88.0)	62 (96.9)	118 (93.7)
Other^b^	17 (6.7)	14 (5.6)	0	4 (3.2)
Ethnicity				
Hispanic	3 (1.2)	3 (1.2)	0	0
Non-Hispanic	253 (98.8)	249 (98.8)	64 (100.0)	62 (100.0)
Education				
High school graduate or less	86 (33.7)	94 (37.0)	18 (28.1)	41 (32.5)
Vocational training or some college	110 (43.1)	110 (43.3)	30 (46.9)	55 (43.7)
Bachelor’s or graduate degree	59 (23.1)	50 (19.7)	16 (25.0)	30 (23.8)
Marital status, married or living with partner	115 (45.3)	107 (42.1)	31 (48.4)	67 (53.1)
Household income				
<$15 000	31 (13.0)	35 (15.2)	9 (15.3)	14 (12.3)
$15 000-$34 999	52 (21.8)	62 (27.0)	13 (22.0)	23 (20.2)
$35 000-$64 999	69 (28.9)	68 (29.6)	15 (25.4)	35 (30.7)
$65 000-$100 000	58 (24.3)	40 (17.4)	16 (27.1)	28 (24.6)
>$100 000	29 (12.1)	25 (10.9)	6 (10.2)	14 (12.3)
CPD, median (IQR)	19.5 (10.0-20.0)	20.0 (11.0-20.0)	15.0 (10.0-20.0)	15.0 (10.0-20.0)
Smoking within 30 min of waking	201 (85.2)	202 (95.5)	41 (64.1)	41 (66.1)
Pack-years, median (IQR)	45.2 (34.9-58.1)	48.5 (38.7-66.6)	46.5 (31.7-55.2)	46.9 (37.0-54.5)
Use e-cigarettes, every day or some days	20 (7.8)	14 (5.5)	5 (7.8)	2 (3.2)
Use marijuana, every day or some days	53 (20.8)	45 (17.7)	13 (20.3)	12 (19.4)
Tried quitting past 12 mos	93 (36.3)	111 (43.7)	34 (53.1)	33 (53.2)
Tobacco quit programs				
In-person 1-on-1 counseling	37 (14.5)	38 (15.0)	10 (15.6)	10 (16.1)
In-person group counseling	30 (11.7)	27 (10.6)	6 (9.4)	7 (11.7)
Telephone help or quit line	50 (19.6)	63 (24.8)	12 (18.8)	7 (11.3)
Internet program or phone app	10 (3.9)	8 (3.2)	0	1 (1.6)
Used any form of NRT in the past	216 (84.4)	218 (85.8)	53 (82.8)	55 (88.7)
Used prescription cessation medications in the past	150 (58.6)	154 (60.6)	34 (53.1)	41 (66.1)
Health status (SF-12), general health				
Excellent	9 (3.5)	3 (1.2)	1 (1.6)	5 (8.1)
Very good	61 (23.8)	53 (20.9)	11 (17.2)	16 (25.8)
Good	91 (35.6)	103 (40.6)	31 (48.4)	22 (35.5)
Fair	74 (28.9)	69 (27.2)	17 (26.6)	18 (29.0)
Poor	21 (8.2)	26 (10.2)	4 (6.3)	1 (1.6)
Charlson Comorbidity Index, median (IQR)	3.0 (2.0-5.0)	3.0 (2-5)	3.0 (2-5)	3.0 (2-4)
Self-reported LDCT lung cancer screening	179 (69.9)	184 (72.4)	49 (76.6)	53 (85.5)

Abbreviations: CPD, cigarettes per day; LDCT, low-dose computerized tomography; MTM, medication therapy management; NRT, nicotine replacement therapy; SF-12, 12-Item Short Form Health Survey; TLC, tobacco longitudinal care; TLC + MTM, tobacco longitudinal care with medication therapy management.

^a^ N = 622 to 636, depending on the baseline characteristic.

^b^ Other includes Asian individuals, Native Hawaiian or Pacific Islander individuals, American Indian or Alaska Native individuals.

### Abstinence Outcomes

The 18 month follow-up survey response rate was 83.2% (529 of 636 participants). Across TLC intervention groups, at week 78 (6 months after the end of the intervention), the overall 6-month prolonged smoking abstinence rate was 24.4% (129 of 529 participants) and the overall 7-day point prevalence smoking abstinence rate was 36.1% (191 of 529 participants). Abstinence rates by TLC intervention group are shown in Table 2. Primary outcome data were missing in 107 participants (16.8%). In the primary analysis using multiple imputation among early treatment nonresponders at week 78, 37 of 208 participants (17.8%) in TLC with MTM self-reported 6-month prolonged abstinence compared with 34 of 208 participants (16.4%) in TLC (adjusted odds ratio [aOR], 1.13; 95% CI, 0.67-1.89; *P* = .66). A complete case analysis (n = 416) showed similar results (aOR, 1.11; 95% CI, 0.66-1.86). A third analysis assuming that participants with missing data were continuing to smoke showed comparable results (aOR, 1.11; 95% CI, 0.67-1.85). Seven-day point prevalent abstinence for TLC with MTM was 62 of 208 (29.8%) compared with 57 of 208 (27.4%) in TLC (aOR, 1.14; 95% CI, 0.74-1.74).

**Table 2.** Observed and Adjusted Abstinence Rates and Sensitivity Analyses

Participant group	Participants, observed proportion (unadjusted), complete case analysis, No. (%) (n = 416)^a^		Adjusted odds ratio, TLC+MTM compared with TLC (95% CI)		
	TLC	TLC + MTM	Complete case analysis (n = 416)^a^	Assuming missing = smoking analysis (n = 500)^a^	Multiple imputation analysis (n = 500)^b^
**Early treatment nonresponders**					
Week 78: 6-mo prolonged abstinence	34/208 (16.4)	37/208 (17.8)	1.11 (0.66-1.86)	1.11 (0.67-1.85)	1.13 (0.67-1.89)^c^
Week 78: 7-d point-prevalent abstinence	57/208 (27.4)	62/208 (29.8)	1.14 (0.74-1.74)	1.09 (0.76-1.56)	NA
Week 52 (end of treatment): 7-d point-prevalent abstinence	57/216 (26.4)	58/216 (26.9)	1.03 (0.67-1.60)	0.97 (0.67-1.41)	NA

Abbreviations: TLC, tobacco longitudinal care; TLC + MTM, tobacco longitudinal care + medication therapy management.

^a^ At Week 78 data were collected from 416 participants, at Week 52 data were collected from 432 participants.

^b^ Primary outcome with deaths excluded. An additional sensitivity analysis including deaths showed similar results.

^c^ *P* = .66.

Among early treatment responders at week 78, 24 of 55 participants (43.6%) in Quarterly TLC reported 6-month prolonged abstinence compared with 34 of 58 (58.6%) in TLC (aOR, 0.54; 95% CI, 0.25-1.17). For Quarterly TLC, 32 of 55 participants (58.2%) had a 7-day point prevalent abstinence compared with 40 of 58 participants (69.0%) for TLC (aOR, 0.64; 95% CI, 0.29-1.40).

Only 79 of 141 (56%) saliva or CO kits that were sent out during the trial were returned; thus there is limited data regarding the biochemical verification of self-reported abstinence. Of the 59 participants who reported abstinence, 45 (76.3%) were biochemically confirmed, and there were not significant differences between comparison groups.

### Treatment Delivered

Table 3 summarizes coaching and medication treatment delivered to participants. Most participants received combination NRT (range, 82.3%-89.1% across TLC groups); there were no significant differences between groups. Additionally, 116 of 254 participants (45.7%) assigned to MTM accepted referral to MTM, and 98 of 254 (38.6%) completed at least 1 visit. The median (IQR) number of pharmacy visits per participant for those who completed at least 1 MTM visit was 6.5 (3.0 to 10.0) visits. Significantly more early treatment nonresponders received any prescription medications in TLC with MTM compared with TLC (86/256 [33.9%] vs 48/256 [18.8%]; *P* < .001), but among early treatment responders the difference in receipt of prescription medications between TLC and Quarterly TLC was not significant (4/64 [6.3%] vs 9/62 [14.5%]; *P* = .15). There was no significant difference in the median (IQR) number of coaching calls between TLC and TLC with MTM (12.0 [9.5-15.0] calls vs 12.0 [7.5-15.0] calls), or between TLC and Quarterly TLC (12.0 [8.0-15.0] calls vs 12.0 [9.0-14.0] calls).

**Table 3.** Coaching and Medication Treatment Delivered Over 12 Months

Treatment	Participants, No (%)					
	Early treatment nonresponders: randomization 2A			Early treatment responders: randomization 2B		
	TLC (n = 256)	TLC + MTM (n = 254)	*P* value	TLC (n = 64)	Quarterly TLC (n = 62)	*P* value
Coaching						
No. coaching calls, median (IQR)	12.0 (7.5-15)	12.0 (8-15)	.15	12.0 (9.5-15)	12.0 (9-14)	.72
Total duration of calls, median (IQR), min	130.5 (86.5-170.5)	133.0 (92-182)	.19	127.5 (98-170)	131.5 (98-197)	.45
Medications						
Single NRT only over 12 mos of treatment (patch, lozenge, or gum)	36 (14.1)	30 (11.8)	.51	5 (7.8)	6 (9.7)	.76
No. of weeks NRT provided, median (IQR)	4.0 (2-8)	6.0 (2-16)		6.0 (2-10)	7.0 (2-38)	
More than 1 NRT (patch, lozenge, gum) over 12 mos of treatment	211 (82.4)	215 (84.7)		57 (89.1)	51 (82.3)	
Patch, median (IQR) weeks NRT provided	12.0 (4-20)	10.0 (4-20)	.55	10.0 (6-18)	12.0 (4-18)	.32
Lozenge, median (IQR) weeks NRT provided	4.0 (2-10)	4.0 (2-8)		4.0 (2-12)	5.0 (2-12)	
Gum, median (IQR) weeks NRT provided	4.0 (2-8)	4.0 (2-8)		6.0 (2-22)	6.0 (2-10)	
Nicotine spray or inhaler^a,b^	17 (6.6)	34 (13.4)	.01	2 (3.1)	1 (1.6)	1.0
Bupropion^a,b^	17 (6.6)	32 (12.6)	.02	1 (1.6)	1 (1.6)	1.0
Varenicline^a,b^	19 (7.4)	39 (15.4)	.01	1 (1.6)	7 (11.3)	.03
Any of the 4 prescription medications^b^	48 (18.8)	86 (33.9)	<.001	4 (6.3)	9 (14.5)	.15

Abbreviations: NRT, nicotine replacement therapy; TLC, tobacco longitudinal care; TLC + MTM, tobacco longitudinal care with medication therapy management.

^a^ Current usage was assessed during the 6 data collection calls.

^b^ Ever during 12 months of treatment.

## Discussion

The purpose of this SMART was to generate data to select components for an evidence-based adaptive intervention for smoking cessation treatment in the LCS setting. Among participants who did not respond to the initial TLC treatment, there were no significant differences in long-term or short-term abstinence between TLC with or without the availability of prescription medications. Among participants who did respond to the initial TLC treatment, results suggested that TLC was more effective with at least monthly contact than quarterly contact, but the difference was not statistically significant. Clinically meaningful long-term quit rates were observed across TLC intervention groups. Based on the findings from these primary analyses, TLC was most effective when implemented without modification in the LCS setting.

Findings from PLUTO inform how to design effective smoking cessation programs in the LCS setting. Based on the primary analyses, there is no evidence to suggest that setting up a referral program for participants to see a pharmacist for MTM (and possible prescription medications for smoking cessation) would yield much benefit. This may have implications for resource allocation because combination NRT is available over the counter and can be dispensed by trained staff, which is less resource intensive than providing prescription medications that require licensed clinicians. There are several potential explanations for these findings. Among early treatment nonresponders, only 39% of participants completed at least 1 MTM visit. Baseline characteristics show that most participants had previously tried prescription medications; perhaps lack of prior success with medications influenced this decision. While prescription medication use was higher in TLC with MTM than in TLC, a number of participants (18.8% of those not randomized to MTM) accessed these medications outside the study (eg, through their health care clinician). Additionally, most participants used considerable amounts of combination NRT, and availability of prescription medications might not have provided important benefit in this context. This could be because coaches promoted combination NRT, it was free of cost, and/or combination NRT is equally effective to prescription medications. However, this finding may not be generalized to populations with limited access to care and prescription medications. Future research should assess whether there are subgroups who would benefit from referral to MTM to access prescription medications.

The comparison of TLC and Quarterly TLC for early responders suggest monthly follow-up confers long-term cessation advantage. The estimated difference of 14% in abstinence rates was not statistically significant, however, it is clinically significant, and this secondary analysis was underpowered. Based on this result, we recommend that clinicians do not step down to quarterly contact among early responders to TLC. It is likely that, even among early treatment responders, attention to relapse prevention, management of smoking lapses, and adjustment of NRT is important.

The results of PLUTO are important because they support feasibility and utility of using a chronic care model of tobacco treatment that incorporates medications and intensive counseling. Meaningful long-term quit rates (24.4%, 6-month prolonged abstinence) and short-term quit rates (36.1%, 7-day point prevalence abstinence) were observed overall across TLC groups and these rates compare favorably with other smoking cessation trials in the LCS setting. For example, Taylor et al^30^ tested a short-term (3-month duration) intensive telephone care intervention and found significant effects on abstinence at 3 months that disappeared at longer term follow-up with self-reported 7-day abstinence rates of 9% to 10% at 6 months. In 2 other SCALE trials, Foley et al^31^ reported 7-day abstinence rates of 13% at 6 months, and Tremblay et al^32^ reported 30-day abstinence rates of 13% to 14%. Clinical observational studies have reported quit rates in the LCS setting of 9% to 14%.^33,34,35^ Modeling studies support that integrating tobacco treatment with LCS yields cost-benefits of similar magnitude of LCS itself; for example, adding a tobacco cessation intervention with an effectiveness of 15% results in equivalent life-years gained as increasing LCS uptake from 30% to 100%.^36^ The quit rates observed in PLUTO suggest that the investment of resources to adopt a chronic care model in the LCS setting would be effective and cost-effective. Future research is needed to identify effective implementation strategies to promote adoption and integration of tobacco treatment into the LCS setting.^31^ PLUTO findings are also consistent with a recent systematic review that concluded that smoking cessation interventions delivering combination counseling and medications can be successfully implemented in the LCS setting and observed that more intensive interventions are likely to be more effective than less intensive interventions.^37^

### Limitations

This study has limitations. The primary analysis was adequately powered, but the sample size is relatively small for some secondary outcomes. The smoking abstinence data are self-reported; however, the biochemical verification data available do not suggest a difference in misreport by randomized comparison groups. Because the participant cohort was in the Midwest and predominantly White individuals, findings may have limited generalizability to racially and ethnically minoritized populations.

## Conclusions

In this SMART clinical trial, offering a referral program to MTM along with TLC for patients continuing to smoke did not offer additional benefit. This trial supports the feasibility and use of integrating longitudinal tobacco cessation care into the LCS setting. Long-term smoking abstinence rates for longitudinal care are higher than abstinence rates observed in shorter interventions and indicate the value of taking a longitudinal or chronic care approach to tobacco cessation. Findings also show that patients undergoing LCS who smoke accept TLC, as evidenced by their completion of a relatively large number of calls over a year-long period. Health systems should consider integrating longitudinal tobacco cessation care into the LCS setting.

## Notes

Supplement 1.
Trial Protocol and Statistical Analysis Plan

Supplement 2. **eTable.** Observed and Adjusted Abstinence Rates and Sensitivity Analyses for Early Treatment Responders

Supplement 3.
Data Sharing Statement
