# Supplementary material for: Optimizing Longitudinal Tobacco Cessation Treatment in Lung Cancer Screening: A Sequential, Multiple Assignment, Randomized Trial
Source: JAMA Netw Open. 2023 Aug 24;6(8):e2329903. doi: 10.1001/jamanetworkopen.2023.29903 (PMC10450571; doi:10.1001/jamanetworkopen.2023.29903)
Supplement: Supplement 1. — Trial Protocol and Statistical Analysis Plan [file jamanetwopen-e2329903-s001.pdf]

Supplemental Online Content

Fu SS, Rothman AJ, Vock DM, et al. Optimizing Longitudinal Tobacco Cessation Treatment in Lung Cancer Screening. *JAMA Netw Open.* 2023;6(8):e2329903. doi:10.1001/jamanetworkopen.2023.29903

eAppendix 1. Statistical Analysis Plan

eAppendix 2. Trial Protocol

This supplemental material has been provided by the authors to give readers additional information about their work.

© 2023 Fu SS et al. *JAMA Network Open.*

eAppendix 1. Statistical Analysis Plan

1.1 Objective of the Updated Statistical Analysis Plan

The objective of this updated statistical analysis plan (SAP) is to provide a description of the updated analytic strategy and the statistical methods that will be used to analyze the data for the Program for LUng cancer screening and TObacco cessation (PLUTO) Trial which uses a sequential multiple assignment randomized trial (SMART) design. The primary objective of the trial is to determine among incomplete responders to first stage tobacco longitudinal care (TLC) if medication therapy management (MTM) added to TLC is an effective approach for long-term tobacco cessation compared to TLC alone. The primary endpoint of this trial is a binary outcome of long-term tobacco cessation assessed 18 months after initial tobacco cessation treatment.

The primary statistical analysis plan was described in detail in the protocol paper (Fu et al., *Contemporary Clinical Trials*[1]). However, changes to the protocol and study status have necessitated updating the statistical analysis plan.

This updated SAP was prepared prior to unblinding of the data by statisticians and other members of the core PLUTO team who have been blinded to interim treatment comparisons for the duration of the trial.

Below we briefly summarize the status of the trial and some key blinded data that informed the preparation of this updated SAP.

1.2 Trial Status and Information That Informed the Updated SAP

1.2.1 Trial Status

The protocol underwent a number of minor changes throughout the study. Two key changes to the protocol are described below.

When the trial opened, participants were required to be scheduled for or have an order for first-time low dose CT for lung cancer screening. Due to fewer people undergoing lung cancer screening (both within the health systems studied in this trial and nationwide), we expanded the inclusion criteria. Eligibility was first expanded in January 2017 at the University of Minnesota and Minneapolis VA to include patients receiving repeat low dose CT for lung cancer screening, in addition to patients receiving first time scans. To further increase enrollment, we added a new site, Allina Health, in June 2018. In September 2018, criteria at the University of Minnesota (UMN) site were expanded to include all people who smoke who were eligible for lung cancer screening on the basis of their age and smoking history. Similarly, the criteria were expanded at the Minneapolis VA to include those who were eligible for lung cancer screening and a part of the lung cancer screening program regardless of their intention to receive another lung cancer screening.

Due to the COVID-19 pandemic-related safety protocols, saliva was no longer collected from participants after March 23, 2020. Thus, cotinine verification of smoking cessation was not able to be assessed after this date. From March 23, 2020 until May 19, 2020,

no participants were sent any biochemical verification. Beginning on May 19, 2020, participants were sent an iCO smokerlyzer to verify abstinence. Additionally, we attempted to send the iCO smokerlyzer to participants to whom we could not send a saliva kit from March 23, 2020 until May 19, 2020 even if those the data collection for those participants was out of window. We paused sending iCO smokerlyzers from July 2020 to December 2020 due to limited product availability.

1.2.2 Enrolment Summary

The first participant was enrolled on November 11, 2016; the last participant was enrolled on September 25, 2019. A total of 691 participants were enrolled across the three sites. Thirty-one participants withdrew prior to the baseline visits, and an additional 17 participants did not complete the outreach call after the baseline visit; thus, 643 participants were randomized to 4- or 8-week assessment of first stage TLC. A total of 510 participants were incomplete responders and were randomized to TLC vs. TLC + MTM; 126 participants were complete responders and randomized to TLC-Q vs. TLC-M. Seven participants were not randomized a second time. Thus, a total of 636 participants were randomized twice.

1.2.3 Summary of Pooled (Both Treatment Groups Combined) Follow-up Results

The final data collection was completed on May 1, 2021. Table 1 describes the data available from the Week 52 and Week 78 data collection calls. In general, data on abstinence is available if the participant only completed a mail-in survey (other secondary measures are not available). Among participants still alive, data are available on 86.8% (547/630) and 84.6% (529/625) of participants at Week 52 and Week 78.

Tables 2 and 3 summarize the number of participants randomized to each condition and the pooled (blinded), self-reported long-term abstinence.

Table 1: Data availability at Week 52 and 78 data collection calls.

Status at data collection call	Week 52 n=	Week 78 n=
Complete	517	494
Missed	63	77
Incomplete	0	0
Participant withdrew from study	15	16
Participant refused call	5	3
Participant only did mail-in survey	30	35
Death on study	6	11
Total	636	636

Table 2: Number of participants randomized to each condition

Complete responder n=126				Incomplete responder n=510			
Week 4		Week 8		Week 4		Week 8	
TLC_M	TLC-Q	TLC_M	TLC-Q	TLC-M	MTM	TLC-M	MTM
n=32	n=34	n=32	n=28	n=133	n=120	n=123	n=134

Table 3: Pooled smoking outcomes at Week 52 and 78.

Smoking outcomes	Week 52 n=	Week 78 n=
1) Smoked in past 7 days;		
No	187 (34.3%)	191 (36.1%)
Yes	359 (65.8%)	338 (63.9%)
Missing	90	107
2) Smoked in the last 30 days:		
No	166 (77.9%)	169 (78.2%)
Yes	47 (22.1%)	47 (21.8%)
Missing*	423	420
3) Smoked 7 consecutive days in last 6 months;		
No	123 (64.7%)	131 (67.9%)
Yes	67 (35.3%)	62 (32.1%)
Missing*	446	443
4) Smoked in 2 consecutive weeks in last 6		
months:	115 (79.3%)	130 (83.3%)
No	30 (20.7%)	26 (16.7%)
Yes	491	480
Missing*		
Prolong abstinence (#1, #3, and #4 =No)	113/636 (17.8%)	129/636 (20.3%)

1.2.4 Sample Size Re-Estimation

In April 2019, the blinded statisticians were provided the pooled (both treatment groups combined) proportions of complete and incomplete responders and the proportion of those achieve long-term abstinence among incomplete responders in order to re-estimate sample size. Based on the observed pooled proportions, we estimated between 75-80% of the sample would be incomplete responders and of those incomplete responders between 10-15% would achieve long-term abstinence with TLC alone. The protocol paper outlines that we designed the study to have at least 80% power to detect a risk difference of 10 percentage points. Table 4 outlines the power for the primary aim under these assumptions. Based on these data, we changed the planned enrollment from 1,000 to 700.

Table 4: Power estimates based on updated observed pooled proportions of the proportions of incomplete responders and proportion of incomplete responders 91 *achieving long-term abstinence.*

n=700					
**Proportion incomplete responders=80%**			**Proportion incomplete responders=75%**		
	**TLC achieving long-term abstinence**			**TLC achieving long-term abstinence**	
**TLC+MTM long-term abstinence**	**10%**	**15%**	**TLC+MTM long-term abstinence**	**10%**	**15%**
**7.5% diff**	73%	62%	**7.5% diff**	70%	60%
**10% diff**	91%	84%	**10% diff**	90%	82%

1.3 Summary of Changes to SAP

Based on the above information and additional data expected we made the following changes and clarifications to the SAP:

1. *Change in primary outcome:* Because saliva samples could not be collected for a portion of the sample due to COVID-19, biochemical verification could not be completed for all those self-reporting long-term abstinence. The primary outcome will be self-reported long-term abstinence at 78 weeks (18 months). This is defined as not smoking at all (even a puff) for the past 7 days and not smoking for 7 consecutive days during the past 6 months. As a supportive analysis, we will impute the missing biochemical verification data on those self-reporting long-term abstinence who declined biochemical verification, did not return the saliva/iCO smokerlyzer kits, or who were not sent a kit in window.

2. Adjusted analysis for primary and secondary aim: The enrolment for the trial was lower than anticipated. In particular, we initially anticipated between 650 and 750 incomplete responders whose outcomes would inform the analysis for the primary outcome. Based on the sample size re-estimation we anticipated between 525 and 560 incomplete responders. A total of 510 incomplete responders were randomized to second stage treatment. Similarly, initial power calculations assumed that there would be between 250 and 350 complete responders but only 126 participants were complete responders in this trial. In general, our other assumptions for the power calculations (e.g., long-term abstinence rate in the control group) were reasonable. To account for the under-enrolment, the primary analysis for the primary aim (comparison of TLC+MTM vs. TLC for incomplete responders) and secondary aim 1 (comparison of TLC-Q vs TLC-M) will be a logistic regression model with covariates for age, cigarettes per day, site (Minneapolis VA vs. others), first-stage randomization (4 vs. 8 week assessment) and second-stage randomized treatment group. In general, adjusted models tend to have higher power (although not guaranteed for a nonlinear model), but the interpretation of the

treatment effect estimate is challenging if there is treatment effect heterogeneity which is not accounted for in the model.[2] Similar adjusted models will also be the primary analysis for secondary aims 2 and 3. The unadjusted analyses described in the protocol paper will be a secondary analysis.

3. Methods to handle missing data: The SAP in the protocol paper specifies that "We will address missing response data using multiple imputation and additional sensitivity analyses." Multiple imputation will be done using the full conditional specification with predictive mean matching.[3 4 5] Covariates included in the imputation include age, gender, cigarettes per day at baseline, time to first cigarette at baseline, site, randomized treatment groups, and seven-day point prevalence at 4, 8, and 12 weeks, 6 months, and 12 months, in addition to 18 month long-term cessation. Note that some of these covariates may be missing as well and the full conditional specification enables one to impute these covariates as well. The imputation models for binary and continuous covariates will use logistic and linear regression models with main effect terms for all the other covariates. Twenty complete datasets will be imputed and the estimates from each the analysis of each complete datasets will combined using Rubin's combining rules. Participants who died will be recorded as a "failure," and we will not stochastically impute their endpoint. As a sensitivity analysis, we will also stochastically impute a cessation endpoint for those who died during the study. Note that only the primary endpoint will be imputed, imputation for other secondary endpoints will not be considered. Sensitivity analysis includes assuming those without data are still smoking and complete case analysis. Additional sensitivity analyses which may be considered (which do not assume that the missingness is ignorable) include joint models for longitudinal smoking cessation and nonresponse, pattern mixture models, and selection models.

4. Lung Cancer Screening Results: Secondary aim 4 addresses whether or not lung cancer screening results moderate smoking cessation outcomes. Due to changes in the eligibility criteria, participants may or may not have undergone lung cancer screening at any time during the 1 year of treatment provided by the study. For the purposes of this analysis, we will use the Lung RADS score from the first *screening* CT during the treatment period or just prior to the outreach call (the electronic medical record is queried up to 6 months prior to enrolment). When the Lung RADS score is considered an ordinal measure, those without CT scans will be considered to have a Lung RADS score of 0. As a sensitivity analysis, we will also group those participants with Lung RADS score of 1.

---

[1] Fu SS, Rothman AJ, Vock DM, et al. Program for lung cancer screening and tobacco cessation: Study protocol of a sequential, multiple assignment, randomized trial. *Contemp Clin Trials*. 2017;60:86-95. doi:10.1016/j.cct.2017.07.002

[2] FDA (2019). Adjusting for Covariates in Randomized Clinical Trials for Drugs and Biologics with Continuous Outcomes. *Draft Guidance for Industry*.

[3] Liu Y, De A. Multiple Imputation by Fully Conditional Specification for Dealing with Missing Data in a Large Epidemiologic Study. *Int J Stat Med Res*. 2015;4(3):287-295. doi:10.6000/1929-6029.2015.04.03.7

[4] Van Buuren, S. (2007). “Multiple Imputation of Discrete and Continuous Data by Fully Conditional Specification.” *Statistical Methods in Medical Research* 16:219–242.

[5] Van Buuren, S. (2012). Flexible Imputation of Missing Data. Boca Raton, FL: Chapman & Hall/CRC.

PROTOCOL COVER PAGE

eAppendix 2. Trial Protocol

**Protocol Title**	**Adaptive Interventions for Smoking Cessation in Lung Cancer Screening Programs**<br>*Alias:* Program for Lung Cancer Screening & Tobacco Cessation "PLUTO"
**Principal Investigator/Faculty Advisor**	Name: Anne Joseph
	Department: Medicine/General Internal Medicine
	Telephone Number: 612 625 3789
	Email Address: amjoseph@umn.edu
**Scientific Assessment**	Cancer Protocol Review Committee (CPRC)
**IND/IDE # (if applicable)**	N/A
**IND/IDE Holder**	N/A
**Investigational Drug Services # (if applicable)**	N/A
**Version Number/Date:**	Version 19 | Version Date 7/15/2020

REVISION HISTORY

Revision #	Version Date	Summary of Changes	Consent Change?
Original	03/25/2015	original to CPRC	n/a
1	08/17/2015	Revise section 5 to delete telephone follow up to invitation letter<br>Add that gift cards are Target	
2	06/20/2016	**Revisions to Section 5:**<br>(1) A mnemonic – "PLUTO" or "Program on Lung Cancer Screening and Tobacco Use" was developed for recruitment purposes and participant use<br>(2) Recruitment plan revised (patients may be referred to study by providers)<br>(3) Inclusion criteria amended to specify eligible candidates are to be recipients of first-time CT screening for lung cancer; clarified definition of daily smoker<br>(4) Exclusion criteria amended to clarify definitions of unstable psychiatric disease; added exclusion for recent cognitive impairment<br>**Revisions to Section 6:**<br>(1) Randomization (R1) will occur following baseline, at the time of the counseling outreach call<br>**Revisions to Section 7:**<br>(1) Follow-up data collection calls will be anchored to counseling outreach call, not the first quit date as previously stated<br>**Revisions to Appendices:**<br>(1) Included Appendix F: Data collection forms	Yes
3	08/03/2016	Revisions to Section 5:<br>(1)-In the event there is insufficient time to mail recruitment letter, patients may be approached at time of Shared Decision Making Visit or CT	No
4	08/25/2016	Revisions to Section 5:<br>(1) Eligibility criteria was amended to extend eligibility to individuals who have made a recent quit attempt given that during the last 30 days, when they were smoking regularly, they smoked on at least 5 out of 7 days of the week.<br>Revisions to Section 9:<br>(1) REDCap contingency plan added<br>Revisions to Appendix A:<br>(2) Counseling call content and objectives restructured<br>(3) Motivational interviewing, customer service added<br>(4) Missed call protocol amended<br>Revisions to Appendix F<br>(1) Updated eligibility questions<br>(2) Minor revisions throughout<br>(3) Changes to RPQ<br>Revisions to Appendix E<br>(1) Revised for consistency with changes to Appendix A, counseling objectives	No
5	11/8/2016	Revisions to Section 5:<br>Exclusion criteria amended to include Callahan screener for patients reporting recent cognitive impairment; smokers using NRT are eligible as long as they are not using it as part of a formal quit program	No
6	1/11/2017	Protocol revised for internal consistency<br>Revisions to Section 5:<br>(1) Recruitment window added (before LDCT or within 30 days following LDCT)<br>Revisions to Section 6:<br>(1) Randomization windows/timeline added<br>Revisions to Section 7.4:	No

		(1) 1st MTM encounter may occur via telephone if participant can't travel to site<br>(2) Parking validation will be provided for participants who park at CSC for MTM visit<br>Revisions to Appendix E:<br>(1) Tobacco treatment form updated<br>Revisions to Appendix F:<br>(1) Data collection forms revised throughout with operational changes<br>(2) Charlson index revised; addition of family hx of cancer; addition of employment status question<br>(3) Revisions to COPD screener	
7	2/1/2017	Revisions to Section 5:<br>Eligibility criteria changed – remove requirement for 1st time LDCT	No
8	3/29/2017	Revisions to Section 5:<br>1) Recruitment will also be conducted at FV community clinics<br>2) Patients with a current diagnosis of lung cancer are excluded<br>Revisions to Section 11.3<br>Telephone informed consent	Yes
9	4/24/2017	1. Section 5 was amended to remove a statement that recruitment at the Minneapolis VAMC will be capped to males every 6 months.<br>2. Section 5.2 was amended to clarify exclusion criteria regarding bupropion/varenicline use at time of eligibility screen.<br>Smokers will be eligible for participation if they have been using bupropion or varenicline for smoking cessation for over 8 weeks at the time of eligibility screening.<br>3. Appendix A Subappendix XI Crisis Management Protocol was revised and re-appended to the protocol as Appendix I. Crisis Management Protocol. It was also amended to include the Columbia-Suicide Severity Rating Scale (C-SSRS) and staff response triage points.<br>4. Appendix F. Data Collection Forms was amended to include a set of questions regarding previous lung cancer screening CTs. It was also amended to include the new eligibility question regarding current bupropion/varenicline use.	Yes
10	11/29/2017	A statement was added to Section 5 detailing addition of a follow-up recruitment letter for patients not currently participating in lung cancer screening (this letter is uploaded to Recruitment Letters). Eligibility criteria was amended in Section 5.2 to clarify that participation in a formal program, to include working with a pharmacist, is exclusionary. Patients with a current diagnosis of lung cancer will require study MD review and approval for inclusion. Section 7.4 is amended to add study procedures following randomization to Medication Therapy Management (MTM). Participants randomized to MTM will be notified by their TLC coach by mail in addition to telephone of allocation to new study treatment options (this letter is uploaded to Supporting Documents). MTM pharmacists will be provided with contact information of patients randomized to MTM to establish contact. Section 7.5 is amended to add a brief written survey to be mailed to participants who miss their 12 or 18 month data collection call (these letters and survey are uploaded to Supporting Documents). Section 7.7 is amended to describe study procedures following 12 months of study treatment; participants will be notified by mail in addition to telephone of completion of 12 months of treatment. For patients seeking additional smoking cessation resources, a brochure will be provided (both the termination of treatment and brochure are uploaded to Supporting Documents). Case report forms are amended in Appendix F. to reflect minor eligibility question changes and to add the Week 26, 52, and 78 follow-up smoking assessments. A new study logo to appear on all patient-facing documents is added in Appendix J.	No

		Additionally, the treatment guidelines were updates to include varenicline.	8
11	4/19/2018	Saliva protocol amended; Allina Health added as study site	
12	6/12/2018	Protocol, consent, and HIPAA are revised/reformatted for appropriate HRP templates	Yes
13	7/31/2018	Expanded eligibility criteria to include LCS eligible but scan incomplete patients	Yes
14	2/7/2019	Revisions to screening and PHQ-9 suicide items	No
15	6/26/2019	Added MyChart recruitment mechanism; revised risks section to indicate that smoking-related deaths are expected in this older cohort	No
16	9/16/2019	Section 22 revised to include information regarding SCALE collaboration	No
17	1/23/2020	Section 5.2.6 revised to specify how lung cancer screening and medication therapy management outcomes data will be obtained	No
18	3/27/2020	Section 13.0 Risks to Participants revised to include Section 13.4 COVID-19 Risk Mitigation Plan<br>Section 5.0 (subsections 5.2.5 and 5.2.7b Procedures Involved revised to include iCO™ Smokerlyzer® as an alternative method for biochemical verification of tobacco abstinence<br>Section 5.0 (subsection 5.2.6) Revised to include COVID-19 questions added to CRFs	No
19	7/15/2020	Section 5.2.6 revised to include an additional financial incentive for the final data collection call	No

Table of Contents

1.0 Objectives 7
2.0 Background 7
3.0 Study Endpoints/Events/Outcomes 11
4.0 Study Intervention(s)/Investigational Agent(s) 11
5.0 Procedures Involved 12
6.0 Data and Specimen Banking 22
7.0 Sharing of Results with Participants 22
8.0 Study Population 22
9.0 Vulnerable Populations 24
10.0 Local Number of Participants 24
11.0 Local Recruitment Methods 24
12.0 Withdrawal of Participants 27
13.0 Risks to Participants 27
14.0 Potential Benefits to Participants 33
15.0 Statistical Considerations 33
16.0 Confidentiality 36
17.0 Provisions to Monitor the Data to Ensure the Safety of Participants 37
18.0 Provisions to Protect the Privacy Interests of Participants 38
19.0 Compensation for Research-Related Injury 38
20.0 Consent Process 38
21.0 Setting 39
22.0 Multi-Site Research 40
23.0 Resources Available 41
24.0 References 42

ABBREVIATIONS/DEFINITIONS

- SMART/Sequential, multiple assignment, randomized trial
- PLUTO/Program on lung cancer screening and tobacco cessation
- PI/Principal investigator
- MTM/Medication therapy management
- NRT/Nicotine replacement therapy
- TLC/Tobacco longitudinal care
- NLST/National Lung Screening Trial
- LDCT/Low-dose computed tomography
- LCS/Lung cancer screening
- NCI/National Cancer Institute

1.0 Objectives

1.1 **Purpose**: Although previous clinical trials have demonstrated the efficacy of behavioral, pharmacological, and combination treatment strategies for smoking cessation, these treatments yield frustratingly low rates of long-term abstinence – in the range of 5-20%, depending on the population of smokers and intensity of treatment. This study uses a sequential, multiple assignment, randomized trial (SMART) design to compare adaptive interventions is a novel approach to evaluate smoking cessation intervention strategies. The chronic and relapsing nature of tobacco dependence makes the condition uniquely suited to this methodology. It incorporates stepping therapy up for those who continue to smoke with medication therapy management (MTM), led by a pharmacist, a tactic successfully applied to a number of complex chronic problems, but has not been formally tested for smoking cessation. This study also will provide formal testing of a step-down approach for those doing well with monthly versus every 3 month long-term counseling. This study also recruits from a previously untapped pool of participants – those who have agree to undergo a low-dose CT scan for lung cancer screening at one of the three participating sites. This study will enroll up to 1000 participants at the University of Minnesota, Minneapolis VA, and Allina Health.

2.0 Background

2.1 **Significance of Research Question/Purpose:** Although clinical trials have demonstrated the efficacy of behavioral, pharmacological, and combination treatment strategies for smoking cessation, these treatments yield frustratingly low rates of long-term abstinence – in the range of 5-20%, depending on the population of smokers and intensity of treatment. The emerging consensus is that long-term abstinence rates will improve only if tobacco dependence is treated as a chronic condition; yet chronic disease management models for smoking cessation are under-developed. Instead, researchers have focused on evaluating one-time treatment strategies delivered in isolation, leaving practitioners with scant guidance on how to manage smoking cessation treatment over time, including how best to tailor therapy based on patients' response to initial treatment. Are distinctive strategies useful when a smoker does not respond (at all or incompletely) to first-line treatment versus when a smoker does respond? For non-responders, should the same treatment be tried again, or should specific sequences of different treatment strategies be used?

Adaptive interventions are suited to conditions like tobacco dependence where patients vary in response to treatment, the effectiveness of an intervention may change over time, and relapse is common. Our team recently demonstrated the efficacy of one adaptive intervention for smoking cessation, Tobacco Longitudinal Care (TLC) that provides behavioral counseling and nicotine replacement therapy for one year, incorporating plans for smokers who have difficulty quitting or who quit and relapse. In this study the primary aim is to test whether the beneficial

effect of adaptive TLC treatment can be augmented by the strategic addition of pharmacist-assisted access to the prescription medications bupropion or varenicline (Medication Therapy Management). Because pharmacist-led MTM uses more resources and imposes greater burden on patients than TLC, it is critical to determine when and for whom this approach is most appropriate.

Testing adaptive interventions for smoking cessation treatment will fill a critical research gap. There is a large body of evidence to support specific behavioral and pharmacological interventions for smoking cessation.[2] The majority of evidence, however, comes from trials that test fixed interventions administered in isolation. Given the extraordinarily high rate of early relapse, especially soon after the onset of treatment (Figure 1),[1] critical decisions must be made about how to manage smoking cessation treatment over time, using a chronic disease management approach based on response to treatment, similar to the clinical management of hypertension or diabetes.[3] There is a remarkable gap in knowledge about how to manage the optimal sequence and intensity of smoking cessation interventions. For example: What is the best subsequent treatment for those who do not respond to first-line treatment? How long should we wait before deciding a smoker is not responding to treatment? How should the intensity of the intervention be stepped-up or stepped down? Should progress toward cessation on initial treatment be used to decide how to modify the intervention? How can the intervention be adapted over time to effectively address the specific and changing needs of the individual (e.g. response to treatment, lung cancer screening results)?

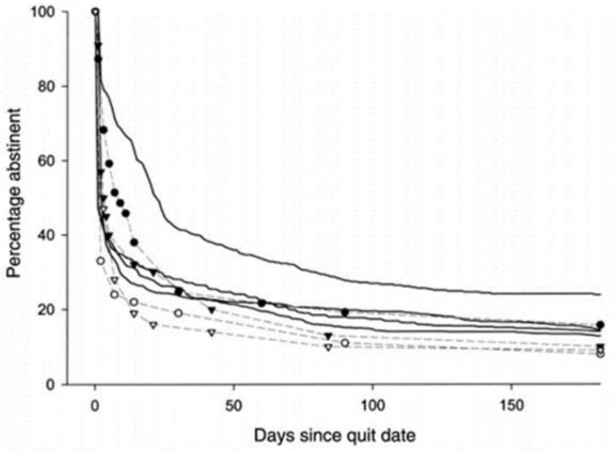

2.2 **Preliminary Data:** Adaptive interventions assign different treatment components to different individuals and use pre-specified decision rules to determine treatment dose and/or type.[4] The premise that intervention strategies need to adapt systematically to the changing needs and experiences of the individual provides a framework that closely resembles clinical practice. However, the development of adaptive intervention requires clear decision rules that explicitly identify the constructs used to tailor the treatment to an individual, as well as guidance regarding the implementation of the rules (e.g., how and when to measure the tailoring variables).[4] Sequential Multiple Assignment Randomized Trials (SMARTs) are designed to build optimal adaptive interventions.[5-7] A range of SMART projects that address questions in oncology, addictive disorders, and mental health treatment are listed on the Penn State University Methodology Center website.[8]

2.3 Existing Literature:

Chronic care is more effective than episodic care. A longitudinal care model reinforces that quitting may require an on-going series of attempts. A small number of published reports describe prolonged behavioral interventions for smoking cessation.[9-11]

Pharmacist-led smoking cessation interventions are effective. A range of health professionals, including pharmacists, are effective at delivery of smoking cessation treatment.[12-14] Pharmacist-led Medication Therapy Management (MTM) includes five core elements of service: medication therapy review, a personal medication record, a medication-related action plan, intervention and referral, and documentation and follow-up[15] that can be comprehensive or targeted, depending on the needs of the patient.[16, 17] MTM has been used successfully for patients with complex medical regimens[18, 19] and also for specific clinical situations where specialized care is needed, for example anticoagulation, asthma, and diabetes.[20]

In 2010 the National Cancer Institute completed the landmark National Lung Screening Trial (NLST), which showed that annual low dose CT screening decreased the risk of dying from lung cancer by 20%.[21-23] In response the American Cancer Society,[24] the National Comprehensive Cancer Network,25 the American College of Chest Physicians,[26] the American Society of Clinical Oncology,27 the American Lung Association,[28] and the US Preventive Services Task Force[29] issued recommendations in favor of screening for persons at high risk based on age and smoking history (Grade B). High risk is defined as adults aged 55-80 years who have a 30-pack-year smoking history and currently smoke or have quit within the past 15 years. The NLST and other screening studies suggest approximately 25% of patients will have an abnormal result that ranges from minor to severe. Epidemiological studies confirm the benefits of quitting smoking at any age, including in this older cohort30 and cost-utility analysis shows additional benefits of incorporating smoking cessation interventions with screening.[31]

If screening proves to be a “teachable moment”[32, 33] and amplifies the effectiveness of smoking cessation intervention, it has the potential to reduce mortality and morbidity through risk factor modification for other cancers, cardiovascular disease, and chronic lung disease. Those participating in screening may be more interested in quitting than average.[34]

Findings from several observational studies of smokers in lung cancer screening indicate meaningful interest in quitting (67-95%),[33-37] although there is variability by screening result and by study. For example, of current smokers in the NLST trial 70% considered quitting, 17% were preparing to quit, and only 13% had no intention to quit.38 In the NY Early Lung Cancer Action Project, 32% were seriously thinking of quitting within 30 days and 47% were seriously thinking of quitting within 6 months.[39] Smoking cessation rates among those undergoing lung cancer screening in studies are fairly high (16%-42%), but also vary by study.[35, 36, 39-46] Scan abnormalities are associated with a higher smoking cessation rate, supporting the premise that lung cancer screening may be an opportune time to promote cessation.[33, 35-37, 41, 42, 44, 47] Limited data do not support the concern that a negative scan reduces interest in cessation (“healthy certificate effect”).[40, 44] To date;

however, smoking cessation interventions in the lung cancer screening setting have relied on very low intensity treatment strategies such as the provision of self-help materials and referral to a quitline[43, 46, 48] and the vast majority of data are observational.

Conceptual Models. The adaptive interventions we propose are highly congruent with principles of chronic disease management.[3] Chronic disease management typically involves the patient, practice, and health care system, and relies on multidisciplinary teams that provide intensive follow-up.[49] Adaptive interventions are consistent with stepped care as a chronic disease management tool, as outlined in Figure 2. A stepped care model features 1) the least restrictive treatment for patients with respect to cost and personal inconvenience (i.e., first-line treatment), and 2) monitoring with correction.[50, 51] More intensive treatments are generally reserved for patients who do not respond to first-line treatments. Stepped care models often mimic clinic care and have been described for the case of smoking cessation[52, 53] as well as other behavioral disorders.[50, 51, 54, 55] Abrams et al suggested a stepped care approach using treatment matching that might bypass low-intensity intervention for smokers at high risk of relapse.[52] Stepped care relies on decision-making rules to define success and failure of treatment; however, there is a paucity of formal testing of such algorithms for the case of smoking cessation.

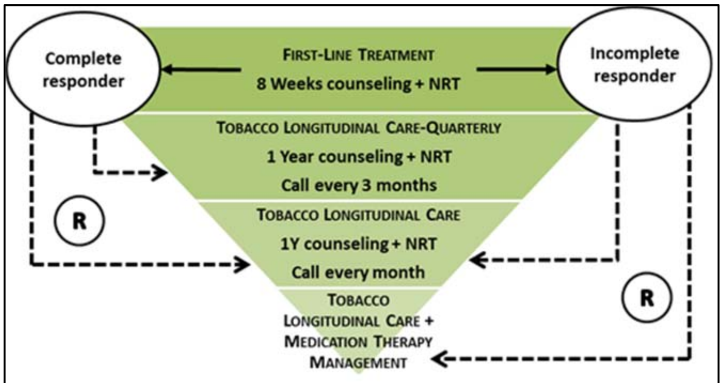

Figure 2. Adaptive intervention for smoking cessation treatment

The proposed set of interventions starts with a first-line treatment that has been shown to be both effective and efficient.[2, 56] Telephone coaching coupled with NRT, available over-the-counter, are the core components of many large scale effective smoking cessation treatment programs,[56] including TLC, that have the considerable advantage of no requirement for face-to-face contact and are relatively low cost. Decisions regarding subsequent treatment rest on how smokers respond to the first-line treatment. For those who are complete responders, treatment continues to involve counseling and NRT, but the frequency of follow-up could vary. For those who are incomplete responders treatment could continue to involve counseling and NRT or it could expand to include a pharmacist consult to provide prescription medications and medication therapy management. This SMART is designed to provide an evidence base to guide the decision rules that support treatment steps. The trial will also track psychological states that may underlie the effectiveness of the proposed intervention strategies (e.g., interest in quitting, cessation self-efficacy, satisfaction with cessation). Because the most intensive treatment includes the provision of prescription medications we will also examine the extent to which reductions in withdrawal[57] and urges to smoke[58] may help explain treatment effectiveness.

3.0 Study Endpoints/Events/Outcomes

3.1 **Primary Aim**: For incomplete responders (any smoking after quit date) to first-line treatment (8 weeks of counseling with nicotine replacement therapy), which strategy -- providing tobacco longitudinal care (TLC) for one year or adding Medication Therapy Management to TLC (TLC+MTM) – will result in higher rates of long-term abstinence (6 months prolonged abstinence measured 18 months after beginning of treatment)?

Hypothesis 1: Among incomplete responders, long-term abstinence rates will be higher in smokers randomized to TLC+MTM compared to TLC.

3.2 **Secondary Aims**:

Secondary Aim 1: For complete responders to first-line treatment, does monthly TLC contact (TLC) result in higher rates of long-term abstinence than quarterly TLC contact (TLC-Q)?

Hypothesis 2: Among complete responders, long-term abstinence rates will be higher in smokers randomized to TLC compared to TLC-Q.

Secondary Aim 2: Do the amount of treatment response and the timing of identifying incomplete/complete response to first-line treatment moderate the effect of different tobacco treatment algorithms?

Hypothesis 3a: Incomplete responders to first-line treatment who report more days smoking following the initial quit date will benefit more from TLC+MTM compared to TLC.

Hypothesis 3b: Complete responders identified at week 4 will benefit more from TLC compared to TLC-Q, but there will be no difference between TLC and TLC-Q for compete responders identified at week 8.

3.3 **Exploratory Aim:**

How do normal and abnormal lung cancer screening findings affect the smoking cessation process? Exploratory analyses using the standard ordinal scoring system for CT scan results (Lung-RADS) will examine relationships among screening results, engagement in treatment, and smoking outcomes.

4.0 Study Intervention(s)/Investigational Agent(s)

4.1 **Description:** This study uses a sequential, multiple assignment, randomized trial (SMART) design to compare adaptive interventions in a novel approach to evaluate smoking cessation intervention strategies. All participants receive first-line therapy (telephonic tobacco cessation counseling and are able to receive free nicotine replacement therapy (transdermal patches, gum, and mini-lozenges)). It incorporates stepping therapy up for those who continue to smoke with medication therapy management (MTM), led by a

pharmacist, a tactic successfully applied to a number of complex chronic problems, but has not been formally tested for smoking cessation. This study also will provide formal testing of a step-down approach for those doing well with monthly versus every 3-month long-term counselling.

4.2 **Drug/Device Handling:** NRT will be sent directly to participants from the University of Minnesota research team. NRT logs with purchase and receipt dates, lot numbers, expiration dates, product type, and dosage are retained by the University of Minnesota research team. Information about dispensing of medication (product, dose, dates) are recorded in REDCap in each participant's record.

Prescription smoking cessation pharmacologics (varenicline, bupropion, nicotine inhaler, nicotine nasal spray) may be prescribed per the protocol at the discretion of the pharmacist/physician assistant for patients randomized to MTM. MTM is pharmacist driven coordination of medications. The medications may include varenicline or bupropion, however these would be prescribed by the patient's (non-study) physician after evaluation by the pharmacist, not by the study. In contrast, nicotine replacement therapy will be arranged and provided by the study. Therefore, the study provides primary **responsibility** for the storage, dispensing, and **disposal** of the NRT used in this study, but not for any prescription smoking cessation pharmacologics.

Additional information about drug handling and prescription guidelines can be found in <u>Appendix C. Medication Therapy Management: Pharmacists Assisted Medication Therapy Guide</u>

4.3 **Biosafety:** This study does not involve a recombinant or synthetic nucleic acid, human gene transfer, biologically derived toxin or an infectious agent.

4.4 **Stem Cells:** This study does not involve the use of human- or animal-derived stem cells.

5.0 Procedures Involved

5.1 **Study Design:** This study employs a prospective sequential, multiple assignment, randomized trial design.

5.2 **Study Procedures:** The intervention period will be 12 months for all participants, regardless of whether they respond to treatment or not, and regardless of randomized treatment group(s). The focus of all counseling will be to develop a personalized quit plan and use a combination of behavioral and pharmacological therapy to completely stop smoking, but the frequency of coaching, type of medication, and timing of identifying treatment response as complete/incomplete will vary by randomized treatment group.

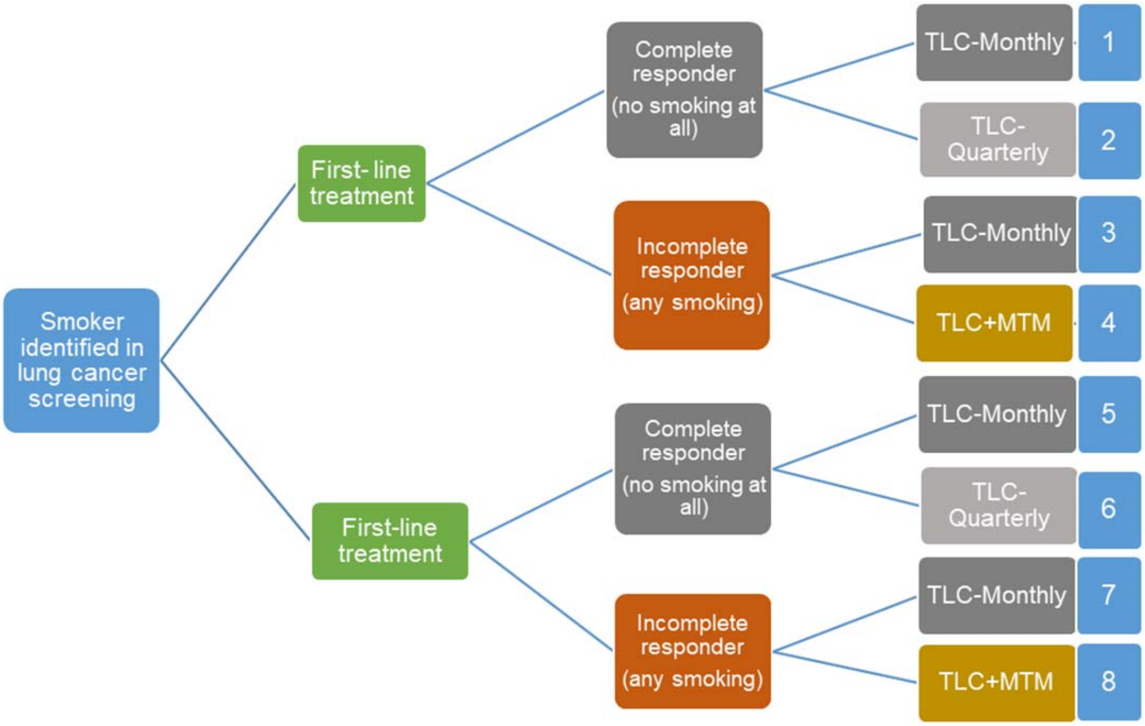

Counseling will begin before lung cancer screening takes place whenever possible, based on preliminary findings that intervention prior to CT scanning is more effective.[43] During the outreach call counselors will ask participants to set a quit date. For those who set a quit date within 30 days the first-line treatment algorithm will begin. Counselors will call those who do not set a quit date within 30 days every 2 weeks during 12 weeks from the time of enrollment to select a quit date.

5.2.0 Participant Registration and Randomization

Registration to the study will occur after the subject consent is signed and eligibility is confirmed, but before any study related procedures.

Participants will be assigned to treatment groups using computer generated blocked (within site) randomization schemes for each potential randomization, available on a secure Web-based application (REDCap). The first randomization will occur 30 days (-2/+14 days) after the first telephone call from the counselor to the participant (outreach call) during a regularly scheduled call.

For Secondary Aim 2, participants will be randomized to identification of complete/incomplete response to first line treatment at 4 weeks vs. 8 weeks. During the 4- or 8-week call, the counselor assessment of smoking status will be linked to the appropriate randomization scheme (TLC vs. TLC-Q for responders, TLC vs TLC+MTM for incomplete responders). This sole element of data collection by the counselor is designed to streamline the treatment sequence and avoids the logistical challenge of requiring separate but coordinated counselor and data collector calls at these critical treatment decision points.

Participants who are complete responders to first-line treatment (i.e. no smoking at all after a two week grace period after the quit date) will be randomized to TLC or TLC-Q at either 4 weeks or 8 weeks.

5.2.1. First-Line Treatment: 8 weeks (all participants)

In first-line treatment seven counseling calls will be scheduled during the first 8 weeks using a front-loaded, relapse sensitive schedule shown to be effective in other trials74 at the following time-points: outreach, pre-quit, 1-3 days post-quit, and 1, 2, 4 and 8 weeks. Refer to Appendix A: Telephone Smoking Cessation Counseling – First-Line Therapy Call Guide for detailed information on this component of the study. The outreach call will last 30-60 minutes and include confirming the quit date, and choosing nicotine replacement therapy (NRT). Call content will include problem-solving, skills training, social support, NRT use, and relapse prevention.

NRT monotherapy (patch, gum, or lozenge) or NRT combination therapy (patch + gum or patch + lozenge) will be available (free, delivered by mail). NRT choices will be modeled on common clinical practice and based on the principles that participants:

1) receive NRT unless they have a contraindication, and
2) will choose NRT type based on intensity of withdrawal symptoms, past experience, and participant preference.

Abnormal lung cancer screening results may redefine participant smoking as having a critical role in determining their health, a key construct thought to motivate individuals to adopt risk-reducing health behaviors.75 Considerable evidence supports this sort of personalized messaging for smoking cessation in the context of lung cancer screening. Counselors will incorporate this feature in the protocol, using a patient-centered approach.76 For example, they will ask smokers if CT scan results have become available, about results, and inquire about potential need for follow-up diagnostic studies. They will also ask about the participant’s perception of risk related to smoking before and after learning about screening results, and how the results change their feelings about smoking. Counselors will provide education regarding scan findings that are likely related to smoking, e.g. emphysema. They will emphasize that a negative screening result does not mean the participant is not at risk from smoking, and that it is better to quit smoking before developing any screening abnormalities.

5.2.2. Randomization to Long-Term Intervention

During the 4 or 8 week first-line therapy call (as assigned at the initial randomization), the counselor assessment of smoking status will be linked to the appropriate randomization scheme:

Participants who are complete responders to first-line treatment (i.e. no smoking at in the 7 days prior to the call) will be randomized to Tobacco Longitudinal Care (TLC) or TLC-Q. In TLC-Q, counselors will call participants every 3 months (rather than every month).

Participants who are incomplete responders to first-line treatment (i.e. continue to smoke) will be randomized to either TLC alone or TLC with Medication Therapy Management (MTM).

Those assigned to assessment of response to treatment at 4 weeks will undergo the second randomization to long-term intervention during a regularly scheduled call within 28-44 days of their outreach call. Those assigned to assessment of response to treatment at 8 weeks will undergo the second randomization to long-term intervention during a regularly scheduled call within 56-74 days of their outreach call.

5.2.3. Tobacco Longitudinal Care (TLC): 12 months

TLC is based on a chronic care model for tobacco treatment, including recycling treatment tools that worked in the past and relapse prevention. Chronic disease management includes continuity of care,77 stepped care improvement (vs. extinction) of symptoms, and self-management. The TLC protocol used in our prior trial11 includes contingencies for whether participants are abstinent, have relapsed (but to a reduced number of cigarettes), have resumed the original amount of smoking, or have not quit at all. Refer to Appendix B. Tobacco Longitudinal Care for complete details.

For Participants Who Are Abstinent

Counselors will use relapse prevention strategies including 1) identification of future high risk situations and skills to handle them; 2) methods to maintain abstinence such as lifestyle changes; 3) building self-efficacy and social support; and 4) issues of weight and exercise.

If during the call it is determined that a participant assigned to the TLC-Q schedule is smoking, the participant will switch to the TLC schedule.

For Participants Who Relapse

For participants who relapse (this is anticipated to be a large proportion of our sample over the one-year treatment period), counselors will urge them to make a new quit attempt as soon as they are willing. If participants are not interested in making another quit attempt, counselors will explain that smoking reduction is an alternative, and provide positive reinforcement for this choice as a step toward quitting. Counselors will stress a goal of at least 50% reduction from the baseline amount of cigarettes smoked (and less than 15 cigarettes per day), emphasizing that more reduction is better than less at any level of smoking. Strategies such as delaying smoking, eliminating cigarettes in specific

situations, and scheduled reduction will be discussed. For participants choosing smoking reduction, counselors will aim to call every 2 weeks, but the schedule will be determined by participants' progress and receptivity to phone sessions.

For Participants Who Choose to Neither Make A Quit Attempt Nor Reduce Smoking

For participants who choose to neither make a quit attempt nor reduce smoking, calls will be made monthly and they will be asked at each contact if they want to set a quit date or reduce. The counselor will also use motivational interviewing techniques with consideration for the participants' individual concerns about quitting78, 79 and target cognitive factors to promote setting a quit date, such as self-efficacy and outcome expectations.

NRT can be used for repeat quit attempts or to maintain smoking reduction. Participants who continue to smoke can use the same NRT as when they tried to quit, use an alternative form of NRT, or increase the dose of NRT. Choices between these options will be based on past experience, intensity of nicotine withdrawal symptoms, and smoker preference. NRT recommendations for reducers include 1 piece of 4 mg gum to substitute for one cigarette, or a 21 mg. patch (for those who smoke more than 10 cigarettes per day).

5.2.4. Medication Therapy Management (MTM): 12 Months

MTM services will expand the toolbox available in TLC to include in-person consultation with a pharmacist, the prescription drugs bupropion or varenicline, or combination medications (combination NRT or bupropion + NRT). The participant is not obliged to use these medications, but the pharmacist will assess readiness to quit and promote their use (Appendix C. Medication Therapy Management).

The first pharmacist-participant encounter will occur in the clinical setting and will follow an algorithm that includes a systematic process of collecting patient and medication-related information such as past experiences with medications and contraindications. The initial pharmacist-participant encounter may occur by telephone if the participant is unable to travel to the clinic. The pharmacist will follow a protocol to develop a patient-specific medication action plan. The pharmacist will have MTM certification from the American Pharmacist Association and can prescribe medications under a collaborative practice agreement. The action plan includes a list of self-management actions necessary to achieve the patient's specific health goals that will be congruent with TLC counselor recommendations. The participant and the pharmacist will record actions and track progress towards health goals (the participant on paper forms, the pharmacist in the EMR).

Within 2 weeks of randomization to MTM, the TLC counselor will notify the participant by telephone and mail of allocation to MTM. Additionally, the MTM pharmacist will be

provided with the names and contact information of participants randomized to MTM and will attempt up to 3 contacts by telephone to establish communication with the participant. The goal is for the first visit to occur within 2 weeks of randomization to MTM and and typically last about one hour (however some participants may decline referral). There can be a range of number of follow-up calls (usually 3-6 calls). Follow-up calls are typically 5-10 minutes and are conducted over a 12-week period, but can last until the end of the 12-month treatment period depending on participant interest and progress. The TLC counselor will continue to interact with the participant by telephone during MTM, but the pharmacist will have responsibility for medications while they work with the participant. Counselors can access the EMR to read notes related to MTM visits/calls.

A parking validation ticket will be given to participants who park at the Clinics and Surgery Center for their MTM visit.

5.2.5. Biomarker Validation: 18 Month Follow-Up

Participants who report 6 months abstinence at 18-month follow up will be asked to submit a saliva sample for biochemical verification (additional $50 Target gift card for collection). Within one month of the final assessment a saliva kit will be mailed to the participant for home collection (section 5.2.7a. Salivary Cotinine Collection).

Saliva samples will also be requested from a random sample of non-quitters to estimate biochemical validation nonresponse bias.

Saliva samples will be analyzed for cotinine in Dr. Sharon Murphy's lab in the UMN Masonic Cancer Center. We will use methods that have demonstrated a yield greater than 80% rate of return at 12 months in the past.[106]

Due to the COVID-19 public health crisis, saliva samples will not be collected to minimize potential risk to subjects and research staff (see section 13.4 for additional COVID-19 risk mitigation details). The iCO™ Smokerlyzer® will be used as an alternate method of verifying tobacco abstinence. The iCO™ Smokerlyzer® is a handheld device that connects to a smartphone and uses an interactive smartphone application to collect a breath test to provide instant biofeedback on exhaled carbon monoxide (CO) levels. Exhaled CO is an effective measure of nicotine consumption and is a recommended test for biochemically confirming abstinence. The protocol for iCO™ Smokerlyzer distribution and collection is located in section 5.2.7b.

5.2.6. Measures and Data Collection Schedule

Data sources will include telephone surveys, the EMR (consent will be obtained from participants), counselor records, and saliva (to verify abstinence). Based on prior experience we will use financial incentives ($25 Target gift card for baseline, $20 Target gift card for other time points, and a two dollar bill pre-incentive prior to the final data

collection call). Follow-up data collection will be anchored by the outreach call. Short windows around each time point will maximize response rates. We will collect multiple phone numbers, consent to text, email addresses, and contact information for two family members or friends to maximize retention. The study team has accomplished retention rates in the 70-90% range in past clinical trials of similar duration.[11, 59, 61, 63, 82] **Table 2** summarizes demographic, psychological, and outcome data assessments.

Table 2. Measures and data collection schedule

	T0	4W	8W	12W	6M	12M	18M	Data source	Instrument
**Demographics**									
Age, gender, race, education, income	x							Survey	NHIS[83]
**General health**									
Physical Health	x					x	x		SF-12
Medical co-morbidities	x							Survey	Charlson Index[84]
Pain	x								PEG
COPD	x			x		x	x		
**Mental health and alcohol use**									
Depression	x								PHQ-9
Anxiety	x							Survey	GAD-7
Alcohol use	x								AUDIT-C[85]
**Smoking history**									
Tobacco use: all products including e-cigs	x	x	x	x	x	x	x	Survey	PATH[88]
Cigarette dependence	x								CDS-5
**Social support**									
Social Support: Partner Interaction Q	x	x	x	x	x	x	X	Survey	Mermelstein 1986[93, 94]
**Personality**									
Regulatory focus	x							Survey	Regulatory Focus Scale
**Smoking cessation-related beliefs and states**									
Interest in quitting	x	x	x	x	x	x	x		Readiness to Quit Ladder
Nicotine Withdrawal	x	x	x	x	x	x			Hughes[57, 90, 91]
Smoking Urges		x	x	x	x	x	x	Survey	CSU-B
Self-Efficacy for Smoking Cessation	x	x	x	x	x	x	x		Baldwin 2006[67]
Satisfaction with cessation		x	x	x	x	x	x		Baldwin 2006[67]
**Treatment utilization**									
Counseling (number & duration of calls)		x	x	x	x	x		Counselor	
MTM sessions (number & duration of in-person visits and calls)		x	x	x	x	x		Pharmacist	**Appendix E.**
NRT and medication prescription		x	x	x	x	x	x	Counselor, pharmacist	**Tobacco Treatment Form**
Medication adherence, adverse reactions		x	x	x	x	x		Survey	TLFB,[98] Marteau 2012[99]
Treatment satisfaction							x		May,[100, 101]
**Smoking outcomes**									
7-day, 30-day point prevalent abstinence		x	x	x	x	x	x	Telephone	BRFSS,[102] NHIS,[83]
6M prolonged abstinence					x	x	x	Survey	Hughes[103, 104]
Relapse, slip		x	x	x	x	x	x		
Cotinine verification smoking abstinence							x	Saliva	Murphy et al[105]
**Lung cancer screening outcomes**									
CT scan results		x	x	x	x	x			
Diagnostic work-up components		x	x	x	x	x	x	EMR	Lung-RADS Version 1.0 Assessment Categories[72]
Screening outcomes/diagnoses		x	x	x	x	x	x		
**Perceptions of cancer risk**									
Self-assessment of cancer risk	x	x	x	x	x	x	x	Survey	Hahn 2006,[34] Park 2009[38]
**COVID-19 Impact**									
Perceived risk and diagnosis questions					x	x	x		
**Data Collection Windows**									

Table 2. Measures and data collection schedule

	T0	4W	8W	12W	6M	12M	18M	Data source	Instrument
Range anchored to outreach call completion call date (days)	No limit	-4/ +14	-4/ +14	-4/ +14	-7/ +21	-7/ +21	-7/ +21	NA	NA

Research assistants involved in data collection will be blind to treatment assignment and will not have counseling responsibilities.

A brief written survey will be mailed to participants who miss their 12 month or 18 month telephone data collection. A pre-addressed and postage paid return envelope will be provided for the participant to return the survey to the study team.

Lung cancer screening outcomes and medication therapy management outcomes data will be retrieved from the EMR. These data will be extracted from the EMR in collaboration with the AHC-IE. Missing data may be obtained via manual chart review by a trained member of the research team.

The impact of the 2019 novel coronavirus (COVID-19) public health crisis to participants enrolled in this study is unclear. Participants will be asked about the impact of COVID-19 on their smoking behaviors and whether they have been diagnosed or otherwise believe they have had COVID-19.

5.2.7a Salivary Cotinine Verification

Due to the COVID-19 public health emergency, saliva sample collection is on temporary hold.

A. Research assistant to mail cotinine sample collection package to reported quitters (18-month follow-up data collection)

1. Label cotinine collection tube; mark the date and the participant's study identification number on the label.
2. Address the envelope to the study Participant.
3. Insert the labeled tube, a self-addressed return envelope, and the thank-you/instructions letter.
4. Mail to study participant.
5. Log the information for tracking.

B. Follow-up to cotinine sample collection package

- If no cotinine testing kit has been returned to the testing laboratory in 2 weeks, 3 call attempts will be made to reach the participant.
- If there is no answer on the 1st and 3rd call attempts, a voice mail message is left for each call.

C. Receipt of package

1. Send package to Dr. Murphy's lab as soon as it is received.
2. If the testing laboratory deems the sample inadequate, the RA will send a new one with special instructions.
3. After confirming that the sample was adequate, the RA will send payment to participant.

D. Documentation in REDCap:

Documentation data should include:
Participant ID
Participant Name
Participant Address
Date package is received by study
Date compensation is mailed
Results of lab analysis

E. Script for Saliva Cotinine Sample Collection Protocol

Biochemical validation of self-reported 7-day abstinence will be obtained at 18 months using a mailed-in saliva sample that will be tested for the nicotine metabolite, cotinine.

1. When a research participant reports at the 18-month follow-up that he/she has not smoked a single cigarette, even a puff, in the last 7 days, the RA follows this script:

"Would you be willing to give us a sample of your spit so that we can document that you do not smoke now? If you would do this, it would be very helpful to us. We will be testing the spit samples for cotinine, which is a chemical that your body makes from nicotine. This test will show only your exposure to cigarette smoke. It will not test for any other chemicals than cotinine. If you would do this, you will receive $50. Would you like me to tell you how this works? "

If no, say "thank you" and continue with the standard script.

If yes, describe what happens next:
"We mail you a cotton swab. You fill your mouth with spit. Then place the swab in your mouth. Keep it in your mouth for about 20 seconds, moving it around, and chewing on it until it is soaked with spit. Then you put it back in the tube it came in. Then you put the swab in the tube in the envelope that came with the swab, and drop it in the mail. We want to emphasize how important it is that this sample comes from you, not anyone else. You will be compensated if we receive the sample, regardless of whether or not the test result is positive. As soon as we receive it, we will load $50 to your study gift card."

"Okay. Thank you."

"These materials will go out to you today in the mail. The envelope you receive will have the swab and directions for how to use it."

"If you have any questions when using the swab, please call us at 1-844-389-5619. Thank you for participating."

5.2.7b. Exhaled CO Verification

A. Script for Breath Test Kit

Research assistant to send iCO™ Smokerlyzer® (i.e., "breath test kit") to participants who would normally have been selected to provide a saliva sample. Participants must provide verbal consent prior to the research assistant mailing the breath test kit. Verbal consent will be documented in REDCap.

During the 18-month follow-up call, if the participant is selected to provide a saliva sample, the research assistant will follow this script:

> "When you originally joined the study, we explained that you may be asked to provide a saliva sample at the end of the study. The purpose of the saliva sample is to test your spit for a marker of nicotine presence. Because of the new coronavirus, also known as COVID-19, we are not currently collecting saliva samples.
>
> We can test you for nicotine presence using a different method. We can send you a breath test kit to see if you have nicotine in your body. The breath test kit has a device called a Smokerlyzer that you breathe in to. You connect the Smokerlyzer device to your phone and download an application on your phone, then breathe in to the device. The application on your phone will send us information about the level of carbon monoxide you are breathing out. We will send you a $50 Target gift card when we receive your breath test results.
>
> Would you be willing to do a breath test?
>
> > If yes: Can you confirm that you have a smartphone and would be able to download apps? (*If not, we will not be able to send them a breath test kit).* Does your smartphone have a standard (round) headphone jack? (*If not, they will need an adapter for the newer iPhone/Android model, which we can provide with the kit*).
> > If no: I understand (*proceed with call per usual*).

B. Mailing the Breath Test Kit.

6. Breath test kit will include the iCO™ Smokerlyzer® device and the iCO Instruction Infographic
7. Address the envelope to the study participant.
8. Mail to study participant.
9. Log the information in REDCap for tracking.

B. Follow-up to mailing breath test kit

- If results from the breath test kit have not been received by the study team within 1 week of mailing the kit, 3 call attempts will be made to contact the participant.
- If there is no answer on the 1st and 2nd call attempts, a voice mail message is left for each call.

5.2.8. End of Treatment Procedures

After 12 months of treatment, TLC counselors will notify participants of termination of treatment by mail (see: PLUTO UMN End of Treatment Letter). For patients who seek additional resources for smoking cessation outside of the study, a resource brochure will be provided (see: UMN Smoking Cessation Resource Brochure in Supplemental Materials).

5.3 **Study Duration:** The anticipated duration for individual participation in the study is 18 months from the date the Outreach Call is completed. The duration anticipated to enroll all study participants is 2 years (September 2016 to August 2019). The duration anticipated to complete all study procedures, including long-term follow-up and data analysis, is 4 years (September 2016 to August 2021).

5.4 **Individually Identifiable Health Information**: This study involves the use of individually identifiable health information.

5.5 **Use of radiation***:* This study does not involve the use of radiation.

5.6 **Use of Center for Magnetic Resonance Research***:* This study does not utilize Center for Magnetic Resonance Research facilities.

6.0 Data and Specimen Banking

N/A.

7.0 Sharing of Results with Participants

This study does not involve diagnostic or genetic tests which could lead to the discovery of incidental findings that would need to be shared with the participant and/or the participant's medical team. This study is registered on ClinicalTrials.gov under identifier no. NCT02597491.

8.0 Study Population

8.1 Inclusion Criteria:

- Scheduled or has order for low dose CT screening for lung cancer, has previously participated in low dose CT screening for lung cancer, or is eligible for low dose CT screening for lung cancer but does not have scheduled exam or order
  - If prior low dose CT screening for lung cancer is reported, confirmation will be elicited from the patient's medical record to ensure patient received screening exam (versus imaging for evaluation of a medical problem, e.g., lung cancer work-up).
- 30 pack-year or greater smoking history

- Current daily smoker; eligible smokers will have smoked during the past 30 days and on at least 5 out of the past 7 days
  - Individuals who have made a recent quit attempt (for example, in anticipation of their low dose CT scan for lung cancer screening) will be eligible if during the last 30 days, when they were smoking regularly, they smoked on at least 5 out of 7 days of the week.
- 55 to 79 years of age
- Interested in quitting and willing to choose a quit smoking date within the next 12 weeks
- Voluntary written consent

8.2 Exclusion Criteria:

- Unstable psychiatric disease, unless stable in treatment for 3 months (smokers on mental health medication with any changes in medication in past 3 months require study MD approval to participate) - Smokers with stable psychiatric disease will be eligible; this baseline characteristic and related symptoms will be considered in analyses.
- No hospitalization for mental health reasons in past 3 months; No thoughts of self-harm in past 2 weeks
- No recent cognitive impairment (difficulties planning or organizing daily activities, such as managing finances, having trouble remembering appointments, or forgetting the correct month of the year); participants reporting recent cognitive impairment will be given the 6-item Callahan Cognitive Screener and must score at least a 5 out of 6 to participate
- Participating in a current formal quit program (such as tobacco cessation counseling or working with a pharmacist, with or without use of NRT, bupropion, or varenicline).
  - Smokers using NRT will be eligible as long as they are not using it as part of a formal quit program
- Smokers using bupropion or varenicline for smoking cessation will be ineligible if they began their course of treatment within 8 weeks of the date of eligibility screening. For smokers using bupropion or varenicline for smoking cessation for greater than 8 weeks at time of eligibility screening, The standard course of treatment with bupropion or varenicline is 8 weeks. For smokers determined to be eligible, but who have been using bupropion or varenicline for smoking cessation for greater than 8 weeks at the time of eligibility screening, a study physician will communicate with the patient's prescribing physician to facilitate transition off bupropion or varenicline.
- No phone
- Non-English speaking
- Current diagnosis of lung cancer if lung cancer was diagnosed prior to study-qualifying LDCT (patients who report diagnosis of lung cancer will be assessed for eligibility by a study MD)

8.3 **Screening:** Trial recruitment will be fully coordinated with the lung cancer screening programs at the both the UMN, VA, and Allina Health sites. In coordination with Fairview Research Administration, patients will also be recruited from Fairview community clinics.

Patients will be screened by research assistants (screening questions can be found in the Data Collection Forms in the Supplemental Materials) to determine eligibility.

Individuals who are deemed ineligible will be asked for permission to retain their name and contact information for the purposes of future re-contact in the even that eligibility criteria changes in the future. No information would be retained on individuals who do not give permission to do so.

9.0 Vulnerable Populations

9.1 Vulnerable Populations:

☐ Children
☐ Pregnant women/Fetuses/Neonates
☐ Prisoners
☐ Adults lacking capacity to consent and/or adults with diminished capacity to consent, including, but not limited to, those with acute medical conditions, psychiatric disorders, neurologic disorders, developmental disorders, and behavioral disorders
☐ Approached for participation in research during a stressful situation such as emergency room setting, childbirth (labor), etc.
☐ Disadvantaged in the distribution of social goods and services such as income, housing, or healthcare
☐ Serious health condition for which there are no satisfactory standard treatments
☐ Fear of negative consequences for not participating in the research (e.g. institutionalization, deportation, disclosure of stigmatizing behavior)
☐ Any other circumstance/dynamic that could increase vulnerability to coercion or exploitation that might influence consent to research or decision to continue in research
☐ Undervalued or disenfranchised social group
☐ Members of the military
☐ Non-English speakers
☒ Those unable to read (illiterate)
☐ Employees of the researcher
☐ Students of the researcher
☐ None of the above

9.2 **Additional Safeguards:** Patients who meet all study criteria but whom are unable to read may (illiterate) may be enrolled in this study. The consent process is modified for these individuals: (1) The consent conversation must be witnessed by a non-member of the research team; (2) The consent form must be signed by the witness to the consent process

10.0 Local Number of Participants

10.1 Local Number of Participants to be Consented: 750

11.0 Local Recruitment Methods

Recruitment Process: Recruitment is fully coordinated with the MHealth Lung Cancer Screening Program, Fairview Research Administration, and AHC-IE. An easy-to-remember mnemonic was developed for recruitment and Participant use for this study. For all Participant-related correspondence, the trial will be referred to as "PLUTO," or, the

"Program on Lung Cancer Screening and Tobacco Cessation." Internally, the trial will remain titled as stated on the IRB application and original protocol: "Adaptive Interventions for Smoking Cessation in Lung Cancer Screening Programs."

Recruitment Mechanism #1

Patients who meet criteria for lung cancer screening based on age and smoking history will be identified from the EMR and mailed invitations for lung cancer screening (not part of the research protocol but part of the clinical care protocol for lung cancer screening). Those patients will receive information about the trial in a separate letter. This information will emphasize that smoking cessation is an integral part of the lung cancer screening program (whether or not smokers enroll in the trial). They will be invited to call a toll-free number if they are interested in participating to learn more about the study. We will follow up the invitation letter by telephone. A phone script for potential study Participants can be found in the Supplemental Materials. If during the follow-up recruitment call it is determined the potential participant is not currently participating in lung cancer screening, the patient will be encouraged to speak with their primary care doctor about lung cancer screening. A follow-up recruitment letter will be mailed to the patient which encourages the patient to speak to their doctor about lung cancer screening. Eligibility criteria include patients who are eligible for lung cancer screening, both those who complete the scan and those who do not. All patients will receive information about lung cancer screening at the time of enrollment (see: Supplemental Materials). Because of the annual recommendation for lung cancer screening, a subset of patients who received an initial lung cancer screening invitation and PLUTO recruitment follow-up (e.g., those who expressed interest during initial recruitment contact but never pursued lung cancer screening or those who were never reached) will be re-contacted by the study team, if they have not opted out of research in the interim.

Recruitment Mechanism #2

Patients referred by a physician for a Lung Cancer Shared Decision Making Visit or Lung Cancer Screening CT will also be identified from the EMR and receive the study invitation letter. If there is insufficient time to mail the study invitation letter, patients may be approached for recruitment at the time of their Shared Decision Making Visit or Lung Cancer Screening CT. The AHC-IE will provide a list of patients who have had an order placed for a lung cancer screening CT; these patients will also receive a study invitation and follow up by telephone.

Recruitment Mechanism #3

This recruitment mechanism will be fully coordinated with the M Health-Fairview Lung Cancer Screening Program, the AHC, and the Fairview MyChart Governance Group. Two pools of patients will receive MyChart messages inviting them to participate in the study. Pool 1 patients are patients who have previously been contacted by our research team who were either (1) never reached or (2) were not interested in quitting smoking within 3

months but who did not otherwise decline to participate at that time. Pool 2 patients are patients who are currently participating in the M Health/Fairview Lung Cancer Screening Program. The MyChart message will include information about the study with options for the patient to either call the study team or to click on an embedded, secure, REDCap link (https://redcap.ahc.umn.edu/surveys/?s=TKY7J7P4TM) to complete an initial screener to determine if the patient may be eligible. The initial screener includes up to 9 questions and upon completion of the screener, the patient will be notified in their web browser whether they may or may not be eligible. Patients who may be eligible will be contacted by the study team to complete the remaining eligibility screening questions. The MyChart message and screen captures of the REDCap questionnaire are included in the Supplemental Materials.

11.1 **Identification of Potential Participants:** Patients who meet criteria for lung cancer screening based on age and smoking history will be identified from the EMR and mailed invitations for lung cancer screening (not part of the research protocol but part of the clinical care protocol for lung cancer screening). Those patients will receive information about the trial in a separate letter. This information will emphasize that smoking cessation is an integral part of the lung cancer screening program (whether or not smokers enroll in the trial). They will be invited to call a toll-free number if they are interested in participating to learn more about the study. We will follow up the invitation letter with a telephone call prior to the scheduled lung cancer screening visit. A phone script for potential study Participants can be found in the Supplemental Materials. If during the follow-up recruitment call it is determined the potential participant is not currently participating in lung cancer screening, the patient will be encouraged to speak with their primary care doctor about lung cancer screening. A follow-up recruitment letter will be mailed to the patient which encourages the patient to speak to their doctor about lung cancer screening (Supplemental Materials).

11.2 **Recruitment Materials**: A recruitment letter will be mailed to patients identified by the EMR as potentially eligible for participation (Supplemental Materials). A MyChart message will be sent to a subset of potential participants (Supplemental Materials).

11.3 **Payment**: Participants will be compensated with Target gift cards according to the compensation schedule:

**Data Collection Event**	**Compensation Amount**
Baseline data collection assessment	$25 Target gift card
Week 4 data collection call	$20 Target gift card
Week 8 data collection call	$20 Target gift card
Week 12 data collection call	$20 Target gift card
Week 26 data collection call	$20 Target gift card
Week 52 data collection call	$20 Target gift card
Week 78 data collection call	$20 Target gift card
Saliva sample mailed in	$50 Target gift card

Total potential compensation:	$195 in Target gift cards

Participants will be mailed a Target gift card after each data collection event. Gift card payment will be prorated based on completion; participants will need to complete at least part of their data collection call to be eligible to receive compensation. All participants who return a saliva sample, regardless of smoking status, will be mailed a gift card. Participants will need to complete at least part of the data collection encounter to be eligible to receive compensation for that data collection encounter. Participants will not be eligible to receive compensation for any data collection calls or saliva sample after the date of withdrawal. Target gift cards will be mailed directly to the participant. Research Experience Points will not be awarded for this study.

12.0 Withdrawal of Participants

12.1 **Withdrawal Circumstances**: Participants may be withdrawn in the event of unforeseen medical circumstances (e.g., significant change in mental health requiring hospitalization) at the discretion of the Principal Investigator. Participants may also withdraw their participation from the study at any time.

12.2 **Withdrawal Procedures**:

Participants who elect to withdraw from the study will be asked to continue participating in the data collection assessments. If the participant elects to withdraw from both the intervention and data collection assessments, the participant will be mailed a letter to confirm they have been withdrawn from the study (Supplemental Materials). Participants who withdraw will also receive the end-of-study resource brochure with information about additional smoking cessation resources. Data will be collected from the patient's medical record during the on-study timeframe.

12.3 **Termination Procedures**: In the event that the PI determines a participant must be withdrawn, the research team will attempt to communicate this to the participant at the earliest available opportunity. The research team will follow up this communication by sending a letter to the patient to confirm they have been withdrawn from the study (Supplemental Materials). Participants withdrawn by the PI will also receive the end-of-study resource brochure with information about additional smoking cessation resources (Supplemental Materials). Data will be collected from the patient's medical record during the on-study timeframe.

13.0 Risks to Participants

13.1 **Foreseeable Risks:**

Quitting or cutting down on nicotine in cigarettes can lead to nicotine withdrawal symptoms that may last up to two weeks. Although withdrawal symptoms may be unpleasant, they do not pose a serious health risk. Nicotine withdrawal symptoms can include insomnia, restlessness, anxiety, difficulty concentrating, depressed mood, increased

appetite, and decreased heart rate. Other common withdrawal symptoms include headache, nausea, constipation, and or diarrhea.

Side effects from the nicotine gum and lozenge include mouth and throat irritation and sores, hiccups, stomach aches, diarrhea, and heartburn. These side effects are more likely to occur if the lozenges or gum are chewed quickly. Participants will receive instruction on how to use the gum or lozenge properly. Side effects of the nicotine patch are sleep problems or abnormal dreams, and redness, itching or swelling at patch site. Less frequent side effects may include headache, palpitations, nausea/vomiting, stomach aches, lightheadedness or dizziness. Participants will be encouraged prior to beginning nicotine replacement therapy to contact their tobacco cessation counselor if they have any side effects; participants reporting side effects will work with their tobacco cessation counselor to adjust or discontinue nicotine replacement therapy use. Attempts to promote smoking cessation may create frustration. There are some risks of loss of confidentiality.

The NLST (n = 53,456) reported deaths relating to neoplasms of the bronchus and lung (1.7%), other neoplasms (1.6%), cardiovascular illness (1.9%), and respiratory illness (0.7%). In this study of 55-79 year olds with a heavy smoking history (>30 pack-year), we anticipate deaths unrelated to the study intervention may occur subsequent to the risk of cancer and smoking-related illness in this population. All participant deaths will be reviewed by the PI (Anne Joseph, MD, MPH) for their relatedness to the study intervention. Any deaths that are possibly related will be promptly reported the UMN IRB per the HRP Investigator Manual guidelines.

<u>Suicide Risk Management Protocol</u>

Anxiety and depression are known to be comorbid with tobacco use and are monitored for their potential moderating effects in this study. In the event suicidal ideation and behavior is disclosed to the research team, we will use a Suicide Risk Management Protocol to assess and mitigate risk.

[Figure on next page]

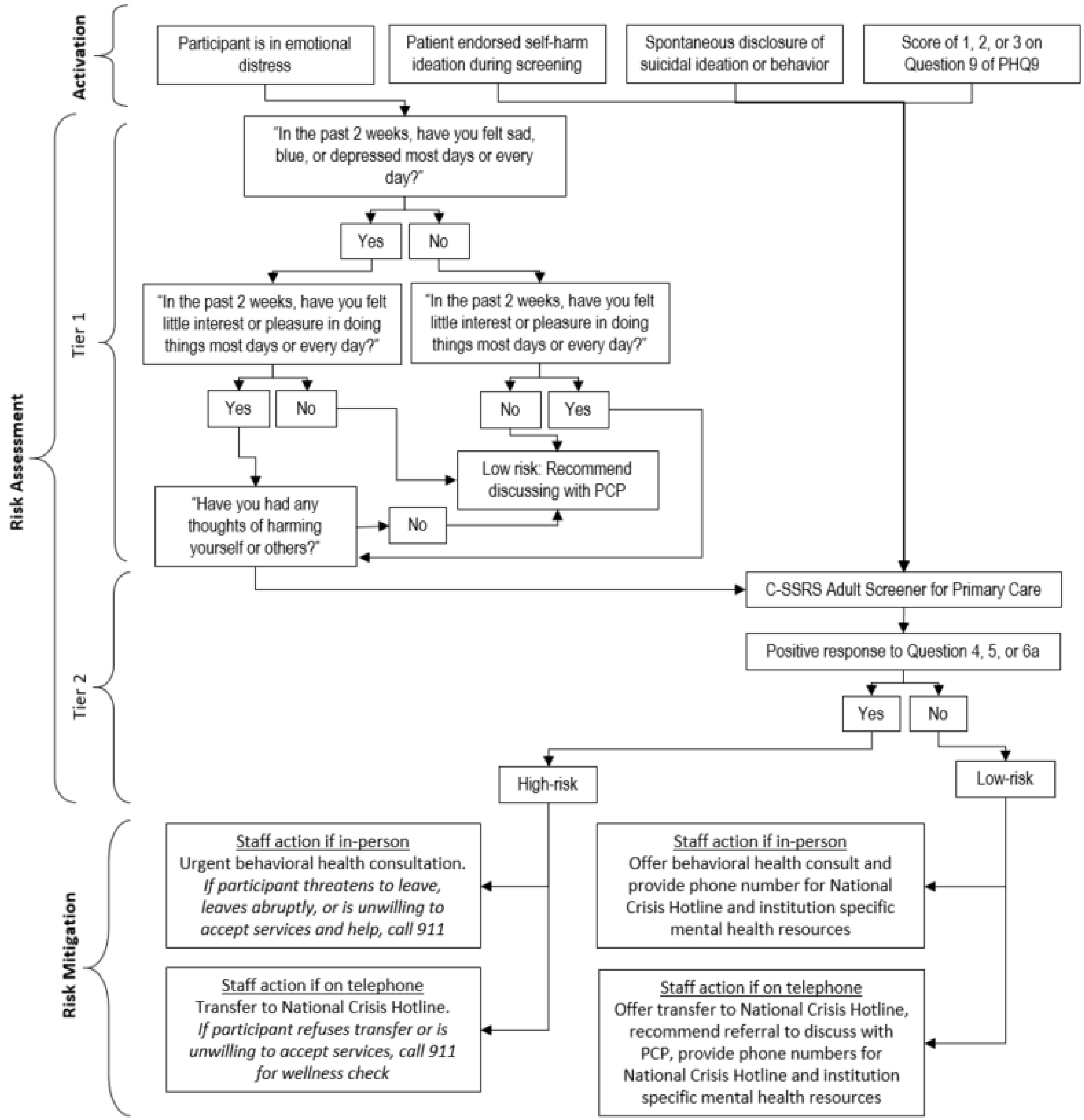
Activation
Participant is in emotional distress
Patient endorsed self-harm ideation during screening
Spontaneous disclosure of suicidal ideation or behavior
Score of 1, 2, or 3 on Question 9 of PHQ9
Risk Assessment
Tier 1
"In the past 2 weeks, have you felt sad, blue, or depressed most days or every day?"
Yes
No
"In the past 2 weeks, have you felt little interest or pleasure in doing things most days or every day?"
"In the past 2 weeks, have you felt little interest or pleasure in doing things most days or every day?"
Yes
No
No
Yes
Low risk: Recommend discussing with PCP
"Have you had any thoughts of harming yourself or others?"
No
Tier 2
C-SSRS Adult Screener for Primary Care
Positive response to Question 4, 5, or 6a
Yes
No
High-risk
Low-risk
Risk Mitigation
Staff action if in-person
Urgent behavioral health consultation.
If participant threatens to leave, leaves abruptly, or is unwilling to accept services and help, call 911
Staff action if on telephone
Transfer to National Crisis Hotline.
If participant refuses transfer or is unwilling to accept services, call 911 for wellness check
Staff action if in-person
Offer behavioral health consult and provide phone number for National Crisis Hotline and institution specific mental health resources
Staff action if on telephone
Offer transfer to National Crisis Hotline, recommend referral to discuss with PCP, provide phone numbers for National Crisis Hotline and institution specific mental health resources

Columbia-Suicide Severity Rating Scale (Adult Version – Screener – Recent)		
Step 1: Risk Assessment		
BEGIN HERE...Ask Questions 1 and 2	Answer	
1 Over the past 30 days, have you wished you were dead or wished you could go to sleep and not wake up?	No	Yes
2 Have you actually had any thoughts of killing yourself?<br>*If YES, ask questions 3, 4, 5 and 6. If NO, go directly to question 6.*	No	Yes
3 Have you been thinking about how you might kill yourself?	No	Yes
4 Have you had these thoughts and had some intention of acting on them?	No	Yes
5 Have you started to work out or worked out the details of how to kill yourself? Do you intend to carry out this plan?	No	Yes
6 Have you ever done anything, started to do anything, or prepared to do anything to end your life?<br>*If YES, ask*: How long ago did you do any of these?	No	Yes

❑ Within past 3 months			No	Yes
❑ Within 3 months to 1 year ago			No	Yes
❑ Over 1 year ago			No	Yes
Step 2: PLUTO Staff Action				
Did participant answer YES to question 4, 5, or 6a?	If you are IN PERSON at UMN…	If you are IN PERSON at the VA…	If you are on the TELEPHONE…	
No	Offer Behavioral Health consult<br>*OR*<br>Refer to PCP or mental health care provider<br>*AND*<br>Give Crisis Hotline # pamphlet	Offer to walk to ED for consult or call Mental Health Intake for consult.<br>*OR*<br>Refer to PCP or mental health care provider<br>*AND*<br>Give Crisis Hotline and Mental Health Intake # cards	Offer transfer to Crisis Hotline #<br>*OR*<br>Refer to PCP or mental health care provider	Non-Imminent Risk
Yes	Page Behavioral Health for consult. Remain with participant until consult arrives. If participant threatens to leave, leaves abruptly, or is unwilling to accept services and help, call 911	Call Mental Health Intake for consult. Remain with participant until consult arrives. If participant threatens to leave, leaves abruptly, or is unwilling to accept services and help, call 911	Offer transfer to Crisis Hotline<br>If REFUSED, call 911 for wellness check	Imminent Risk

**PLUTO Study Suicide Risk Management Protocol Post-activation Follow-up Guidelines**			
**Note, the study coordinator and site PI should be notified following each use of the SRMP*			
**Time point**	**Risk Class**	**Site**	
		UMN/Allina	VA
**Before consent** (e.g., during recruitment or screening)	Low-Risk	1. Message patient's mental health provider and/or PCP with summary of encounter and resources offered/accepted<br>2. Notify patient that they will not be will not be enrolled in study (during screening); end recruitment contact	1. Enter EMR note with summary of encounter and resources offered/accepted, adding patient's mental health provider and/or PCP to the note as a co-signer<br>2. Notify patient that they will not be will not be enrolled in study (during screening); end recruitment contact
	High-Risk	1. Message patient's mental health provider and/or PCP with summary of encounter and resources offered/accepted<br>2. Notify patient that they will not be will not be enrolled in study in follow-up phone call 1-2 days later; end recruitment contact	1. Enter EMR note with summary of encounter and resources offered/accepted, adding patient's mental health provider and/or PCP to the note as a co-signer<br>2. Notify patient that they will not be will not be enrolled in study in follow-up phone call 1-2 days later; end recruitment contact
**After Consent**	Low-Risk	1. Message patient's mental health provider and/or PCP with summary of encounter and resources offered/accepted<br>2. Continue subject's participation as normal	1. Enter EMR note with summary of encounter and resources offered/accepted, adding patient's mental health provider and/or PCP to the note as a co-signer<br>2. Continue subject's participation as normal
	High-Risk	1. Message patient's mental health provider and/or PCP with summary of encounter and resources offered/accepted<br>2. Determine with PI and patient's PCP/mental health provider whether continued participation for the subject is appropriate. If withdrawal is warranted, follow protocol for orderly termination of participation by PI	1. Enter EMR note with summary of encounter and resources offered/accepted, adding patient's mental health provider and/or PCP to the note as a co-signer<br>2. Determine with PI and patient's PCP/mental health provider whether continued participation for the subject is appropriate. If withdrawal is warranted, follow protocol for orderly termination of participation by PI

In the event a participant is hospitalized for mental health reasons or is classified by high-risk by the suicide risk management protocol, the study coordinator and/or PI will communicate with the participant's mental health and/or primary care provider to determine whether continued participation in the study is appropriate.

13.2 **Reproduction Risks:** N/A.

13.3 **Risks to Others:** N/A.

13.4 COVID-19 Risk Mitigation

The majority of study procedures (treatment and data collection) have always been conducted remotely, by telephone. However, in response to the COVID-19 public health

crisis, the following protocol changes were implemented 3/20/2020 to enable the study to continue and minimize risk:

(1) All scheduled in-person MTM study visits will be cancelled and rescheduled as phone consults. All future MTM study encounters will be held by telephone.

(2) All research staff (counselors and data collection research assistants) are working remotely. All phone contacts will be conducted through the Doximity App, a HIPAA compliant app for healthcare professionals that allows the caller to choose the number the participant sees on their caller ID. All research staff will use the PLUTO study # on their outgoing calls (844-389-5619).

(3) Collection of saliva samples will be halted until it is deemed appropriate and safe to continue collection. Verification of tobacco abstinence may be obtained using an iCO™ Smokerlyzer® in place of salivary cotinine (see

14.0 Potential Benefits to Participants

14.1 **Potential Benefits**: Participants may benefit from smoking cessation or smoking reduction.

15.0 Statistical Considerations

15.1 Data Analysis Plan:

15.1.1 Specific Aims

The proposed trial design of adaptive interventions is equivalent to a factorial design.[4-6, 107] Similar to standard methods for adaptive intervention trials conducted in other therapeutic areas, analyses to address the Primary Aim and Secondary Aim 1 will be a test of the main effect of the randomized intervention. The primary (dichotomous) outcome for all analyses will be whether or not smokers achieve 6-month prolonged abstinence measured at 18 months after the baseline assessment. All analyses will be by intention-to-treat, and include all participants randomized to intervention, regardless of whether they are lost to follow-up. Methods to handle non-response of the primary outcome are discussed below.

To test the hypothesis specified for the Primary Aim we will compare, among incomplete responders, the proportion of participants with prolonged abstinence between those randomized to TLC (cells 3 and 7, Figure 5) and TLC+MTM (cells 4 and 8) using a two-sided Wald-type test[108] of the difference in proportions with a significance level of 95%. To test the hypothesis specified for Secondary Aim 1, we will conduct a Wald-type superiority test to compare the proportion of participants with prolonged abstinence among complete responders between those randomized to TLC (cells 2 and 6) and TLC-Q (cells 1 and 5). To test the effect of the proposed moderators (Secondary Aim 2), we will fit a logistic regression model within the relevant subpopulation which includes a main effect for the randomized treatment group, the moderator, and moderator by treatment interactions. Wald-type tests will be used to assess if the interaction term is significant (at

the 90% level for this aim) indicating that the covariate significantly moderates the proposed treatment effect. For example, to address Hypothesis 3a, we will fit a logistic regression model among incomplete responders to first line treatment with covariates for second-line treatment (TLC+MTM versus TLC), number of days smoking following the quit date, and their interaction. To evaluate Hypothesis 3b, we will fit a logistic regression model among complete responders to first line treatment with covariates for second-line treatment (TLC versus TLC-Q), assessment time for incomplete response (4 vs. 8 weeks), and their interaction. We will use the Lung-RADS score as an ordinal measure of lung cancer screening findings for the Exploratory Aims. A Cochran-Armitage trend test108 will evaluate if the proportion of smokers achieving long-term abstinence differs by the degree of screening abnormalities. Similarly, the Jonckheere-Terpstra trend test,[109] a generalization of the Kruskil-Wallis test for ordinal predictors, will assess if progress toward quitting differs by Lung-RADS score.

15.1.2 Exploratory Analyses

We will supplement these unadjusted analyses of the effect of various interventions on the primary outcome with multiple logistic regression which includes demographics and other baseline factors included in Table 2. These factors will be selected using results from previous studies and based on expert clinical opinion, including age, gender, marital status, income category, quit attempt in previous year, cigarettes per day at baseline, time to first cigarette in the day at baseline, withdrawal symptoms, and psychiatric co-morbidity. The t-test, supplemented by multiple regression, will be used to compare the interventions as described above but with other secondary, continuous endpoints including smoking reduction from baseline, quit attempts, and treatment satisfaction (see Table 2). Because many variables will be assessed repeatedly (e.g. interest in quitting, self-efficacy, withdrawal, smoking reduction) the same models (with 1] only the treatment group and 2] adjusted for the factors above) will be fit with the longitudinal data using generalized estimating equations to account for the correlation between repeated measures. To assess the role of the treatment group on potential mediators of long-term abstinence including self-efficacy, stress, interest in quitting, satisfaction, and how progress to quitting moderates the mediation, we will undertake a mediated moderation analysis.[110-112] Importantly, the data collected as part of this trial will permit us to estimate the proportion of participants achieving prolonged abstinence within each of the 8 embedded adaptive interventions and allow us to explore how to tailor the interventions based on an individual's characteristics through Q- and A-learning.[113-126]

15.2 **Power Analysis:** The proposed trial is powered for a main effects analysis of the effect of TLC compared to TLC+MTM among the incomplete responders, where the outcome is 6-month prolonged abstinence measured at 18 months after baseline. We emphasize that the power calculation for the **Primary Aim** is equivalent to a standard two-arm intervention trial to be conducted among a population of incomplete responders to first-line treatment.

Power for the Primary Aim is shown in **Table 3** assuming a total sample size of 1,000 Participants under different assumptions about the proportion of complete and incomplete responders to first-line treatment, and the proportion of long-term abstinence in TLC vs. TLC+MTM. We considered a difference between treatment groups of 10% to be of interest. The hypothesized proportion of incomplete responders (65-75%) averaged across the two assessment time points is based on the 21-day and 3-month abstinence rates in the TLC trial which were 58% and 68% respectively. The hypothesized proportion of incomplete responders achieving prolonged abstinence among those randomized to TLC (10-20%) is similarly based on data from the TLC trial (20% and 19% of participants who were smoking at 21 days and 3 months, respectively, had prolonged abstinence at 18 months). **Table 3** indicates we have sufficient power to detect a 10% difference and even reasonable power across many scenarios to detect a 7.5% difference.

Table 3. Power to test a difference in the proportion of Participants achieving prolonged abstinence among incomplete responders for TLC vs. TLC+MTM (Primary Aim). Power calculations assume total N=1,000 and the use of a two-sided test with a 95% significance level.

Proportion incomplete responders=75%				Proportion incomplete responders=65%			
	TLC responders				TLC responders		
**TLC+MTM responders**	**10%**	**15%**	**20%**	**TLC+MTM responders**	**10%**	**15%**	**20%**
**7.5% diff**	85%	75%	68%	**7.5% diff**	79%	68%	61%
**10% diff**	97%	93%	89%	**10% diff**	95%	89%	84%

Table 4. Power for the superiority analysis of TLC vs. TLC-Q among complete responders (Secondary Aim 2). Total N=1,000, proportion of incomplete responders assumed to be 65%; 90% significance level.

	TLC responders		
**TLC-Q responders**	**20%**	**30%**	**40%**
**7.5% diff**	73%	62%	57%
**10% diff**	91%	81%	75%

We also show sufficient power to test our hypotheses for **Secondary Aim 1 (Table 4)**. For the superiority analysis of TLC versus TLC-Q among complete responders, we provide power calculations under the assumption that the proportion of early incomplete response is 35%. In the TLC trial, the proportion of participants that were complete responders (measured at 21 days) who achieved prolonged abstinence was 40%. To assess the power of whether degree of treatment response moderates the effect of TLC+MTM among the incomplete responders (**Secondary Aim 2**, interaction of amount of smoking following the quit date with TLC/TLC+MTM), we assume that 65% of initial sample will be incomplete responders and that the percentage of incomplete responders randomized to TLC and TLC+MTM with prolonged abstinence is 20% and 30%, respectively, averaged across different amounts of treatment response. For ease of power estimates, we dichotomized amount of treatment response into two groups with ~50% of the population in each group. If TLC+MTM has no effect among those with fewer days of smoking and the group of incomplete responders with fewer days of smoking has a prolonged abstinence rate between 10% and 20%, we will have 62%-83% power to detect a significant interaction effect between second-line treatment and amount of response assuming a two-sided Wald-type test using a 90% significance level. *Treating number of days smoking following the quit date as a continuous covariate would increase the power to detect a significant interaction.* Similarly, to test the assessment time (**Secondary Aim 2**, 4 vs. 8 weeks) as a moderator for second-line treatment among complete responders, we make assumptions about the effect

of TLC and TLC-Q on long-term abstinence averaged across the two assessment times identical to those described for Secondary Aim 1. If, among *complete responders*, we assume no difference in prolonged abstinence assessed at 8 weeks between TLC and TLC-Q and higher prolonged abstinence for those assessed at 4 weeks among those randomized to TLC, we have between 66% and 90% power to detect a significant interaction effect between second-line treatment and assessment time using a two-sided Wald-type test and a 90% significance level.

15.3 **Statistical Analysis:**

15.3.1 Analytic Methods to Address Missing Data

We will address missing response data using a variety of sensitivity analyses. As a preliminary analysis, we consider non-responders to be continuing smokers. However, this approach is not necessarily a conservative assumption.[127] We will use multiple imputation techniques[128-130] for missing outcomes including the primary outcome. We anticipate that nonresponse will depend on the unobserved smoking status of the Participant; that is, we assume a not missing at random (NMAR) missingness mechanism. Therefore, similar to the analysis proposed in Fu et al.,[64] we will construct a joint longitudinal mixed-effects model of smoking status (abstinent versus non-abstinent) and response status measured at various times during the follow-up for our imputation model. (Similar joint longitudinal models for other outcomes can be constructed for secondary analyses). Following imputation, standard statistical methods can be applied accounting for the multiply imputed outcomes (SAS Proc MIANALYZE). We will consider any assessment of 18-month abstinence that is not cotinine verified to be missing (and impute the missing value as described above). Therefore, all randomized Participants will be included in analyses, and over-recruitment for loss to follow-up will not be necessary.

15.4 **Data Integrity:** Data will be stored in REDCap, which is a secure, web-based application designed to support data capture for research studies. Access to REDCap projects for this study will be restricted by project role to study team members. Ability to delete, rename, or export records will be limited to the study coordinator and statistician. Audit trails will be utilized to track data manipulation and exports. Fields will be flagged as required and data quality checks for missing data will be executed.

16.0 Confidentiality

16.1 **Data Security:**

Data for this study will be entered into a REDCap database, which uses a MySQL database via a secure web interface with data checks used during data entry to ensure data quality. REDCap includes a complete suite of features to support HIPAA compliance, including a full audit trail, user-based privileges, and integration with the institutional LDAP server. The MySQL database and the web server are both housed on secure servers operated by the University of Minnesota Academic Health Center's Information Systems group (AHC-IS).

The servers are in a physically secure location on campus and are backed up nightly, with the backups stored in accordance with the AHC-IS retention schedule of daily, weekly, and monthly tapes retained for 1 month, 3 months, and 6 months, respectively. Weekly backup tapes are stored offsite.

Access to the study's data in REDCap is restricted to the members of the study team by username and password. Research data is collected from the participant and entered into the database only after written informed consent and HIPAA authorization granting disclosure of PHI are obtained. Data sources for this research include electronic surveys (administered verbally by data collectors in-person or over telephone and recorded in REDCap), tobacco cessation counseling notes (recorded in REDCap) and CPRS. All data collectors and tobacco cessation counselors at the University of Minnesota (coordinating site) have VA WOC privileges with the Minneapolis VAMC. All research staff will have completed the appropriate CITI and HIPAA training prior to working with participant data.

In the rare event that REDCap is inaccessible to our study team due to an unforeseen circumstance (e.g. network outage, emergency maintenance to REDCap server), data may be collected on paper data collection forms and later be entered into REDCap once the database is available. Once all data is entered into REDCap from a paper data collection form, the form would be placed into a designated PHI bin for shredding.

A copy of the consent form will not be placed in the patient’s electronic medical record. Per Fairview Research Administration policy, participants will be enrolled in OnCore which will automatically apply a flag in the patient’s electronic medical record. This flag will include a description of the study and contact information for the PI and study coordinator. This flag is removed following completion of the study.

Identifiers are specifically flagged in REDCap. Data exported from REDCap will be compliant with the University’s Privacy Office guidance on securing and de-identification of data.

Record Retention

The investigator will retain study records including source data, copies of case report form, consent forms, HIPAA authorizations, and all study correspondence in a secured facility for at 6 years after the study file is closed with the IRB and FDA.

17.0 Provisions to Monitor the Data to Ensure the Safety of Participants

This is a laboratory study that is considered low risk.

The study’s Data and Safety Monitoring Plan will be in compliance with the University of Minnesota Masonic Cancer Center’s Data & Safety Monitoring Plan (DSMP), which can be found at:
https://www.cancer.umn.edu/sites/cancer.umn.edu/files/cprc_data_safety_monitoring_policy_

04.18.2017.pdf. Per the DSMP, low risks studies do not require monitoring by the Masonic Cancer Center.

At the time of IRB annual review, a copy of the submission will be provided to the CPRC unless otherwise waived.

17.1 **Data Integrity Monitoring.** N/A

17.2 **Data Safety Monitoring.**

The principal investigator and study team will assume responsibility for monitoring (1) enrollment, avoiding unnecessary exposure to risks; (2) adverse events; (3) data completeness; (4) outcome data; (5) protocol non-compliance; and (6) new and relevant information. Additional oversight will be provided by a Data and Safety Monitoring Board (DSMB). The DSMB will be responsible for safeguarding the interests of trial participants, assessing safety, and monitoring the overall conduct of the clinical trial. The DSMB Charter, which defines the responsibilities of the DSMB, its membership, and the timing of meetings, is available as a Supporting Document.

18.0 Provisions to Protect the Privacy Interests of Participants

18.1 **Protecting Privacy**: Call protocols for the recruitment phase of this study use a privacy-sensitive protocol (e.g., we will not leave voicemails with information about the nature of the study). The majority of study contacts occur telephonically. We will ask for permission to leave voicemails on the participant's phone following consent. Research staff who will facilitate phone calls are located in a private office suite that is access-restricted by key. Within the study team, there are separate personnel for data collection and telephone counseling; minimal data will be shared between the two personnel groups. Data and tobacco counseling notes will be entered into REDCap, which uses a MySQL database via a secure web browser. Minimally necessary access within REDCap is granted to research staff to protect participant privacy.

Biologic samples (saliva samples) – Subjects who are selected to provide a saliva sample will be mailed a testing kit, along with instructions and a postage-paid box for return to the coordinating center at the University of Minnesota. The kit will include their study ID number only. They will be instructed not to include their name and address on the envelope. The sample will only be identified by the subject's study ID number. All specimens will be destroyed within 3 months after the tests have been completed

18.2 **Access to Participants***:* Participants are counseled during the consent process regarding the nature of the medical records the research team will access. Access to medical records is necessitated by this study's exploratory aim.

19.0 Compensation for Research-Related Injury

19.1 Compensation for Research-Related Injury: N/A

19.2 Contract Language: N/A

20.0 Consent Process

20.1 **Consent Process (when consent will be obtained)**: All potential study participants will be given a copy of the IRB-approved consent to review. The investigator or designee will explain all aspects of the study in lay language and answer all questions regarding the study. If the participant decides to participate in the study, he/she will be asked to sign and date the consent document. Patients who refuse to participate or who withdraw from the study will be treated without prejudice.

When possible, informed consent will be obtained in person. Informed consent may be obtained by telephone in the event the study team cannot feasibly obtain informed consent in person. Written informed consent and HIPAA authorization will be obtained from all subjects. The study team will provide a short description of the study to all potential subjects over the phone (scripting can be found in Appendix G). Interested individuals will be screened for study eligibility. For eligible potential subjects whom informed consent and HIPAA authorization cannot be feasibly obtained in person, the study team will mail a blank consent form and HIPAA Authorization Form, along with a cover letter introducing the study. A stamped, self addressed envelope will be provided with the packet so the subject can mail back the consent and HIPAA Authorization Form. Once the potential subject receives the packet, they are instructed by the cover letter to call the study team. During this phone conversation, a member of the study team will walk through the entire consent and HIPAA Authorization Form (scripting can be found in Appendix H). Once all questions are answered over the phone, the subject will be instructed to sign the consent and HIPAA Authorization forms if they are willing to participate in the study. The subject then will mail the consent and HIPAA Authorization forms back to the study team. No study activities will begin until the signed consent and HIPAA authorization forms are received. The participant will be mailed signed copies of the consent and HIPAA forms.

In the event an illiterate individual is interested in study participation and is determined to be eligible by the eligibility screen, a witness to the consent process will be required. The witness will not be a member of the research team and will be present during the informed consent process (if the telephone consent process is used, the witness will be asked to join the telephone call by speaker phone). In addition to signatures from the subject and person obtaining consent, the witness to the consent process will be asked to provide a signature.

20.2 **Waiver or Alteration of Consent Process (when consent will not be obtained)**: N/A.

20.3 **Non-English Speaking Participants:** This study will only enroll English speaking participants.

20.4 **Participants Who Are Not Yet Adults (infants, children, teenagers under 18 years of age):**

N/A.

20.5 **Cognitively Impaired Adults, or adults with fluctuating or diminished capacity to consent:** N/A

20.6 **Adults Unable to Consent:** N/A

21.0 Setting

21.1 **Research Sites:** This study is primarily telephonic. Recruitment, enrollment, and baseline visits may occur in-person at the M Health Clinics and Surgery Center or University of

Minnesota Medical Center (at the preference of the patient). Patients may be recruited from the University of Minnesota Medical Center, MHealth clinics, and Fairview Community Clinics. The recruitment procedures for this study comply with Fairview Research Administration guidelines.

21.2 **International Research**: N/A

22.0 Multi-Site Research

22.1 **Study-Wide Number of Participants:** 1000 enrolled at the University of Minnesota, Allina Health, and Minneapolis VA Health System

22.2 **Study-Wide Recruitment Methods:** Recruitment will be coordinated with the lung cancer screening programs at each respective site. Recruitment plans have been tailored and approved by each site's IRB.

22.3 **Study-Wide Recruitment Materials:** N/A

22.4 **Communication Among Sites:** In this multi-site study, the University of Minnesota is the "Coordinating Site." Allina Health and the Minneapolis VA have independent IRB approval; the Coordinating Site does not serve as the IRB of record. In addition to regular meetings between the research team and Coordinating Site, additional standards will be met:

- All sites will have the most current version of the protocol (provided by the Coordinating Site), consent document(s), and HIPAA authorization;
- All required approvals (initial, continuing review, and modifications) have been obtained at each site and records will be maintained at the Coordinating Site;
- All modifications will be communicated to all sites and approval will be obtained at all sites prior to implementation of the modification;
- All engaged participating sites will safeguard data, including secure transmission of data, as required by local information security policies;
- All local site investigators conduct the study in accordance with applicable federal regulations and local laws;
- All non-compliance with the study protocol or applicable requirements will be reported in accordance with university or local policy;
- All other reportable events will be reported in accordance with university or local policy.
- Adverse events occurring at other study sites will be reported to the Coordinating Site PI (Dr. Joseph) or her designee

22.5 **Communication to Sites**: The Coordinating Site is responsible for scheduling recurring meetings to facilitate ongoing monitoring of 1) enrollment; (2) adverse events; (3) data completeness; (4) outcome data; (5) protocol non-compliance; and (6) new and relevant information.

22.6 **SCALE Collaboration:** This trial is part of the National Cancer Institute-sponsored SCALE (Smoking Cessation at Lung Examination) collaboration. The purpose of the collaboration is to share de-identified data and methods from eight funded research projects to enable cross-project research on smoking cessation interventions in the setting of lung cancer

screening. De-identified data will be sent to the National Cancer Institute using their secure portal. Data collected from participants enrolled in the trial as well as data collected about patients who are eligible to enroll but ultimately decline to participate will be sent to NCI. Data will be de-identified in accordance with UMN policy and the HIPAA de-identification standard (https://www.healthprivacy.umn.edu/policies-procedures/creating-de-identified-data-set). All data will be transferred as described by a Data Transfer Agreement executed between the University of Minnesota and the National Cancer Institute.

23.0 Resources Available

23.1 Resources Available:

Facilities & Equipment

This study has a suite of offices in the Mayo Building at the University of Minnesota. Staff use IBM-type personal computers supported by the AHC-IS. Laboratory space for Dr. Sharon Murphy is available for the processing of salivary cotinine samples at the Cancer and Cardiovascular Research Building at the University of Minnesota.

Recruitment

Table 1. Patients available for recruitment, target N=1,000 (rounded to the nearest 10)

	UMN	VAMC	Total
# Current smokers/year age 55-79	3860*	~3500**	7360
# Smokers responding to lung cancer screening invitation/year	1540***	500****	2040
# Eligible to participate in study/year (estimate 70%† of smokers screened)	1080	350	1430
# Enroll in study/year (estimate 60% of eligible)	650	210	860
# Potential enrollees/total recruitment period‡	1630	470	2100

*Based on ICD9 code of tobacco use disorder or EMR designation of SOC_HX of being a smoker
**Based on CPRS, national data, an estimate of the # of smokers in current VA research sample
***Based on 40% order completion rate at UMN
****Based on CT scan capacity, potential to preferentially invite current smokers, and 15% response to invite in VA lung cancer screening program
†Based on interest in quitting smoking in the lung cancer screening literature, VA survey data
‡Total recruitment period UMN 2.5 years, VAMC 2.25 years (staggered recruitment start)

Other Resources

The study team will meet on a monthly basis to ensure that all persons assisting with the research are adequately informed about the protocol, the research procedures, and their duties and functions.

Mental health professionals are available at study sites locally to assist research staff who need consultation for participants requiring mental health evaluation per the study suicide risk management protocol. Drs. Joseph (UMN), Begnaud (UMN), Fu (VAMC), Melzer (VAMC), and Lee (Allina) are available to the study team for assessing eligibility (e.g., reviewing recent mental health medication changes), determining the severity of an adverse event, or in any other event that a medically-qualified personnel may be needed for study related activities.

24.0 References

1. Hughes JR, Keely J, Naud S. Shape of the relapse curve and long-term abstinence among untreated smokers. Addiction 2004;99(1):29-38.

2. Fiore MC JC, Baker TB, et al. Treating Tobacco Use and Dependence: 2008 Update. Clinical Practice Guideline. Rockville, MD: U.S. Department of Health and Human Services. Public Health Service; 2008.

3. Coleman K, Austin BT, Brach C, Wagner EH. Evidence on the Chronic Care Model in the new millennium. Health Aff (Millwood) 2009;28(1):75-85.

4. Collins LM, Murphy SA, Bierman KL. A conceptual framework for adaptive preventive interventions. Prev Sci 2004;5(3):185-96. PMCID: PMC3544191.

5. Lavori PW, Dawson R. Dynamic treatment regimes: practical design considerations. Clin Trials 2004;1(1):9-20.

6. Murphy SA. An experimental design for the development of adaptive treatment strategies. Stat Med 2005;24(10):1455-81.

7. Nahum-Shani I, Qian M, Almirall D, Pelham WE, Gnagy B, Fabiano GA, et al. Experimental design and primary data analysis methods for comparing adaptive interventions. Psychol Methods 2012;17(4):457-77. PMCID: PMC3825557.

8. The Methodology Center. Projects using SMART. Available at http://methodology.psu.edu/ra/adap-treat-strat/projects. Accessibility verified September 2014.

9. Hall SM, Humfleet GL, Munoz RF, Reus VI, Robbins JA, Prochaska JJ. Extended treatment of older cigarette smokers. Addiction 2009;104(6):1043-52.

10. Ellerbeck EF, Mahnken JD, Cupertino AP, Cox LS, Greiner KA, Mussulman LM, et al. Effect of varying levels of disease management on smoking cessation: a randomized trial. Ann Intern Med 2009;150(7):437-46.

11. Joseph AM, Fu SS, Lindgren B, Rothman AJ, Kodl M, Lando H, et al. Chronic disease management for tobacco dependence: a randomized, controlled trial. Arch Intern Med 2011;171(21):1894-900.

12. Sinclair HK, Bond CM, Stead LF. Community pharmacy personnel interventions for smoking cessation. Cochrane Database Syst Rev 2004(1):CD003698.

13. West R, McNeill A, Raw M. Smoking cessation guidelines for health professionals: an update. Health Education Authority. Thorax 2000;55(12):987-99. PMCID: PMC1745657.

14. Maguire TA, McElnay JC, Drummond A. A randomized controlled trial of a smoking cessation intervention based in community pharmacies. Addiction 2001;96(2):325-31.

15. DaVanzo J, Dobson A, Koenig L, Book R. Medication Therapy Management Services: A Critical Review. 2005.

16. American Pharmacists A, National Association of Chain Drug Stores F. Medication therapy management in pharmacy practice: core elements of an MTM service model (version 2.0). J Am Pharm Assoc (2003) 2008;48(3):341-53.

17. McDonough RP, Doucette WR. Drug therapy management: an empirical report of drug therapy problems, pharmacists' interventions, and results of pharmacists' actions. J Am Pharm Assoc (2003) 2003;43(4):511-8.

18. Moczygemba LR, Barner JC, Lawson KA, Brown CM, Gabrillo ER, Godley P, et al. Impact of telephone medication therapy management on medication and health-related problems, medication adherence, and Medicare Part D drug costs: a 6-month follow up. Am J Geriatr Pharmacother 2011;9(5):328-38.

19. Branham AR, Katz AJ, Moose JS, Ferreri SP, Farley JF, Marciniak MW. Retrospective analysis of estimated cost avoidance following pharmacist-provided medication therapy management services. J Pharm Pract 2013;26(4):420-7.

20. Machado M, Bajcar J, Guzzo GC, Einarson TR. Sensitivity of patient outcomes to pharmacist interventions. Part I: systematic review and meta-analysis in diabetes management. Ann Pharmacother 2007;41(10):1569-82.

21. National Lung Screening Trial Research T, Aberle DR, Adams AM, Berg CD, Black WC, Clapp JD, et al. Reduced lung-cancer mortality with low-dose computed tomographic screening. N Engl J Med 2011;365(5):395-409.

22. Humphrey LL, Deffebach M, Pappas M, Baumann C, Artis K, Mitchell JP, et al. Screening for lung cancer with low-dose computed tomography: a systematic review to update the US Preventive services task force recommendation. Ann Intern Med 2013;159(6):411-20.

23. Bach PB, Mirkin JN, Oliver TK, Azzoli CG, Berry DA, Brawley OW, et al. Benefits and harms of CT screening for lung cancer: a systematic review. JAMA 2012;307(22):2418-29. PMCID: PMC3709596.

24. Wender R, Fontham ETH, Barrera E, Colditz GA, Church TR, Ettinger DS, et al. American Cancer Society lung cancer screening guidelines. CA: A Cancer Journal for Clinicians 2013;63(2):106-117.

25. Wood DE, Eapen GA, Ettinger DS, Hou L, Jackman D, Kazerooni E, et al. Lung cancer screening. J Natl Compr Canc Netw 2012;10(2):240-65.

26. Detterbeck FC, Mazzone PJ, Naidich DP, Bach PB. Screening for lung cancer: Diagnosis and management of lung cancer, 3rd ed: American College of Chest Physicians evidence-based clinical practice guidelines. Chest 2013;143(5 Suppl):e78S-92S. PMCID: PMC3749713.

27. American Society of Clinical Oncology. The role of CT screening for lung cancer in clinical practice. The evidence based practice guideline of the American College of Chest Physicians and the American Society for Clinical Oncology. http://www.asco.org/quality-guidelines/role-ct-screening-lung-cancer-clinical-practice-evidence-based-practice-guideline. 2012.

28. American Lung Association. Providing Guidance on Lung Cancer Screening To Patients and Physicians. Report on Lung Cancer Screening, 2012.

29. U.S. Preventive Services Task Force. Screening for Lung Cancer: Current Recommendation, 2013. http://www.uspreventiveservicestaskforce.org/uspstf/uspslung.htm.2014.

30. Jha P, Ramasundarahettige C, Landsman V, Rostron B, Thun M, Anderson RN, et al. 21st-century hazards of smoking and benefits of cessation in the United States. N Engl J Med 2013;368(4):341-50.

31. Villanti AC, Jiang Y, Abrams DB, Pyenson BS. A cost-utility analysis of lung cancer screening and the additional benefits of incorporating smoking cessation interventions. PLoS One 2013;8(8):e71379. PMCID: PMC3737088.

32. Deppen SA, Grogan EL, Aldrich MC, Massion PP. Lung cancer screening and smoking cessation: a teachable moment? J Natl Cancer Inst 2014;106(6):dju122.

33. Taylor KL, Cox LS, Zincke N, Mehta L, McGuire C, Gelmann E. Lung cancer screening as a teachable moment for smoking cessation. Lung Cancer 2007;56(1):125-34.

34. Hahn EJ, Rayens MK, Hopenhayn C, Christian WJ. Perceived risk and interest in screening for lung cancer among current and former smokers. Res Nurs Health 2006;29(4):359-70.

35. van der Aalst CM, van den Bergh KA, Willemsen MC, de Koning HJ, van Klaveren RJ. Lung cancer screening and smoking abstinence: 2 year follow-up data from the Dutch-Belgian randomised controlled lung cancer screening trial. Thorax 2010;65(7):600-5.

36. Ostroff JS, Buckshee N, Mancuso CA, Yankelevitz DF, Henschke CI. Smoking cessation following CT screening for early detection of lung cancer. Prev Med 2001;33(6):613-21.

37. Schnoll RA, Bradley P, Miller SM, Unger M, Babb J, Cornfeld M. Psychological issues related to the use of spiral CT for lung cancer early detection. Lung Cancer 2003;39(3):315-25.

38. Park ER, Ostroff JS, Rakowski W, Gareen IF, Diefenbach MA, Feibelmann S, et al. Risk perceptions among participants undergoing lung cancer screening: baseline results from the National Lung Screening Trial. Ann Behav Med 2009;37(3):268-79. PMCID: PMC2831282.

39. Krebs P, Li Y, Burkhalter JE, Ostroff JS. Impact of Cancer Diagnosis on Patient and Family Smoking Abstinence. In: Annals of Behavioral Medicine: Springer 233 Spring St., New York, NY 10013 USA; 2011. p. S75-S75.

40. Anderson CM, Yip R, Henschke CI, Yankelevitz DF, Ostroff JS, Burns DM. Smoking cessation and relapse during a lung cancer screening program. Cancer Epidemiol Biomarkers Prev 2009;18(12):3476-83.

41. Townsend CO, Clark MM, Jett JR, Patten CA, Schroeder DR, Nirelli LM, et al. Relation between smoking cessation and receiving results from three annual spiral chest computed tomography scans for lung carcinoma screening. Cancer 2005;103(10):2154-62.

42. Ashraf H, Tonnesen P, Holst Pedersen J, Dirksen A, Thorsen H, Dossing M. Effect of CT screening on smoking habits at 1-year follow-up in the Danish Lung Cancer Screening Trial (DLCST). Thorax 2009;64(5):388-92.

43. Ferketich AK, Otterson GA, King M, Hall N, Browning KK, Wewers ME. A pilot test of a combined tobacco dependence treatment and lung cancer screening program. Lung Cancer 2012;76(2):211-5.

44. Tammemagi MC, Berg CD, Riley TL, Cunningham CR, Taylor KL. Impact of lung cancer screening results on smoking cessation. J Natl Cancer Inst 2014;106(6):dju084. PMCID: PMC4081623.

45. Cox LS, Clark MM, Jett JR, Patten CA, Schroeder DR, Nirelli LM, et al. Change in smoking status after spiral chest computed tomography scan screening. Cancer 2003;98(11):2495-501.

46. Ostroff JS, Yip R, Henkel C, Weiss E, Henschke CI. Preliminary Results Comparing Two Brief Cessation Interventions for Older Smokers Seeking Low-Dose CT Scan of the Chest. Society for Research on Nicotine and Tobacco National Meeting. Houston, Texas; 2012.

47. Styn MA, Land SR, Perkins KA, Wilson DO, Romkes M, Weissfeld JL. Smoking behavior 1 year after computed tomography screening for lung cancer: Effect of physician referral for abnormal CT findings. Cancer Epidemiol Biomarkers Prev 2009;18(12):3484-9. PMCID: PMC2789354.

48. Clark MM, Cox LS, Jett JR, Patten CA, Schroeder DR, Nirelli LM, et al. Effectiveness of smoking cessation self-help materials in a lung cancer screening population. Lung Cancer 2004;44(1):13-21.

49. Wagner EH. The role of patient care teams in chronic disease management. BMJ 2000;320(7234):569-72. PMCID: PMC1117605.

50. Bower P, Gilbody S. Stepped care in psychological therapies: access, effectiveness and efficiency. Narrative literature review. Br J Psychiatry 2005;186:11-7.

51. Sobell MB, Sobell LC. Stepped care as a heuristic approach to the treatment of alcohol problems. J Consult Clin Psychol 2000;68(4):573-9.

52. Abrams DB, Orleans CT, Niaura RS, Goldstein MG, Prochaska JO, Velicer W. Integrating individual and public health perspectives for treatment of tobacco dependence under managed health care: a combined stepped-care and matching model. Ann Behav Med 1996;18(4):290-304.

53. Smith SS, Jorenby DE, Fiore MC, Anderson JE, Mielke MM, Beach KE, et al. Strike while the iron is hot: can stepped-care treatments resurrect relapsing smokers? J Consult Clin Psychol 2001;69(3):429-39.

54. Jaehne A, Loessl B, Frick K, Berner M, Hulse G, Balmford J. The efficacy of stepped care models involving psychosocial treatment of alcohol use disorders and nicotine dependence: a systematic review of the literature. Curr Drug Abuse Rev 2012;5(1):41-51.

55. Bischof G, Grothues JM, Reinhardt S, Meyer C, John U, Rumpf HJ. Evaluation of a telephone-based stepped care intervention for alcohol-related disorders: a randomized controlled trial. Drug Alcohol Depend 2008;93(3):244-51.

56. Stead LF, Perera R, Lancaster T. Telephone counselling for smoking cessation. Cochrane Database Syst Rev 2006(3):CD002850.

57. Hughes JR, Hatsukami D. Signs and symptoms of tobacco withdrawal. Arch Gen Psychiatry 1986;43(3):289-94.

58. Cox LS, Tiffany ST, Christen AG. Evaluation of the brief questionnaire of smoking urges (QSU-brief) in laboratory and clinical settings. Nicotine Tob Res 2001;3(1):7-16.

59. Joseph AM, Norman SM, Ferry LH, Prochazka AV, Westman EC, Steele BG, et al. The safety of transdermal nicotine as an aid to smoking cessation in patients with cardiac disease. New England Journal of Medicine 1996;335(24):1792-1798.

60. Joseph AM, Nelson DB, Nugent SM, Willenbring ML. Timing of alcohol and smoking cessation (TASC): smoking among substance use patients screened and enrolled in a clinical trial. J Addict Dis 2003;22(4):87-107.

61. Joseph AM, Willenbring ML, Nugent SM, Nelson DB. A randomized trial of concurrent versus delayed smoking intervention for patients in alcohol dependence treatment. J Stud Alcohol 2004;65(6):681-91.

62. Joseph AM, Nelson D, Nugent SM, Willenbring ML. Timing of Alcohol and Smoking Cessation (TASC): Study Design, Screening Results and Baseline Charactersitics. Journal of Addictive Diseases 2003;22(4).

63. Joseph AM, Hecht SS, Murphy SE, Lando H, Carmella SG, Gross M, et al. Smoking reduction fails to improve clinical and biological markers of cardiac disease: a randomized controlled trial. Nicotine Tob Res 2008;10(3):471-81.

64. Fu SS, van Ryn M, Sherman SE, Burgess DJ, Noorbaloochi S, Clothier B, et al. Proactive tobacco treatment and population-level cessation: a pragmatic randomized clinical trial. JAMA Intern Med 2014;174(5):671-7.

65. Fu SS, van Ryn M, Burgess DJ, Nelson D, Clothier B, Thomas JL, et al. Proactive tobacco treatment for low income smokers: study protocol of a randomized controlled trial. BMC Public Health 2014;14:337. PMCID: PMC3995758.

66. Finch EA, Linde JA, Jeffery RW, Rothman AJ, King CM, Levy RL. The effects of outcome expectations and satisfaction on weight loss and maintenance: correlational and experimental analyses--a randomized trial. Health Psychol 2005;24(6):608-16.

67. Baldwin AS, Rothman AJ, Hertel AW, Linde JA, Jeffery RW, Finch EA, et al. Specifying the determinants of the initiation and maintenance of behavior change: An examination of self-efficacy, satisfaction, and smoking cessation. Health Psychology 2006;25(5):626-634.

68. Hertel AW, Finch EA, Kelly KM, King C, Lando H, Linde JA, et al. The impact of expectations and satisfaction on the initiation and maintenance of smoking cessation: an experimental test. Health Psychol 2008;27(3 Suppl):S197-206.

69. Burns, RJ, Rothman, AJ, Fu, SS, Lindgren, B, & Joseph, AM. Longitudinal care for smoking cessation: Increasing satisfaction, self-efficacy, and readiness to quit helps struggling smokers succeed. Paper presented at the annual meeting of the Society for Behavioral Medicine, Philadelphia, PA, 2014.

70. Kinsinger LS, Atkins D, Provenzale D, Anderson C, Petzel R. Implementation of a New Screening Recommendation in Health Care: The VHA's Approach to Lung Cancer Screening. Ann Intern Med 2014.

71. Lillie S, Fu SS. Minneapolis VA Lung Cancer Screening Survey. 2014.

72. McKee BJ, Regis SM, McKee AB, Flacke S, Wald C. Performance of ACR Lung-RADS in a Clinical CT Lung Screening Program. Journal of the American College of Radiology (0).

73. Rhoades HM, Overall JE. The semistructured BPRS interview and rating guide. Psychopharmacol Bull 1988;24(1):101-4.

74. Zhu SH, Stretch V, Balabanis M, Rosbrook B, Sadler G, Pierce JP. Telephone counseling for smoking cessation: effects of single-session and multiple-session interventions. Journal of Consulting & Clinical Psychology 1996;64(1):202-211.

75. Friebely J, Rigotti NA, Chang Y, Hall N, Weiley V, Dempsey J, et al. Parent smoker role conflict and planning to quit smoking: a cross-sectional study. BMC Public Health 2013;13:164. PMCID: PMC3600049.

76. Strecher VJ. Computer-tailored smoking cessation materials: a review and discussion. Patient Education and Counseling 1999;36(2):107-17.

77. Polisena J, Coyle D, Coyle K, McGill S. Home telehealth for chronic disease management: a systematic review and an analysis of economic evaluations. Int J Technol Assess Health Care 2009;25(3):339-49.

78. Prochaska JO, DiClemente CC. Stages and processes of self-change of smoking: toward an integrative model of change. Journal of Consulting & Clinical Psychology 1983;51(3):390-395.

79. DiClemente CC, Prochaska JO, Fairhurst SK, Velicer WF, Velasquez MM, Rossi JS. The process of smoking cessation: an analysis of precontemplation, contemplation, and preparation stages of change. Journal of Consulting & Clinical Psychology 1991;59(2):295-304.

80. Nicotine Dependence Education Program. Tobacco Treatment Specialist Certification. http://mayoresearch.mayo.edu/mayo/research/ndc_education/tts_certification.cfm, 2012.

81. Center for Tobacco Treatment and Research. Training and Certification. University of Massachusetts http://www.umassmed.edu/tobacco/training/.

82. Fu S, van Ryn M, Shernan S, Burgess D, Noorbaloochi S, Clothier B, et al. Proactive tobacco treatment and population-level cessation: a pragmatic randomized controlled trial. In: Society for General Internal Medicine. Denver, CO; 2013.

83. Centers for Disease Control and Prevention, NCHS. National Health Interview Survey. Cited in Healthy People 2010.

84. Charlson M, Szatrowski TP, Peterson J, Gold J. Validation of a combined comorbidity index. Journal of Clinical Epidemiology 1994;47(11):1245-1251.

85. Bush K, Kivlahan DR, McDonell MB, Fihn SD, Bradley KA. The AUDIT alcohol consumption questions (AUDIT-C): an effective brief screening test for problem drinking. Ambulatory Care Quality Improvement Project (ACQUIP). Alcohol Use Disorders Identification Test. Arch Intern Med 1998;158(16):1789-95.

86. Kessler RC, Ustun TB. The World Mental Health (WMH) Survey Initiative Version of the World Health Organization (WHO) Composite International Diagnostic Interview (CIDI). Int J Methods Psychiatr Res 2004;13(2):93-121.

87. Kroenke K, Spitzer RL, Williams JB. The PHQ-9: validity of a brief depression severity measure. J Gen Intern Med 2001;16(9):606-13.

88. Population Assessment of Tobacco and Health Study. https://pathstudyinfo.nih.gov/UI/HomeMobile.aspx.

89. Heatherton TF, Kozlowski LT, Frecker RC, Fagerstrom KO. The Fagerstrom Test for Nicotine Dependence: a revision of the Fagerstrom Tolerance Questionnaire. British Journal of Addiction 1991;86(9):1119-1127.

90. Hughes J, Hatsukami DK. Errors in using tobacco withdrawal scale. Tob Control 1998;7(1):92-3. PMCID: PMC1759641.

91. Toll BA, O'Malley SS, McKee SA, Salovey P, Krishnan-Sarin S. Confirmatory factor analysis of the Minnesota Nicotine Withdrawal Scale. Psychol Addict Behav 2007;21(2):216-25. PMCID: PMC2527730.

92. Cohen S, Kamarck T, Mermelstein R. A global measure of perceived stress. J Health Soc Behav 1983;24(4):385-96.

93. Mermelstein R, Cohen S, Lichtenstein E, Baer JS, Kamarck T. Social support and smoking cessation and maintenance. J Consult Clin Psychol 1986;54(4):447-53.

94. Cohen S, Mermelstein R, Kamarck T, Hoberman H. Measuring the Functional Components of Social Support. In: Sarason I, Sarason B, editors. Social Support: Theory, Research and Applications: Springer Netherlands; 1985. p. 73-94.

95. Biener L, Abrams DB. The Contemplation Ladder: validation of a measure of readiness to consider smoking cessation. Health Psychology 1991;10(5):360-365.

96. Herzog TA, Abrams DB, Emmons KM, Linnan L. Predicting increases in readiness to quit smoking: A prospective analysis using the contemplation ladder. Psychology & Health 2000;15(3):369-381.

97. Downey L, Rosengren DB, Donovan DM. Sources of motivation for abstinence: a replication analysis of the reasons for quitting questionnaire. Addict Behav 2001;26(1):79-89.

98. Timeline Followback Instructions and Materials for Alcohol, Cigarettes, Marijuana and Other Drugs and SCQ and DTCQ Feedback Programs. Toronto, CA: Addiction Research Foundation; 1996.

99. Marteau TM, Aveyard P, Munafo MR, Prevost AT, Hollands GJ, Armstrong D, et al. Effect on adherence to nicotine replacement therapy of informing smokers their dose is determined by their genotype: a randomised controlled trial. PLoS One 2012;7(4):e35249. PMCID: PMC3324463.

100. Quinn VP, Stevens VJ, Hollis JF, Rigotti NA, Solberg LI, Gordon N, et al. Tobacco-cessation services and patient satisfaction in nine nonprofit HMOs. Am J Prev Med 2005;29(2):77-84.

101. May S, McEwen A, Arnoldi H, Bauld L, Ferguson J, Stead M. How to measure client satisfaction with stop smoking services: A pilot project in the UK National Health Service. Journal of Smoking Cessation 2009;2(1):52-58.

102. US Department of Health and Human Services. 2000 BRFSS Summary Prevalence Report. Atlanta, Georgia: U.S. Department of Health and Human Services, Centers for Disease Control and Prevention, National Center for Chronic Disease Prevention and Health Promotion, Division of Adult and Community Health, Behavioral Surveillance Branch; 2001.

103. Hughes JR, Keely JP, Niaura RS, Ossip-Klein DJ, Richmond RL, Swan GE. Measures of abstinence in clinical trials: issues and recommendations. Nicotine Tob Res 2003;5(1):13-25.

104. Hughes JR, Benowitz N, Hatsukami D, Mermelstein RJ, Shiffman S. Clarification of SRNT workgroup guidelines for measures in clinical trials of smoking cessation therapies. Nicotine Tob Res 2004;6(5):863-4.

105. Murphy SE, Villalta P, Ho SW, von Weymarn LB. Analysis of [3',3'-d(2)]-nicotine and [3',3'-d(2)]-cotinine by capillary liquid chromatography-electrospray tandem mass spectrometry. J Chromatogr B Analyt Technol Biomed Life Sci 2007;857(1):1-8. PMCID: PMC2234036.

106. Rigotti NA, Park ER, Regan S, Chang Y, Perry K, Loudin B, et al. Efficacy of telephone counseling for pregnant smokers: a randomized controlled trial. Obstet Gynecol 2006;108(1):83-92. PMCID: PMC16816060.

107. Almirall D, Nahum-Shani, Sherwood N, Murphy S. Introduciton to SMART designs for the development of adaptive interventions: with application to weight loss research. Translational Behavioral Medicine (in press).

108. Agresti A. Categorical Data Analysis (Second Edition): Wiley; 2002.

109. Jonckheere A. A distribution-free k-sample test against ordered alternatives. Biometrika 1954;41:133-145.

110. Hayes A. An introduction to mediation, moderation, and conditional process analysis: A regression-based approach. New York: Guilford Press; 2013.

111. Fairchild AJ, MacKinnon DP. A general model for testing mediation and moderation effects. Prev Sci 2009;10(2):87-99. PMCID: PMC2908713.

112. Valeri L, Vanderweele TJ. Mediation analysis allowing for exposure-mediator interactions and causal interpretation: theoretical assumptions and implementation with SAS and SPSS macros. Psychol Methods 2013;18(2):137-50. PMCID: PMC3659198.

113. Chakraborty B, Murphy S, Strecher V. Inference for non-regular parameters in optimal dynamic treatment regimes. Stat Methods Med Res 2010;19(3):317-43. PMCID: PMC2891316.

114. Henderson R, Ansell P, Alshibani D. Regret-regression for optimal dynamic treatment regimes. Biometrics 2010;66(4):1192-201.

115. Chakraborty B. Dynamic treatment regimes for managing chronic health conditions: a statistical perspective. Am J Public Health 2011;101(1):40-5. PMCID: PMC3000720.

116. Moodie EE, Chakraborty B, Kramer MS. Q-learning for estimating optimal dynamic treatment rules from observational data. Can J Stat 2012;40(4):629-645. PMCID: PMC3551601.

117. Moodie E, Platt R, Kramer M. Estimating response-maximized decision rules with applications to breastfeeding. Journal of the American Statistical Association 2009;104(485):155-165.

118. Moodie EE, Richardson TS, Stephens DA. Demystifying optimal dynamic treatment regimes. Biometrics 2007;63(2):447-55.

119. Murphy SA, Lynch KG, Oslin D, McKay JR, TenHave T. Developing adaptive treatment strategies in substance abuse research. Drug Alcohol Depend 2007;88 Suppl 2:S24-30. PMCID: PMC1922034.

120. Murphy SA, Oslin DW, Rush AJ, Zhu J, McAts. Methodological challenges in constructing effective treatment sequences for chronic psychiatric disorders. Neuropsychopharmacology 2007;32(2):257-62.

121. Nahum-Shani I, Qian M, Almirall D, Pelham WE, Gnagy B, Fabiano GA, et al. Q-learning: a data analysis method for constructing adaptive interventions. Psychol Methods 2012;17(4):478-94. PMCID: PMC3747013.

122. Rosthoj S, Fullwood C, Henderson R, Stewart S. Estimation of optimal dynamic anticoagulation regimes from observational data: a regret-based approach. Stat Med 2006;25(24):4197-215.

123. Thall PF, Millikan RE, Sung HG. Evaluating multiple treatment courses in clinical trials. Stat Med 2000;19(8):1011-28.

124. Thall P, Sung H, Estey E. Selecting therapeutic strategies based on efficacy and death in multicourse clinical trials. Journal of the American Statistical Association 2002;97(457):29-39.

125. Watkins C, Dayan P. Q-learning. Mach Learning 1992;8(3-4):279-292.

126. Zhao Y, Kosorok MR, Zeng D. Reinforcement learning design for cancer clinical trials. Stat Med 2009;28(26):3294-315. PMCID: PMC2767418.

127. Nelson DB, Partin MR, Fu SS, Joseph AM, An LC. Why assigning ongoing tobacco use is not necessarily a conservative approach to handling missing tobacco cessation outcomes. Nicotine Tob Res 2009;11(1):77-83.

128. Rubin D. Inference and missing data. Biometrika 1976;63:581-592.

129. Rubin D. Multiple Imputation for Nonresponse in Surveys. New York: John Wiley & Sons, Inc.; 1987.

130. Schafer J. Analysis of Incomplete Multivariate Data. New York: Chapman and Hall; 1997.

131. Kofman M, Dunton K, Senkewicz MB. Implementation of Tobacco Cessation Coverage Under the Affordable Care Act: Understanding How Private Health Insurance Policies Cover Tobacco Cessation Treatment. Washington, DC: Georgetown University Health Policy Institute; 2012. http://www.tobaccofreekids.org/pressoffice/2012/georgetown/coveragereport.pdf.

132. Saba M, Diep J, Saini B, Dhippayom T. Meta-analysis of the effectiveness of smoking cessation interventions in community pharmacy. J Clin Pharm Ther 2014;39(3):240-7.

133. Kahende JW, Loomis BR, Adhikari B, Marshall L. A review of economic evaluations of tobacco control programs. Int J Environ Res Public Health 2009;6(1):51-68. PMCID: PMC2672319.

134. Ruger JP, Lazar CM. Economic evaluation of pharmaco- and behavioral therapies for smoking cessation: a critical and systematic review of empirical research. Annu Rev Public Health 2012;33:279-305. PMCID: PMC3959760.

135. Lal A, Mihalopoulos C, Wallace A, Vos T. The cost-effectiveness of call-back counselling for smoking cessation. Tob Control 2014;23(5):437-42.

136. Warner KE, Mendez D, Smith DG. The financial implications of coverage of smoking cessation treatment by managed care organizations. Inquiry 2004;41(1):57-69.

137. Cromwell J, Bartosch WJ, Fiore MC, Hasselblad V, Baker T. Cost-effectiveness of the clinical practice recommendations in the AHCPR guideline for smoking cessation. Agency for Health Care Policy and Research [see comments]. JAMA 1997;278(21):1759-1766.

138. Barnett PG, Wong W, Hall S. The cost-effectiveness of a smoking cessation program for out-patients in treatment for depression. Addiction 2008;103(5):834-40.

139. Tran MT, Holdford DA, Kennedy DT, Small RE. Modeling the cost-effectiveness of a smoking-cessation program in a community pharmacy practice. Pharmacotherapy 2002;22(12):1623-31.

25.0 Appendix A. TLC Behavioral Counseling and Medication Management Call Guide

I. Structure and Organization of the Telephone Call Guide

The Call Guide for counseling consists of 2 components:

1. A session-by-session "roadmap" containing:
2. Specific treatment objectives to be accomplished
3. Recommended strategies for executing these objectives
4. Examples illustrating recommended strategies

5. Appendix covering intervention strategies that counselors should be familiar with as informed tobacco cessation interventionists: motivational interviewing, pharmacotherapy overview for smoking cessation, and customer service skills

II. Clinical Strategy for Using the Call Guide

- Before each session, read the objectives for the session and acquaint yourself with supplemental information that may be needed for the session.
- Review the notes on the participant from the previous session(s) to familiarize yourself with the participant's most recent plan, particular triggers, barriers, etc.
- During the session, do not be afraid to refer to the treatment manual. It is available for reference as deemed appropriate by the counselor.
- Focus on accomplishing the specific treatment objectives for each session. Use your discretion in following the recommended strategies for executing these objectives while tailoring the session appropriately to the particular participant. Consider the recommended strategies as just that—recommendations, not a rigid set of rules to be followed in "lock-step" fashion.

III. Tobacco Treatment Forms

This manual and the Tobacco Treatment Forms are intended to be used together. The forms are meant to promote consistency in calls. The forms will also assist in gather some of the data that is needed to record treatment. Counselor notes can be helpful for other counselors when a different counselor, other than the primary counselor of the participant, needs to fill in for another with unfamiliar clients if a counselor is sick, on vacation, etc.

IV. Overview of Treatment

1. Once participants set a quit date (during outreach or follow-up calls) there are 6 counseling phone calls over an eight week period, anchored to the quit date.

   a. Outreach call
   b. Pre-quit call — OR Already Quit Call (a and b)
   c. 1-3-day call
   d. 1-week call
   e. 2-week call

f. 4-week call

g. 8-week call

2. If at any point the client relapses at any point before 8 weeks, they are welcome to repeat sessions. After the participant has set a quit date, the counselor should track relapse prevention and slipping/relapses should they occur while working with a participant. At the end of each 1-3-day, 1 week, 2 week, and 4-week phone call, there are specific definitions and categories to indicate if the participant is responding completely, responding incompletely, or not responding at all.

3. Treatment uses a combination of cognitive behavioral therapy strategies and elements of motivational interviewing which emphasizes collaboration, evocation, empathy, and autonomy.

4. Use of nicotine replacement therapy to help with quitting will be addressed with participants.

5. In general, we want to

    a. Resolve participants' ambivalence toward change and promote participants' motivation to change.

    b. Help participants build self-efficacy and set a set quit date.

    c. Assist participants in the development of safe and effective strategies for quitting smoking.

    d. Empower participants with cognitive and behavioral coping strategies and self-management skills maintain tobacco cessation.

V. Plan for Delivering Counseling Sessions

1. Who should deliver counseling interventions?

    Counseling sessions, using motivational interviewing and behavioral strategies should be delivered by a counselor who has an ongoing, continuous relationship with the participant as their primary "point of contact." Ideally, the counselor who completes the outreach phone call with the participant will be their counselor.

2. What modality should be used for delivering sessions?

    Treatment sessions are to be delivered on an individual (not group) basis. All treatment sessions will be delivered over the phone at the participant's convenience.

3. How will calls be scheduled?

    At the end of the baseline data collection visit, the data collection staff will gather participant preferred days and times for the outreach call. Each participant will be assigned a counselor depending upon the participant's given general availability. During the outreach call, the counselor will also set up the pre-quit call. The assigned counselor will make the pre-quit call

during which a quit date and quit (+3 days) call will be scheduled. At the end of each call, counselors will schedule the upcoming call with their participants.

4. What if the participant doesn't answer the phone at the scheduled time?

   If a participant does not answer a scheduled phone call, the counselor will attempt to call the participant 6 times over the following week. If no answer or response, then the counselor will skip a week and try calling 6 more times on the third week. If at that point there is no answer or response, the counselor will send out a missed call letter. If a participant is within one month of their end date of counseling (4 months after their first call), then we will send an end of coaching note instead of a missed call note. Leave voicemail on 1st, 3rd and 6th attempt. If after the 6th attempt is made and the participant was not able to be reached, or did not call back the counselor will send a letter to their home. These attempts should be recorded the, "missed call protocol form. "*Please see Missed Call Protocol for more information.*

VI. Counselor Role in Prescribing Medication

The counselors' responsibility is to provide information about nicotine replacement therapy (monotherapy or combination therapy) and promote its use. Counselors will discuss medication and check in for any concerns or questions about medication during each counseling call. If the participant requests to use a prescription medication (such as nicotine nasal spray, nicotine inhaler, varenicline or bupropion), the counselor will refer the participant to their primary care provider. Although counselors should be informed about these medications and discuss participant questions and concerns during each counseling call, the participant should understand that for prescription medication they should maintain contact with their health care professionals. *More information on discussing medications with participants is found in the Appendix.*

Screening for NRT Use

Use the below form to screen for any potential contraindications to the use of nicotine replacement. If all the questions are answered NO, then it is safe to go ahead and offer the participant the use of nicotine replacement therapy. If the participant answers YES to the question, delay the start of nicotine replacement therapy until two weeks have passed since the date of the heart-related event which required hospitalization.

**MEDICATION SCREENING FORM**		
**I am going to ask you a few question to see if it is safe for you to use nicotine replacement therapy**		
Have you been hospitalized for a heart-related condition in the past 2 weeks?	YES	NO

VII. Participants' FAQs

- Is coaching available 24/7?

  Our toll-free phone number (1-844-389-5619) is available to call 24 hours a day, 7 days a week. You are welcome to call and leave as long a message as you need to talk about any urges you might be having. A quit coach will call you back the next day (with the exception of

weekends and holidays). This quit coach may be different from the one who normally calls you.

- Why did we pick the call schedule that we did and can it be modified?

  The timing of the calls is based on years of research regarding when smokers who are making a quit attempt tend to need the most support. In other words, the call schedule is based on the likelihood of relapse at any given point after the quit attempt. Our goal is to provide extra support at the times you are likely to need it the most and the frequency of the calls will go down as we get further out from the quit date.

  We can modify it within the windows (see Tables 1 and 2) and can always call for check-ins ("maintenance calls") on a more frequent basis. There is also flexibility with scheduling calls depending on the participant's needs.

- Is this program similar to telephone sponsors in AA or NA?

  Our program does not offer quit coaches who are available whenever you have an urge to smoke. We encourage you to talk with a close friend, family member, neighbor, or co-worker who is supportive of your effort to quit smoking about being available to talk to when you have the urge to smoke.

- Are medications I use to help stop smoking free with this study?

  Nicotine replacement therapy is available for free from the study. If you choose to use prescription medications at any point these may be covered by your health insurance, but you will be responsible for any co-pays or costs that insurance does not cover.

- How will I get my medication?

  NRT will be mailed to you.

- What credentials and/or experience do the counselors have? If they've never been addicted to cigarettes, how are they going to be able to help me?

  Our counselors have a range of educational background. All have completed a bachelor's degree. Most are enrolled in or have completed a masters-level counseling program. All counselors completed extensive training before the start of the study.

VIII. Counseling Call Schedule

**Table 1. Coaching Call Schedule**			
**Call Type**	**Window for Scheduling <u>Next Call</u>**	**Number of call attempts (Leave voice mail at call attempts 1, 3 and 6 only)**	**Time Blocked for Call (but may be**

			**shorter)**
Outreach (3 days after data collection is complete)	Call date and time set up by data collector	• Week of scheduled call - 6 call attempts<br>• Next week - 6 attempts	30-45 minutes
Pre-quit	QD after 14 days of Outreach call	• Week of scheduled call - 6 call attempts<br>• Next week - 6 attempts	20-30 minutes
Already-quit call	Up to 11-13 days after outreach call	• Week of scheduled call - 6 call attempts<br>• Next week - 6 attempts	30 minutes
QD + 1-3 day day	1 to 3 days post quit date	• Week of scheduled call - 6 call attempts<br>• Next week - 6 attempts	10-15 minutes
QD + 1-week	7-10 day post quit	• Week of scheduled call - 6 call attempts<br>• Next week - 6 attempts	10-15 minutes
QD + 2-week	14-17 days post quit	• Week of scheduled call - 6 call attempts<br>• Next week - 6 attempts	10-15 minutes
QD + 4-week	27-31 days post quit	• Week of scheduled call - 6 call attempts<br>• Next week - 6 attempts	10-15 minutes
QD + 8-week	55-59 days post quit	• Week of scheduled call - 6 call attempts<br>• Next week - 6 attempts	10-15 minutes
Optional calls – Use clinical judgement			
Motivation Call	As needed, record	• Collaborate to elicit participant motivation and commitment to make a quit attempt<br>• Resolve ambivalence toward change<br>• Goal is to set a quit date	10-15 minutes
Progress Call	As needed, record	• Collaborate to elicit participant relapse issues, discover new behaviors and problem solve.<br>• Revisit medication plan<br>• Goal is to set a quit date	10-15 minutes
Maintenance Call	As needed, record	• Quit maintenance check-in<br>• Medication check-in	10-15 minutes

Before making each call, counselors should review the participant's previous calls to familiarize yourself with their information, situation, struggles, etc. You can review information on the participant through their counseling file.

IX. Call Content (for counseling purposes, not data collection)

Table 2. Call Content

Objective	Outreach	Pre quit (QD)	QD +1-3	QD + 1-Week	QD + 2-Weeks	QD + 4-Weeks	QD + 8-Weeks
Participants may be randomized at 4 or 8 weeks to TLC or TLC+MTM. At that point they will be transferred to the assigned treatment protocols.							
**1. Call Opening**	x	x	x	x	x	x	X
**2. Program Overview and Lung Cancer Screening**	x						
**3. Motivation-Assess Readiness to Quit**	x	x	x	x	x	x	X
**4. Tobacco Dependence**	x						
**5. Quit History**	X						
**6. Comorbidities**	x						
**7. Physical Addiction**	x						
**8. Pharmacotherapy**	x	X					
**9. Smoking Cues and Triggers**	x	X					
**10. Coping Skills**	X	X					
**11. Support**	X						
**12. Goal setting and Quit Planning**	X	X	x	x	x	x	X
**13. Set Quit Date (Reduction)**	X	X	x	x	x	x	x
**14. Self-efficacy**	X	X	x	x	x	X	x
**15. Call Summary and Close**	X	X	x	x	x	X	x
**16. Tobacco Treatment Form**	X	X	x	x	x	X	X
**17. Quit Status - Follow-up**			x	x	x	X	x

**18. Medication and Withdrawal Follow-up**	x	x	x	X	x
**19. Relapse Prevention**	x	x	x	X	x
**20. Support – Follow-up**	x	x	x	X	

Table 2 is a guide to call content. It is intended that the counselor have flexibility to add or subtract modules as clinically appropriate.

Objective 1: Call Opening
Goals: Provide consistent and professional call opening. Ensure privacy and confidentiality.

1. Introduce self, title, and study at the beginning of every call.
- "This is Mary, your Coach with the University of Minnesota tobacco cessation study."

2. Explain purpose of the call and approximate timeframe
- I am calling for our scheduled 20-minute follow-up call"

3. Verify participant using two forms of identification (HIPAA)
- "Would you verify your name and date of birth for me please?"

4. State call is recorded for quality purposes
- "For your protection and privacy, our calls may be recorded for quality assurance and is confidential."

Objective 2: Program Overview and Lung Cancer Screening
Goals: Provide rationale for Program for Lung Cancer and Tobacco Treatment (PLUTO) call, build rapport and foster collaboration.

1. Briefly discussion rationale for call.
   a. Introduce yourself and explain purpose of this call and what types of questions you will be asking.
   b. Example: "Hello, I am aware that you enrolled in this study because you are interested in quitting smoking. In this first call, I would like to ask you some questions that will help us come up with a plan for medication, counseling and quitting smoking. We know that understanding certain factors related to your individual smoking pattern and history (how much you smoke, your challenges, understanding what worked in past quit attempts, etc) are related to successful quitting. Today we will talk about your history, any concerns you have and work together to come up with a plan tailored to fit your specific needs around smoking. At the end of our call, we will talk more specifically about setting a quit date and a schedule for future calls."

2. Inquire about lung cancer screening **<u>only as appropriate</u>**.
   a. Inquire about the reasons the participant decided to have a CT scan.
   b. Ask about when the scan is scheduled, or if it has already taken place.

c. Ask the participant if they are comfortable sharing the results.
d. Ask about whether there are plans for further follow-up and what they might be.
e. Ask about how they feel about the lung cancer screening results, for example “How do you feel about the information? Do you think this information may help you quit smoking? Does the information make it more difficult for you to quit smoking? Do you think you will get another CT scan next year?” (Questions to be adjusted to be clinically relevant to the screening result.)

3. Explain the purpose of counseling sessions: Counseling sessions will be provided because stopping smoking requires learning new behaviors and ways of thinking, a process that takes work over many weeks. Emphasize that counseling greatly increases success in stopping smoking by teaching skills for:

    a. Reducing and managing smoking urges and withdrawal symptoms until they go away entirely.

    b. “Unlearning” the habit of smoking by practicing alternative behaviors in situations that usually trigger smoking.

    c. Coping with negative moods, and stress without using tobacco to control emotions.

4. Explain the schedule for the first 8 weeks of counseling sessions:

    a. 7 counseling sessions over an 8-week period. The first is approximately 45 minutes and the remaining 6 will last approximately 15-20 minutes. Participants will receive their counseling calls as follows: pre-quit date, quit date, 3 days, one week, two weeks, one month, and two months after the quit date. There can be flexibility if needed.
    b. Sessions will teach skills for quitting smoking as well as remaining smoke free, by teaching skills for preventing relapse and recovering quickly from any lapses, should they occur.

5. Familiarize yourself with the participant’s smoking and work to build rapport with the participant before talking specifically about smoking habits and behaviors.

Objective 3: Motivation-Assess Readiness to Quit Smoking
Goals: Understand participant perspective, assess motivation to stop smoking and encourage change talk

1. The goal for this objective is to help participants move toward action (setting a quit date and having motivation to set the quit date).

2. Understand participant perspective

1. Ask participant how he/she feels about quitting smoking
2. Listen for and reinforce change talk: their desires, ability, reasons and needs to quit smoking. He/she may find it helpful to write down these reasons and keep them as a reminder to keep from smoking (or other strategy as appropriate/ of interest to the participant).
3. Acknowledge sustain talk: desires, ability, reason and need to smoke without reinforcing or confronting to build resistance.

3. Try to get the participant to talk about change, ask for elaboration when you hear change talk.! Hearing ourselves talk about the changes we want to make increases our motivation to take action to make the changes happen. Change talk is what leads to commitment to change and commitment language leads to actual behavior change. Use E.A.R.S. to respond to change talk.

- Elaboration “What did you do to quit in the past”
- Affirm “You are determined to make this quit stick!”
- Reflection “You really ‘need’ to quit smoking.”
- Summarize “Essentially, the lung cancer screen was what you needed to push to quit for good.”

Then, follow up with:

a. “What from your previous attempt might be helpful this time?

“You need to quit. Tell me more about that.”

4. Ask: “How important is it to you to quit smoking, using a 10-point scale where 0 equals ‘not at all important and 10 equals ‘extremely important?”
   a. Why are you at (chosen number) and not a lower number?”
   b. “What would move you to a higher number?”
   c. Summarize
   d. If participant is in the middle, use decisional balance. * See Decisional Worksheet

**Table 3. Decisional Worksheet Work sheet:<br>Examining the Pros and Cons of Behavior Change**
Ambivalence about the value of change focuses on the cost and benefits of both staying the same and making an attempt to change. Decisional balance asks four questions and is most useful when both

sides, pros and cons, are summarized.	
**Good things about smoking**	**Not so good things about smoking**
**Not so good things about Quitting**	**Good things about quitting**
Reason to stay the same	*Reasons for making a change*

Objective 4: Tobacco Dependence
Goal: Assess level of nicotine dependence

a. Explore and assess the participant's level of nicotine dependence. To understand current smoking status, ask these questions.
   a. Tell me about your tobacco use?
   b. What types of tobacco do you use?
   c. Do you currently smoke, every day, some days or not at all>?
   d. How many cigarettes a day do you smoke?
   e. At what age did you start smoking regularly?
   f. How soon after you wake up do you use your first cigarette?

Objective 5: Quit History
Goals: Understand previous quit attempts and medication use.

1. Explore the participant's history of quit attempts and previous use of nicotine replacement medications. To understand ask these questions.

   a. How many serious attempts have you made?
   b. What is the longest time you went without smoking cigarettes?
   c. What led you to start smoking again?
   d. Did you experience nicotine withdrawal?
   e. Have you tried any smoking cessation medications?

Objective 6: Comorbidities
Goals: Understand the connection between smoking and depression, anxiety, alcohol abuse or major mental illness. This information will allow the coach to support the participant planning for these triggers/emotions.

a. Coaches will ask the following:
a. “How does mood, feeling sad, blue or depressed influence your smoking?”
b. “How does anxiety influence your smoking?”
c. “How does alcohol use influence your smoking?”
d. “How does marijuana use influence your smoking?”
e. “How does mental illness influence your smoking?”

Objective 7: Physical Addiction
Goals: Describe physical dependence, nicotine tolerance and nicotine withdrawal to the meet the participants’ level of interest.

a. There are good reasons why quitting smoking can be so challenging despite your desire to quit. It is not about willpower. It is the addicting drug called nicotine, the substance that makes it so hard to stop smoking. Coaches could share the following:
a. “What do you know about how nicotine changes your brain?”
b. What do you know about nicotine tolerance?”
c. What do you know about how the body responds to no more nicotine (withdrawal)?”

Objective 8: Pharmacotherapy
Goals: Medications education, medication plan and instructions

1. Counselors should do the following:
    a. Let the participant know that medications can double the likelihood that they will be successful at quitting smoking.
    b. Let the participant know that you will be working with their primary care provider to arrange medication.
    c. Use your discretion to abbreviate or elaborate this intervention, depending on your judgment of the extent to which the participant is interested in smoking cessation medications.

2. Physical addiction and medication
    a. Explain that the physical addiction part of smoking can be successfully treated using medications that reduce withdrawal symptoms and urges to smoke.
    b. Explain that the medication will reduce the physical withdrawal from nicotine and allow them to focus on the other two aspects of nicotine addiction already discussed: the habit of pairing smoking with specific activities and the emotional addiction.
    c. Explain that reducing the physical symptoms will help them concentrate on ways to break the habit and learn new ways to cope with the emotional connections. D

d. Explain that nicotine replacement therapy reduces nicotine withdrawal symptoms by safely delivering nicotine without the harmful effects of cigarette smoke.

3. Invite the participant to express reservations or resistance to using medications and address these or any misconceptions by providing information and/or reassurance.

4. Ask participant if they have used any of these smoking cessation medications in the past. If so, identify medications that were (and were not) helpful and the participant's current preferences for smoking cessation medications. **The program provides nicotine patch, nicotine gum and nicotine lozenges.**

**Table 5. Medications**			
**Type/Purpose**	**Use**	**Side Effects**	**Comments**
**NRT/ for withdrawal**<br>**Gum**	Use as directed, park and chew, use ad lib	Mouth or stomach irritation	Do not eat or drink for 15 minutes before or during gum use.
**Lozenge**	Use as directed, park and chew, use ad lib	Mouth or stomach irritation	
**Patch**	Use as directed, avoid experimenting with patch	Skin irritation, vivid dreams, mild itching	Do not apply patch to areas of the skin that are inflamed, burned, or irritated.
Other<br>1. inhaler<br>2. nasal spray	Avoid acidic drinks	Mouth irritation, nasal passage irritation (spray)	
**Bupropion**	Start taking 7 days before quitting	Dry mouth, mild sleeping problems, shakiness, skin rash	Can help with withdrawal symptoms like depression and irritability<br><br>Bupropion can be used with NRT (patch, gum, lozenge)
**Varenicline**	Start taking 7 days before quitting<br><br>Take with food to reduce nausea	Nausea, gas, headache, vomiting Insomnia, changes in dreaming, constipation	Can help cut cravings and block pleasure from smoking<br>**MD should monitor for possible aggression and suicidality

5. Invite and answer questions about the purpose and proper use of smoking cessation medications.

   a. Describe how the medication works, proper use, contradictions, side effects and signs of too much or too little nicotine.

b. Discuss advantages of combination therapy, long acting and short acting NRT.

c. Refer participant to their provider if interested in other medications.

6. Responding to Participants Who Refuse Medications

    a. It is acceptable for participants who refuse smoking cessation medications to only receive counseling as the study treatment.

    b. If the participant relapses or has difficulty quitting, persist in attempting to persuade the participant to accept smoking cessation medications to assist in making another quit attempt.

7. Utilize the medication Appendix 2 for more information on pharmacotherapy.

Objective 9: Smoking Cues and Triggers
Goals: Identify people, places, things and feelings that are associated with smoking cigarettes

1. Explain that most people smoke cigarettes for three main reasons, and that all three reasons will be addressed during treatment. These reasons are:

    a. Smoking is a <u>habit</u> resulting from years of repetition and association with many activities of daily living.

    b. Smoking is an <u>emotional addiction</u>, a way people have learned to cope with negative moods (like depression, anger, and anxiety), and general stress of daily living.

    c. Smoking is a <u>physical addiction</u>, since it delivers the drug nicotine that causes cravings to smoke and withdrawal symptoms.

2. Explain the purpose of counseling sessions: Counseling sessions will be provided because stopping smoking requires learning new behaviors and ways of thinking, a process that takes work over many weeks. Emphasize that counseling greatly increases success in stopping smoking by teaching skills for:

    a. Helps to reduce and manage smoking urges and withdrawal symptoms.

    b. “Unlearning” the habit of smoking by practicing alternative behaviors in situations that usually trigger smoking

    c. Coping with negative moods, and stress without using tobacco.

3. Address Smoking Triggers

a. Have a participant consider about what goes with smoking cigarettes?

b. Most likely the participant Will increase their awareness of the people, places, things and emotions that go with smoking cigarettes

c. Smoking triggers are high risk situations or cues that bring on the urge to smoke. This happens because these situations and cues have been associated with smoking hundreds or thousands of times, over many years.

d. To prevent smoking relapses, it is necessary to address what these smoking triggers are for the individual participant. Once we have identified the triggers, we will be able to work with the participant to provide solutions on how to overcome them. Here are some sample questions:

   i. "During what situations do you anticipate being most tempted to have a cigarette?"

   ii. "What situations will be most difficult for you (can give examples if participant doesn't respond, e.g., being around others who smoke, watching TV, drinking coffee or alcohol)?"

Discuss relevant triggers

**People**	i.e. My spouse that smokes
**Places**	i.e. My friend's house where smoking is allowed inside
**Things**	i.e. Coffee, after meals
**Emotions**	i.e. Stress, boredom, anger, frustration

4. *Triggers Over Time – optional*
   a. A conversation, "I want to briefly talk about any concerns that you have for the future in regards to staying quit.?" If the participant does not come up with anything, you might say, "I know it is difficult to predict the future, but there are a couple known situations and triggers that have been difficult for other people who have quit, is it okay if I briefly talk to you about the three top triggers for relapse overtime that other people who have quit report?"

Objective 10: Coping Skills

Goal: Develop skills for coping with smoking cues and triggers

a. Skills for coping with smoking triggers

a. During your assessment of their previous quit attempts, the participant may have identified what helped them during the time they did quit. As a counselor, you will want to reinforce the coping skills that have been a success (kept the participant from smoking cigarettes) and work with participants to learn new coping skills to address the cravings in situations that have proved to be too difficult to resist.

b. By the end of this conversation, you want to help the participant understand their triggers and strategies that will help them cope successfully with their triggers.

My spouse that smokes	i.e. Ask spouse to smoke outside
My friend's house where smoking is allowed inside	i.e. Ask friend to visit at your house for a week or two
Coffee, after meals	i.e. Switch to tea, go for walks after meals
Stress, boredom, anger, frustration	i.e. Talk to a friend when feeling negative emotions, start a hobby to reduce boredom

b. Provide Psychoeducation

- It may be helpful for the participant to hear some educational points about stress when quitting. Ask the participant if you can share some points about stress and quitting
- If the participant states that weight gain is a concern, provide education about the reality of gaining weight and quitting smoking.

  i. Average weight gain 5-7 pounds.

  ii. People often confuse cravings for hunger

  iii. Nicotine affects blood sugar levels so quitting can cause cravings for sweets.

  iv. The harm of smoking far exceeds the hard of weight gain.

  v. Metabolism return to normal (becomes slower than when smoking).

  vi. Increased activity (exercise) level will help.

- It could also be really helpful to openly discuss slips and relapses with the participant to avoid any confusion they may have after quitting and for you to openly discuss how difficult and challenging quitting can be for anyone.

  vii. Slip: smoking after quitting, but getting back on track with quitting

  viii. Relapse: smoking after quitting, and continuing to smoke.

  ix. Slips and relapses are normal part of the quitting process

  x. You learn from your slip or relapse

a. Skils for Coping with Smoking Triggers
   i. Reinforce the coping skills that have been a success (kept the participant from smoking cigarettes) and work with participants to learn new coping

Revised: 1/19/2018

skills to address the cravings in situations that have proved to be too difficult to resist.

ii. During each call discuss triggers and strategies that will help them cope successfully with their triggers. Build their confidence by highlighting their successes. Affirm success!

iii. Sample questions in follow-up:
  i. "What is working for you?"
  ii. "What have you learned from these situations that could be useful as you move forward with quitting smoking?"
  iii. "What other coping skills are you considering?"

Objective: 11 Support

Goals: Enlisting support for smoking cessation

I. Discuss the following:

  a. Other smokers in the house, how will this interfere with quitting

  b. Discuss rules about smoking in the home and where the participant typically smokes

  c. Social situations that might interfere with participant quitting

  d. Sources of support in participant's life

  e. If needed, help participant brainstorm about possible sources of support.

  f. Many people trying to quit smoking do not want to tell anyone that they are quitting smoking. There are a variety of reasons for this. Help participants identify their reasons (fear of failing, fear of being "nagged," fear of what other smokers will say, etc.).

  g. Examples:

    - "Who are some people that will support you in this process?"
    - "What are some ways that others could be supportive?"

II. Sometimes people who are close to the person quitting do not know how to be helpful. People trying to quit are sometimes hesitant to tell them. This can be particularly true with partners. For those that have never smoked, it is sometimes difficult to understand why the smoker "doesn't just quit."

  a. Helping smokers identify and then articulate ways that others can be supportive, can help ease some of their fears. Some examples are:

    - "How might your spouse best support you in this process?"
    - "How could you help him to support you?"

III. Conversely, many smokers are afraid to tell their smoking friends. Smokers have a strong social connection. They often feel ostracized by nonsmoking society and rebellious about nonsmokers "judging" them. When one of their smoking friends quit, some smokers feel threatened. Those trying to quit understand this and are often hesitant to ask their smoking friends for support. They do not want to ask a smoker not to smoke around them. This is true even if the smoker is smoking in the quitting person's home or car! Yet, this support can be more important than support from nonsmoking friends, particularly in the case of a partner.

a. Help smokers identify ways that their smoking friends can support them. Help them find ways to articulate this to their smoking friends. Some examples are:

- "How could _____support you even though he does not want to quit?"
- "How might you talk to him about this?"

IV. People that have support through the process of quitting do better long term than those that do not have support. Helping smokers identify support people in their life can lead toward more success.

Objective 12: Goal setting and Quit Planning
Goals: Provide rational for setting smoking cessation- related goals and quit planning.

- There is a point in the coaching conversation where the participant has shared his/her smoking cues, environmental smoking triggers, ideas for coping with withdrawal/cues/triggers and ideas about support. Any of these steps can be small goals or steps toward quitting. Committing to a goal (step) can put the focus on action steps (vs not smoking) towards the ultimate goal of smoking cessation.

- Benefit of goals include:

1. A curious self-discovery process
1. Focus on what is working
2. Positives in the process
2. Encourages testing out ideas
3. Objective is to learn along the way
4. Wrap in participant values, strengths and preferences
5. Goals must fit and be relevant to the participant

- Participants begin thinking about when and how to change, less about whether and why. Goals setting is part of the planning process.
    - Participants generate solutions and coaches promote autonomy of decision making and avoid assigning goals.

- The focus is on participants' strengths, and personal assets tied to their change talk.

- Quit planning includes choice of quit date, quit medications, and goals.
  - "You have a quit date, and a medication plan. We talked about many steps you could take as you prepare to quit smoking (i.e. clean out your car, chew on straws or talk to your spouse about support). What is one thing you might try before our next call?"

- Goal follow-up
  - Coaches will follow up on participant progress and quit status.
    - Coach: "Last time we talked you were going to talk to your wife about support. Tell me how that went?"
    - Participant: "We decided one way to help me would be to go for a walk after dinner each night and focus on the fresh air going into my lungs."
  - Goal setting continues as participants may need to alter or change goals. Setting a new goal is a positive and confidence building part of the quit process.

Objective 13: Setting Quit Date and Smoking Reduction
Goals: Set a quit date and after a quit attempt an option to create reduction plan.

1. Ask participant to decide the calendar date of his/her Quit Date

2. Allow 7-10 days for NRT to arrive by United States Postal Service

3. Participant may consider choosing a Quit Date on a day when s/he:

   a. Anticipates minimal stress and can devote time to focusing on quitting

   b. Can celebrate the event by engaging in a special pleasurable activity with a supportive significant other

   c. Can arrange to be in a non-smoking environment and engage in activities that are not associated with smoking

4. Recommending a quit date is preferred, however, occasionally, "quitting spontaneously" is the participant's choice. Do not argue with the participant and ask them what their concerns are about setting a specific date. Usually this will result in participant discussion around ambivalence. Try explaining that it is best to put a quit date in the system, but the two of you can work together to allow participant to "quit spontaneously."

   a. Example:

   Co: Tell me what your concerns are about setting a quit date.

Cl: I just get nervous and then I can't quit. When I quit drinking 10 years ago I just said, 'That's it I'm quitting! And I did! I didn't think about it.
Co: Okay, so you have found in previous experiences that it works best for you to quit spontaneously. Understandably, everyone has their own preferences in how to quit. This time, let's pick a date to put in our system so that we can plan our call schedule together. Then we can work together to see how this is working for you when next we talk.

5. After a quit date has been attempted, participants may be offered the option of smoking reduction techniques.

- Psychoeducational points about scheduled tapering:
    - Choose a number of cigarettes to begin with.
    - Smoke them at regular intervals (e.g. every hour), not in response to a craving.
    - Gain control over smoking and reduce number at same time.
    - Generate tapering schedule from computer.
- Psychoeducational points about smoking reduction strategies:
    - Discuss these strategies with the participant and develop a plan and together decide which of the strategies to follow. Document the behavior plan in your notes.

Basic tips of smoking reduction method:

a. Purchasing and carrying only a certain number of cigarettes each day- base this number on the target level of reduction.

b. Increasing the amount of time between each cigarette

Hierarchical Reduction (Selective elimination of cigarettes)

- This involves eliminating cigarettes that are easiest for the individual to give up. For example, in the first week recommend giving up the easiest 25% of cigarettes. In the second week, they should give up the second easiest 25%.
- Eliminate cigarettes associated with particular locations or times of the day
- Eliminate particular cigarettes throughout the day

Scheduled Reduction (Increased Inter-cigarette Interval)

- This involves calculating the average time interval between cigarettes. Taking the average number of minutes the person is awake per day and dividing by the average number of cigarettes smoked per day, using data from the baseline week (smoking level prior to starting treatment). Then instructing the person to increase the interval by 25% the first week and 50% in the second week.

- Obtain a commitment/agreement from the person that they are to wait until the target interval has lapsed before having a cigarette. Of course, if they can wait more than the target interval, that is good. Also, a person should agree not to "make up" for missed cigarettes. For example, a person went on a plane ride and couldn't smoke, and when they get off the plane they smoke 2-3 cigarettes to "make up" for the extra skipped cigarettes.

- Example: Person smokes two packs (40 cigarettes) per day and is awake 16 hours (960 minutes) of the day. The average interval between cigarettes is 24 minutes. Instruct participant to wait on 30 minutes between smoking cigarettes in the first week. In the second week, to wait 36 minutes.

Through using tapering methods, always provide empathy. You may want to encourage their strength in working towards their goals of quitting or acknowledge how challenging this process is for them. *Example*: "I understand that certain situations are really challenging for you. I admire your strength to work towards your goal of quitting by ___________."

Other Strategies to Taper: *(use only if participant suggest these; however try to encourage scheduled tapering and smoking reduction methods as those are more effective methods.)*

1. Rationing: limiting the amount of cigarettes smoked per day

2. Delaying: sometimes holding a cigarette without lighting it, or trying to delay smoking until craving becomes too much, distracting oneself to delay smoking; consciously making an effort to delay gratification by smoking

3. Brand Switching: switching cigarette brands to a brand the participant does not like, or not as strong

4. Smoking half of each cigarette (limiting intake): not smoking full cigarette, only taking puffs at a time during cravings

5. Banning: banning smoking in certain places the participant usually smokes (in the house, in the car, banning smoke breaks, avoiding social smoking, bars, parties) to limit the places the participant can smoke.

6. Removing cigarettes from environment—keeping cigarettes in a usual place: Making cigarettes hard to find (having a partner or friend hide the cigarettes—or giving them to someone to avoid smoking).

Objective 14: Self-Efficacy
Goals: Determine confidence level; bolster self-efficacy.

1. Ask: "How confident are you in your ability to stop smoking, on a scale from 0-10, where 0 equals 'not at all confident' and 10 equals 'extremely confident'?"

   a. Why are you at (chosen number) and not a lower number?”
   b. “What would move you to a higher number?”
   c. Summarize
   d. If participant is in the middle, use decisional balance. * See decisional worksheet

2. Reframe past “failed” quit attempts as valuable learning experiences:
1. Successful ex-smokers usually made several quit attempts before stopping smoking for good.
2. Lessons learned from past quit attempts will be useful in identifying
   i. Stop smoking methods to try again now
   ii. Relapse situations to better anticipate and plan for

Objective 15: Call Summary and Close

Goals: Summarize major ideas covered in the call; a consistent call close, and set up next call

1. Summarize the highlights of the call:
   a. Participant strengths, genuine affirmations, not cheerleading
      i. You are determination and have good insight into what you need to do to be successful.”
   b. Warm call close to build self-efficacy
      i. “You put a lot of thought into our call today. I am confident that when you get to your quit date you will be successful.”
      ii. Personalize, use participant’s name.
      iii. Show appreciation for their time.
         1. “Mary, thank you for your time this evening.”
2. For each call, assess the need to mail out mediation. Especially, when following –up.
   a. Verify participant’s mailing address.
   b. Schedule next call – date and time.
   c. Share toll free number incase participant needs to reschedule.
3. Reinforce next steps to end call on action items and personalized call conclusion.

Objective 16: Complete Tobacco Treatment Form (see Appendix E.)
Goal: Fill out form for every call

1. Enter the next scheduled call into the Tobacco Treatment Form
2. The following information should be entered from this call:
    a. Type of Call
    b. Duration of call
    c. Smoked since last call (yes/no)
    a. If yes, cigarettes per day
    d. Medication type currently being used (Do NOT check if they haven't started using it yet)
    e. Call topics covered (checklist)
    f. Summarize progress and the quit plan.

Objective 17: Quit Status (Follow up)
Goal: Discuss progress and quit status

1. Focus on the participant's quit planning and quitting experiences.
    a. Ask open-ended questions about quit planning and quitting experiences.
        1. "How is it going?"
2. Reassess the participant's tobacco use and next steps.
    a. Have you smoked a cigarette, even a puff in the last 7 days?
    b. Where you able to reduce your cigarettes per day?
    c. On average, on the days you smoke, how many cigarettes a day do you smoke now?
    d. How ready are you to set a new quit date or start/resume smoking reduction?
    e. What is your new quit date?
3. Have you received your medications?
4. Continue collaborate approach, try not to provide advice.
    a. Affirm progress and learning

b. Build participant self-efficacy to continue the process of quitting smoking.

c. Normalize the ups and downs of quitting smoking.

5. As needed, re-assess readiness to quit smoking. See Objective 3.

Objective 18: Medication and Withdrawal (Follow-up)

1. How is the participant tolerating the medications?
   a. "How have you been feeling while taking the medication?"
   b. "Do you feel the medications you are using are effective?"
      i. Check for proper medication use
   c. "Are you experiencing any side effects?"
2. How much NRT is the participant using?
   a. "How much ad lib NRT are you using a day?"
   b. Does the participant need to increase ad lib NRT use?
3. Check in with the participant about their withdrawal symptoms
   a. "How have you been coping with withdrawal symptoms?"
   b. Discuss coping skills to better manage withdrawal symptoms
4. See Appendix B for more information on each medication

Objective 19: Relapse Prevention
Goal: Help participants prepare and plan on how to face a variety of temptations, stressors, and the pull of old habit patterns.

1. Staying on track can be challenging. Discussing smoking cues, triggers and high risk situations is an on-going process with smoking cessation.
   a. Smoking triggers are high-risk situations or cues that bring on the urge to smoke. This happens because these situations and cues have been associated with smoking hundreds or thousands of times, over many years.
   b. To prevent smoking relapses, it is necessary to address what these smoking triggers are for the individual participant. Once we have identified the triggers, we will be able to work with the participant to provide solutions on how to overcome them.

2. If the participant had a close call, or may be slipping address the abstinence violation effect. The abstinence violation effect (AVE) occurs when an individual made a personal commitment to

abstain from smoking and has lapsed at least once. The AVE occurs when the person attributes the cause of the initial lapse (first violation of abstinence) to internal and global factors within (e.g., lack of willpower or underlying addiction or disease). Teach the participant how to minimize the size of the relapse/slip by directing attention to the more controllable external/situational factors that triggered the lapse (e.g., high-risk situations, coping skills, and outcome expectancies), so that the person can quickly return to the goal of abstinence. For those who slipped and didn't go back to smoking at their pre-quit levels, congratulate them for their strength for continuing with not smoking instead of thinking, "I blew it" and going back to old pattern.

3. Reinforce the coping skills that have been a success (kept the participant from smoking cigarettes) and work with participants to learn new coping skills to address the cravings in situations that have proved to be too difficult to resist.

4. Everyone's quit process is unique

    a. "What triggers have you discovered since our last call?"

    b. If slipped, "What happened?" – be non-judgmental

    c. If slipped, "Have you smoked since?" – curious tone

    d. "The next time this (slip) situation comes up, how would you handle it?"

    e. Normalize slips and relapse, important message is to help participant get back on track.

Objective 20: Support (Follow-up)

1. Check in with participant about the support they have been receiving as a result of quitting. Discuss the following:

    a. How would you describe the support you are receiving while quitting smoking?"

    b. "Who do you need to communicate with about your quitting smoking?"

    c. "What is your plan to get the support you need?"

X. Missed Call Protocol

If a participant does not answer a scheduled phone call, the counselor will attempt to call the participant 6 times over the following week. If no answer or response, then the counselor will skip a week and try calling 6 more times on over the following week.

Voicemail Scripts:

1st Missed Call (1st week)

Hi [name of participant]. This is [counselor's name] from the Smoking and Lung Cancer Screening Study. I was trying to reach you for the call we had scheduled at this time. I will try you again, but if you could call me back at 1-888-xxx-xxxx to reschedule I am here until [time] today. I hope to talk to you soon.

3rd Attempt (1st and 2nd week)

Hi [name of participant]. This is [counselor's name] again. I am sorry I keep missing you. I look forward to talking to you regardless of where you are with quitting. Please call me back to reschedule at 1-888-xxx-xxxx. I look forward to hearing from you.

6th Attempt (1st week and 2nd week)

Hi [name of participant]. This is [counselor's name] again. I have tried to reach you a few times and can't seem to connect. I would like to talk to you whether you have quit smoking or not, but realize this might not be a good time for you. I will try to reach you again in a few weeks. However, please do not hesitate to call me anytime at 1-888-xxx-xxxx. Sorry I missed you this week. I hope all is well!

Appendix A1: Motivational Interviewing

A. Motivational Approach to Smoking Cessation Treatment

1. Motivational interviewing is a collaborative, goal oriented, method of communication with particular attention to the language of change. It is designed to strengthen an individuals' motivation for and movement toward a specific goal by eliciting and exploring the person's own arguments for change.
2. Key principles include: resist the righting reflex, understand the participants' motivation, listen to the participant and empower the participant.
3. The four processes of motivational interviewing include:
    a. Engaging, the process of establishing a mutually trusting and respectful helping relationship.
    b. Focusing, the process by which a coach and participant develop and maintain a specific direction in the conversation about change.
    c. Evoking, involved eliciting the participant's own motivation for change. Embracing the participant's own ideas and feelings about why and how to change.
    d. Planning is when a participant's motivation reaches a threshold of readiness and begins taking more about when and how to change and less about whether or why.

B. Spirit of Motivational Interviewing

1. The participant understands that his/her expertise and wisdom about stopping smoking mostly reside within the participant. Participant–centered approach.
- Participants are the experts on themselves. The coach is a companion who typically does less than half of the talking. Within the partnership is an attitude of acceptance of what the participant brings to the calls.
- Coach avoids the expert trap, communicating that, based on your professional expertise, you have all the answers to the participant's dilemma. Coach avoids trying to "fix" the participant.
- Coach promotes and encourages power sharing in the sessions in such a way that the participant's contributions substantially influence the nature of the session.
    a) Demonstrates curiosity about participant's ideas through open ended questions and reflective listening
    b) Identifies participant as the expert and decision maker
    c) Tempers advise giving and expertise depending on participant input (see E-P-E)
    d) Coach evokes participant's strengths and resources (affirming and validating) rather than probing for deficits

2. Coach demonstrates an active interest in, and effort to understand the participant's internal perspective (experience), to see the world through his/her eyes.
    A. Coach makes an effort to understand the participant's perspective and convey that understanding to the participant.
        a. Listening and reflecting are key skills for the coach.
    B. Coach effectively communicates understanding of the participant's point of view through accurate reflections.
    C. Acceptance of the participant is important. It is when people experience acceptance of themselves as they are that change becomes possible.
        a. To accept a participant is this sense does not mean you that you necessarily agree/approve of the participant's actions.
    D. Coach encourages participant to elaborate, beyond what I necessary to merely follow the story

a. “Tell me more”, “In what ways?”

E. Coach uses accurate complex reflections
   a. Simple reflections – stick close to exactly what the participant said.
   b. Complex reflections – adds meaning or emphasizes to what the participant has said. Making a guess about the unspoken content or what might be coming next.

3. In a smoking cessation program, participant engagement is key. This therapeutic alliance between the participant needs to be established in the very first telephone call. The participant-centered approach leads to participants being more actively engaged, more likely to stay in the program and adhere to and benefits of the smoking cessation program.

- Establish a trusting and mutually respectful working relationship with the participant.
- Be certain that the participant is in agreement with the treatment goals and plans.
- Collaborate with the throughout the smoking cessation program.

C. Motivational Interviewing Key Communication Skills

1. Asking open questions invite the participant to reflect and elaborate. Open questions help the coach understand the participant’s internal frame of reference and strengthens the collaborative relationship.
2. Affirming is to accentuate the positives. A coach will recognize and acknowledge that which is good, support and encourage.
3. Reflective listening forms a reasonable guess as to what the original meaning was, and gives voice to this guess in the form of a statement with the goal of continued exploration. Reflective listening focuses on the participant’s own narrative rather than a coach asserting his/her own understanding of it.
4. Summarizing. Summaries are essentially reflections that pull together several things that a participant has shared.

Appendix A2: Pharmacotherapy for Tobacco Cessation

A. Rationale for Using the Medication
Explain that the medication will reduce physical withdrawal from nicotine and allow participants to focus on the other two aspects of nicotine addiction already discussed: the habit of pairing smoking with specific activities and the emotional addiction. Explain that reducing the physical symptoms will help them concentrate on ways to break the habit and learn new ways to cope with the emotional connections.

B. Screening for Contraindications

- Participants with underlying cardiovascular disease, including recent myocardial infarction (heart attacks), life threatening arrhythmias, or severe angina
- Participants with active TMJ (gum only)
- Pregnant Participants
- Participants who are breast feeding
- Participants with an excessive amount of dental work (gum only)
- Participants with dermatological conditions such as psoriasis, eczema, atopic dermatitis (patch only)
- Participants under 18 years old

C. Dosing Guidelines
Although pharmacotherapy will be individualized, the purpose of this algorithm is to promote reasonable consistency in prescribing of smoking cessation medications across patients. The Guideline recommended five first line therapies, three of which will be included in first-line treatment and tobacco longitudinal care in the study: transdermal nicotine, nicotine gum and nicotine lozenge. Choice of a specific first-line pharmacotherapy should be guided by factors such as contraindications for selected patients, patient preference, previous patient experience with a specific pharmacotherapy (positive or negative), and patient characteristics. For monotherapy encourage patch for combination therapy encourage patch + gum or patch + lozenge.

MONOTHERAPY

1. Transdermal Nicotine

A. Who will receive transdermal nicotine?
   All study Participants may receive transdermal nicotine unless absolute contraindications exist or unless the Participant refuses the medication. The absolute contraindications are:
   1. Hypersensitivity to nicotine patch
   2. Recent (within 2 weeks) MI or severe arrhythmia
   3. Unstable angina
   4. Pregnancy

B. Initiating transdermal nicotine

1. Transdermal nicotine will be initiated on the Participant's planned quit date.
2. Transdermal nicotine will initially be prescribed at 21 mg/24 hours for those participants smoking ≥10 CPD. If participant smokes <10 CPD, initial dose will be 14 mg/24 hours for 4 weeks.
    a. If intolerable side effects supervene, the appropriate steps to take depend on the side effects.
        i. If the side effect is sleep disturbance, Participants should be instructed to remove the patch prior to going to sleep.
        ii. For mild skin irritation Participants will be encouraged to change the patch site and use 1% hydrocortisone cream.
    ii. For any other types of side effects, the dosage will be reduced to the next lowest dosage. If the dosage is 21 mg/24 hours and side effects supervene, the dosage will be reduced to 14 mg/24 hours and then to 7 mg /24 hours and then discontinued if side effects continue.

C. Continuation treatment with transdermal nicotine
1. All Participants who are successfully initiated and stabilized on transdermal nicotine will be maintained on their stable dose for a planned treatment exposure of 8 weeks unless intolerable side effects supervene.
2. Participants who start at 21 mg/24 hours for 4 weeks (and have not relapsed to smoking) will receive 14 mg/24 hours for 2 weeks and 7 mg/24 hours for 2 weeks (total 8 weeks). Those who received 14 mg/4 weeks initially (and have not relapsed to smoking) will receive 7 mg/24 hours for 4 weeks (total 8 weeks).

3. Nicotine Gum

A. Who will receive nicotine gum?

All study Participants may receive nicotine gum for monotherapy unless absolute contraindications exist or unless the Participant refuses the medication. The absolute contraindications are:

1. Recent (within 2 weeks) MI or severe arrhythmia
2. Unstable angina
3. Pregnancy

Some study Participants who refuse transdermal nicotine or who cannot tolerate transdermal nicotine because of dermatological side effects may use nicotine gum as their sole nicotine replacement, and it would be recommended as a scheduled rather than a prn medication.

B. Initiating nicotine gum for Participants who *are not* prescribed transdermal nicotine (monotherapy)

1. Nicotine gum will be initiated on the Participant's designated quit date.
2. As per the Guideline nicotine gum will initially be prescribed as 2 mg q 1-2h while awake for Participants who are less dependent (smoke <25 cigarettes per day/do not smoke within 30 minutes of waking); at 4 mg q 1-2 h while awake for Participants who are more dependent (smoke ≥25 cigarettes per day/within 30 minutes of waking).
3. The maximum dose of nicotine gum for Participants not receiving transdermal nicotine is generally 24 pieces/24 hours

b. Nicotine gum for Participants not on transdermal nicotine should be tapered over a 12 week period.

4. Nicotine Lozenge

A. Who will receive nicotine lozenge?

All study Participants may receive nicotine lozenge for monotherapy unless absolute contraindications exist or unless the Participant refuses the medication. The absolute contraindications are:

1. Recent (within 2 weeks) MI or severe arrhythmia
2. Unstable angina
3. Pregnancy

Some study Participants who refuse transdermal nicotine or who cannot tolerate transdermal nicotine because of dermatological side effects may use nicotine lozenge as their sole nicotine replacement, and it would be recommended as a scheduled rather than a prn medication.

B. Initiating nicotine lozenge for Participants who *are not* prescribed transdermal nicotine (monotherapy)

1. Nicotine lozenge will be initiated on the Participant's designated quit date.
2. Lozenge will initially be prescribed as 2 mg q 1-2h while awake for Participants who are less dependent (smoke <25 cigarettes per day/do not smoke within 30 minutes of waking); at 4 mg q 1-2 h while awake for Participants who are more dependent (smoke ≥25 cigarettes per day/within 30 minutes of waking).
3. The maximum dose of nicotine lozenge for Participants not receiving transdermal nicotine is generally 20/24 hours

b. Nicotine lozenge for Participants not on transdermal nicotine should be tapered over a 12 week period.

COMBINATION THERAPY

Combination therapy will consist of patch plus ad lib use of nicotine gum or lozenge.

A. Initiating nicotine gum for *Participants on transdermal nicotine*

1. Nicotine gum will be initiated on the Participant's designated quit date.
2. Nicotine gum will initially be prescribed as 2 mg gum q 1-2 h prn nicotine craving. Since most Participants will also be receiving transdermal nicotine, the maximum dose of nicotine gum will be 12 pieces (24 mg)/24 hours.
   a. If intolerable side effects supervene, Participants will be encouraged to reduce the dosage or discontinue nicotine gum.
   b. If Participants experience intense craving 4 mg gum prn may be used in this situation.

B. Initiating nicotine lozenge for *Participants on transdermal nicotine*

1. Nicotine lozenge will be initiated on the Participant's designated quit date.
2. Nicotine lozenge will initially be prescribed as 2 mg lozenge q 1-2 h prn nicotine craving. Since most Participants will also be receiving transdermal nicotine, the maximum dose of nicotine lozenge will be 12 pieces (24 mg)/24 hours.
   a. If intolerable side effects supervene, Participants will be encouraged to reduce the dosage or discontinue nicotine lozenge.
   b. If Participants experience intense craving 4 mg lozenge prn may be used in this situation.

Participants who smoke while receiving smoking cessation pharmacotherapies

A. During the initial quit attempt, Participants in this study who receive nicotine replacement are instructed not to smoke in part because concomitant nicotine replacement and smoking decrease the likelihood of successful quitting. Nevertheless, many patients who get nicotine replacement do smoke. In most cases this occurrence does not represent a serious risk.

B. Participants who are in EQC and receiving smoking reduction treatment may receive NRT while they are still smoking. See EQC Treatment plan.

D. Directions for use

Transdermal Nicotine Patch

- Choose an area of the skin on the upper part of the body or the upper outer part of the arm
- Make sure the skin is clean, dry, and hairless
- Hair will interfere with the application of the patch
- Do not shave; this may irritate the skin
- Do not apply patch to areas of the skin that are inflamed, burned, or irritated
- Apply patch to a different area each day
- The same area should not be used again for at least 1 week
- Remove patch from pouch
- Peel off half of the backing from the patch
- Apply adhesive side of patch to skin
- Peel remaining protective covering
- Press firmly with palm of hand for 10 seconds
- Make sure the patch sticks well to skin, especially around the edges
- Wash hands (nicotine on hands can get into eyes or nose and cause stinging or burning)
- Do not leave the patch on skin for more than the recommended 16-24 hours as this may cause skin irritation
- Adhesive remaining on skin can be removed with rubbing alcohol or acetone
- Dispose of used patch by folding onto itself and completely covering the adhesive area
- Water will not harm the patch if applied correctly (Participants may bathe, swim, shower, or exercise while wearing)

- Do not cut patch to adjust dose
- Keep all patches (new and used) out of reach of pets and children

Nicotine Gum

- Use gum according to dosing schedule to reduce cravings
- Chew each piece very slowly several times
- Stop chewing at first sign of peppery taste or slight tingling in mouth (~15 chews)
- "Park" gum between cheek and gum (to allow absorption of nicotine)
- Resume slow chewing when taste or tingle fades
- Repeat chew/park steps until most of the nicotine is gone (taste or tingle does not return, usually 30 minutes)

Gradually reduce dose of gum until use is discontinued. Strategies include:

- Chew gum for 10-15 minutes instead of 30 minutes
- Chew each piece for more than 30 minutes but reduce the number of pieces used daily
- Substitute ordinary chewing gum for nicotine gum
- To improve chances of quitting, use at least 9 pieces of gum daily.

Nicotine Lozenge

- Use lozenge according to recommended dosing schedule
- Place lozenge in mouth and allow it to dissolve slowly (nicotine release may cause a warm, tingling sensation)
- Do not chew or swallow the lozenge
- Occasionally rotate the lozenge to different areas of the mouth
- Lozenge will completely dissolve in about 20-30 minutes
- To improve chances of quitting, use at least 9 lozenges daily during the first 6 weeks

E. Potential Side Effects

Transdermal Patch

- Side effects in the first hour include mild itching, burning, or tingling

- After patch removal, the skin may appear red for 24 hours (If the skin stays red more than 4 days or swells, or if a rash appears, discontinue use and contact your health care provider)
- Vivid dreams or sleep disturbances
- Headache
- Local skin reactions

Nicotine Gum

- Chewing gum too rapidly can cause excessive release of nicotine, resulting in lightheadedness, nausea/vomiting, irritation of the throat and mouth, hiccups, and indigestion.
- Chewing gum will not provide same rapid satisfaction that smoking provides.
- Mouth soreness
- Hiccups
- Jaw muscle ache
- Dyspepsia
- Adhesion to dental work (Discontinue use if excessive sticking occurs)

Nicotine Lozenge

- The lozenge does not provide the same rapid satisfaction that smoking provides.
- Effectiveness decreases with some foods, including coffee, wine, juices, or soft drinks (Do not eat or drink for 15 minutes before or during lozenge use.)
- Nausea
- Hiccups
- Cough
- Heartburn
- Headache
- Flatulence
- Insomnia

Appendix A3: Customer Service

A. Rationale

Provide a consistent and professional call, ensure participant privacy and reassure call confidentiality. Specific elements for an appropriate coach call opening and call closing.

B. Skills for call opening and call closing

1. Call Opening

a. Identifies self by name/title/study
- Detailed description identifying program information

b. Greet caller in pleasant tone of voice
- Appropriate call energy
- Greeting is positive and inviting

c. Explains purpose of call and time frame
- Identifies the type of call and approximately how long it will take

d. Verify caller using two forms of identification (adheres to HIPPA guidelines)
- Uses member specific information to ensure accuracy (name, date of birth)
- Does not provide personal address, phone number, e-mail etc. to caller/member

e. States calls is recorded for quality purposes

2. Call Closing

a. Warm closure
- Offers an expression of optimism

b. Summarize what was discussed in the call

c. Thank you for your time
- Participant specifically thanked for the time that they spent in the call

3. Overall customer skills

a. Appropriate volume
- Not too loud or to soft
- Participant does not ask for answers to be repeated frequently

b. Sounds conversational
- Doesn’t sound like the conversation is being read from a script
- Use of proper inflection in voice

c. Professional dialogue
- Does not use jargon/acronyms or endearments (i.e. medical terminology that is unclear to the member, honey, sweetie etc.)

d. Doesn’t sound hurried
- Call isn’t being rushed to meet time agenda’s etc.

e. Effective time management of call
- Use of learned skills to guide and structure the call

f. Appropriate pace of call

- Gaps of silence are managed using bridging (informing a participant of what is occurring during quiet periods)
- No silence vs. too much silence

g. Does not interrupt caller
    - Participant is allowed to speak without disturbance
    - Coach does not talk over participant

h. Uses callers name periodically throughout the call
    - Participant name is used a minimum of two times
    - Preferably the participant's name is used at both the beginning and ending of the ending of the call
    - Avoid using the participant name too frequently
    - If uncertain as to the pronunciation of a participant's name ma'am or sir is appropriate

26.0 Appendix B. Tobacco Longitudinal Care

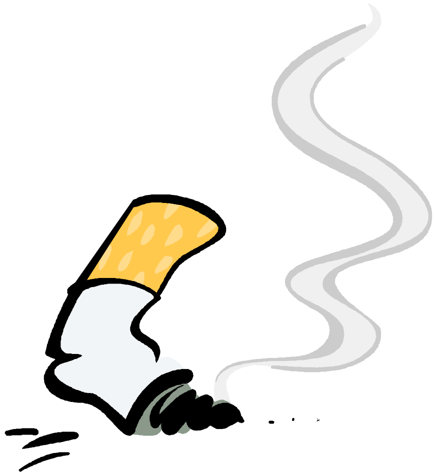

Tobacco Longitudinal Care

I. Guidelines for Using the Treatment Manual

Tobacco Longitudinal Care treatment components depend on the participant's particular smoking behavior at that call. There are four possible scenarios at the follow-up treatment calls. Each status has its own treatment plan. A brief definition of each status is provided below.

II. Possible Treatment Status

Complete Responder - Relapse Prevention

Relapse Prevention Treatment Plan is used when smokers have intentionally quit for at least 24 hours and successfully remained completely abstinent from smoking.

Incomplete Responder - Relapse, Ready to set Quit Date

The REPEAT QUIT ATTEMPT Treatment Plan is used when smokers have relapsed (or slipped) and wish to reattempt quitting smoking in the next 14 days. Seven consecutive days of smoking one cigarette or two consecutive week-ends of smoking constitute a relapse.

Incomplete Responder - Relapse, Ready to Reduce

The SMOKING REDUCTION Treatment Plan is used when smokers have relapsed and are not yet ready to make a repeat quit attempt (i.e., set a new quit date), but are interested in reducing the number of cigarettes smoked per day, or maintaining a reduction they have already achieved.

Incomplete Responder - Relapse, no plans to change

NO ACTION PLANNED treatment is used when smokers have relapsed and do not wish to reattempt to quit smoking or reduce their smoking at the time of their follow-up session.

III. Plan for Delivering TLC Behavioral Counseling Sessions

1. **Who should deliver TLC counseling interventions?** Behavioral counseling sessions should be delivered by a TLC staff member who has an ongoing, continuous relationship with the participant as their primary "point of contact." The counselor from first-line treatment is optimal.

2. **What modality should be used for delivering TLC sessions?** TLC treatment sessions are to be delivered on an individual basis. All treatment sessions will be delivered over the phone at the participant's convenience.

IV. Description of Tobacco Longitudinal Care and Medication Therapy Management Roles

Appendices covering intervention strategies that counselors should be familiar with as informed tobacco cessation interventionists, including smoking cessation medications. Counselors will coordinate the delivery of over-the-counter nicotine replacement therapy, including patches, gum, or lozenges or the combination of patch + gum or patch + lozenge. They will arrange medications as appropriate for each longitudinal care follow-up call. Counselors will discuss medication and "check in" for any concerns or questions about medication during each counseling call. If the participant has decided to use a prescription medication (such as bupropion or varenicline), the Medication Therapy Management pharmacist or participant's medical professional and the prescriber are primarily responsible for education, distribution, and monitoring of the prescription medication. Although counselors should be informed about these medications and discuss participant questions and concerns during each counseling call, the participant should understand that for prescriptions medication they should maintain contact with the Medication Therapy Management pharmacist or other health care professionals, such as their primary care provider.

V. Inform participant of randomized treatment group (at 4 vs. 8 weeks)

Some participants will be randomized to assessment of complete response to first-line treatment at 4 weeks, others to 8 weeks. At the time of assessment the participant will be identified (classified) as a complete or incomplete responded to first-line treatment. Complete responders are those who report no smoking at all, not even a puff, after a one week grace period following their quit date. All other participants are incomplete responders (any smoking at all after a one week grace period following the quit date).

Up until the 4 week phone call, treatment assignment has been unknown to the participant and the counselor. <u>If the person is a complete responder</u> they will receive at least monthly phone calls from the study team for one year, or at least quarterly phone calls for one year. The content of the calls will be the same, but the frequency differs in the two treatment groups. The purpose of these phone calls is to help him/her successfully quit smoking for good. S/he will have the ability to receive more frequent phone calls if it is determined that this would be helpful. Please also inform the person that if s/he wants to stop the phone calls or reduce the frequency of calls for any reason, they can.

<u>If the person is an incomplete responder</u> they will be randomized to TLC or TLC plus Medication Therapy Management.

VI. Assess smoking treatment status

Following the Tobacco Treatment Form, assess and assign the person to one smoking treatment status:

b. Relapse Prevention

c. Relapse, Ready to Set Quit Date

d. Relapse, Ready to Reduce

e. Relapse, No Plans to Change

First, determine whether s/he is currently smoking cigarettes. If s/he has quit smoking, assign the person to Treatment Status Relapse Prevention. If s/he is smoking, assess the person's interest in making another quit attempt. Feel free to encourage him/her to re-attempt. If the person would like to quit again in the next 14 days, assign the person to Treatment Status Relapse, Ready to Set Quit Date. You can treat the current call as a new "pre-quit" call or set another date. The goal is to get back onto the regular call schedule. The "1-3 day" and "4 week" calls are imperative and should occur on schedule, but the counselor has more flexibility regarding the timing or delivery of the 1 and 2 week calls.

For people who have relapsed and are not yet ready to make a repeat quit attempt, encourage Treatment Status Relapse, Ready to Reduce.

If the person is not ready to quit again within 14 days, determine whether they are smoking less. If so, encourage the person to maintain the reduced smoking level and advise the person to participate in smoking reduction treatment. If the person is back to their prior smoking level, encourage the person to reduce the amount they smoke and participate in smoking reduction treatment.

Inform the person we can help him/her reduce the amount of they smoke and that we can even provide them with medications to help them reduce the amount s/he smokes. State the following:

d. Smoking reduction is an option for people who aren't quite ready to completely quit.

e. Many smokers find after a quit attempt that they are able to smoke less than they smoked before they tried to quit.

f. Reducing the amount of cigarettes smoked may help the person quit smoking in the future.

Assign persons willing to participate in smoking reduction to Treatment Status Relapse, Ready to Reduce.

Assign persons who are smoking but not interested in quitting again or not willing to participate in smoking reduction to EQC Treatment Status Relapse, No Plans to Change.

VIII. Treatment Plans

Each smoking status category has a separate treatment plan.

A. Treatment Plan: Relapse Prevention

The goal of treatment for "abstinent" status is to reinforce the participant's successes and discuss strategies for maintaining the quit. As part of this, discussing relapse prevention and how to handle lapses is critical. We want to encourage self-awareness and problem-solving. We also want to assess the participants' use of NRT medication, as relevant. Remind participants that you will check in with them every month (or every 3 months if randomized to TLC-Quarterly).

1. Provide positive affirmation and reinforcement of the person's successes.

Sample Questions:

1. Own reactions: How does it feel to be smoke-free?
2. Others' reactions: How have others reacted?
3. Benefits of quitting: What have you noticed about the way you feel physically since quitting? What have you noticed about the way you feel about yourself?

Introduce idea that although reaching this point means that they have already "succeeded" at something very hard to do (actually quitting), it is still important to think about quitting as a process (and that by talking about possible future challenges, it will help to ensure their continued success):

Points to discuss/mention:

- May be ups and downs in terms of how "difficult" quitting feels.
- May be certain times or future situations where they will be tempted to smoke.
- For some people, doubts about really "doing it" or "pulling it off" may creep into their head.

2. Introduce notion of "planning".

Other former smokers have told us that it is helpful to plan and think about the future challenges before they are actually facing you. That way, you can make a plan for how to handle the ups and downs, just like you did when you planned your quit.

One of the ways we can learn about ourselves is to think back about other attempts we've made to change (like previous quit attempts or the current one) and look at what worked and what didn't. We

can use this knowledge to make a plan. That way, if temptations arise, you already know what you are going to do.

3. Identify person-specific challenges (or review from previous discussions).

Elicit danger situations (external events, internal states, activities) that might increase risk of smoking or relapse.

- Particular challenges person has already faced (e.g., stress, a night out with smoking friends, previous slip, negative moods – frustration, anger, depression) (past challenges are good indicators of future challenges).
- Anticipate future challenges over the next few weeks/months (e.g., celebratory events, weight gain, in situations with alcohol, pain, changes in life circumstances, being around certain people- social pressure, stress).
- Discovering warning signs (e.g., how will you know if you are in a situation that might increase your temptation to smoke?)

4. Discuss coping strategies. Brainstorm together and use previously discussed material.

Things to discuss/sample questions:

- How could you deal/ have you dealt with these situations?
- Elicit their personal behavioral strategies and reinforce strategies that have been successful.
  - Include other strategies as appropriate (e.g., relaxation, enlisting social support, distraction, substitution of alternative, positive behaviors – hobbies, exercise).
- Cognitive strategies.
  - What positive things could we tell ourselves?
  - Also important to notice the negative messages we might be giving ourselves. For example, in a tempting situation like feeling sad or being around other smokers….“after I smoke, I will feel better,” “I am missing out”). Important to challenge these thoughts and substitute a more helpful thought.

2. Lifestyle changes -What changes in lifestyle have you already made or could make to make it easier to stay smoke-free?

LAPSES

If the notion of lapses has not been discussed, it is important to mention that this may occur, despite our best planning, and how it is handled is really important. Remind the participant that rather than getting down on him/her self, the important thing is to learn from the experience and focus on getting back to being smoke-free quickly.

Points to discuss/mention:

- As you know, how we think about any slips is really important.
- Helpful to view as a temporary setback, related to external factors that we can work to better control in the future.
- Not helpful to view as personal flaw or weakness

Discuss ways person could talk self through a slip if it happened, how they would handle the situation with behavioral and cognitive strategies.

5. **Discuss use of NRT as appropriate.**

6. **Schedule follow-up call in one month.**

B. Treatment Plan: Relapse, Ready to Set Quit Date

If the person has smoked, but would like to continue to try to quit, congratulate them for their desire to quit again. Review what has been helpful during the time they were successful in quitting and what has contributed to their relapse.

Review the pre-quit call in the First-Line Treatment manual and use many of the same strategies (assess current consumption, what it was like for them when they were attempting to quit, reasons for quitting, and go over what triggered their relapse and their plans to avoid these triggers this time). Create a new plan with them based on their new knowledge. Encourage them to think of the previous attempt as an opportunity to learn what they need to successfully quit smoking. Create a new call schedule with the person.

Sample questions/comments:

- I know that you have successfully quit for __ weeks. What do you think helped you for the length of time you did quit? What kept you going?
- What was going on when you relapsed? What were you doing, thinking, feeling?
- What did you learn during this last quit attempt that can help you this time? (remind them that most people who successfully quit smoking have had previous attempts, indicating a process of learning)
- I am impressed with your continued determination to quit smoking. Where does your determination come from?

- Would you like to review your initial plan and make any changes based on what you learned this time?
- Should we set a new call schedule?

C. Treatment Plan: Relapse, Ready to Reduce

Overview
The goal of smoking reduction treatment is for the participant to reduce the amount of cigarettes smoked each day by at least 50% (one half) of the amount s/he was smoking prior to start of the initial quit attempt. Tell the participant the more s/he reduces the better.

- Working with participant, decide upon a target level of how many cigarettes per day the person will smoke and no more.

To help the participant reduce smoking, we will offer over the counter nicotine gum, lozenge or patch. Study physicians, Dr. Fu or Dr. Joseph, are available to provide guidance on choice and use of medications. Participants may be concerned about smoking and using nicotine replacement therapy at the same time. Instruct the participants they will use the nicotine gum or lozenge instead of smoking a cigarette. That way they will not put too much nicotine into their system.

Participants will also be instructed on several behavioral strategies and tips to reduce smoking.

Screening for NRT Use
Use the below form to screen for any potential contraindications to the use of nicotine replacement. If all the questions are answered NO, then it is safe to go ahead and offer the participant the use of nicotine replacement therapy. If the participant answers yes to any of the questions, consult with Dr. Joseph or Fu before providing any medications. Inform the participant that you need to talk with the study physician about whether nicotine replacement would be appropriate for that person.

**MEDICATION SCREENING FORM**		
**I am going to ask you a few questions to see if it is safe for you to use nicotine replacement therapy?**		
Have you been hospitalized for a heart-related condition in the past 2 weeks?	YES	NO
Have you had a recent myocardial infarction (heart attack) – *how recent?*	YES	NO
Are you currently pregnant or breastfeeding?	YES	NO/NA

NRT Treatment Guidelines

3. Basic information about nicotine replacement therapy can be found in the First-Line Therapy manual.

4. First offer choice of nicotine gum or lozenge (2 mg dose) to help participants reduce smoking. The recommended reduction plan is to replace each cigarette not smoked with a piece of gum or lozenge. Instruct the person to not use more than 20 pieces of the gum or lozenge in one day. If a person is a heavy smoker and you find the 2 mg dose isn't working consider a 4 mg dose.

5. At 2-4 weeks (at counselor's discretion), if 50% reduction has not been achieved, offer the participant the nicotine patch instead of nicotine gum or lozenge, but allow him/her to choose between continuing gum/lozenge or starting on the patch.

    - If participant smokes 20 or more cigarettes per day, offer the 21 mg strength patch.

    - If participant smokes less than 20 cigarettes per day offer the 14 mg patch.

6. Education about potential adverse reactions such as nausea, insomnia, palpitations and increase irritability.

Behavioral Strategies

Discussed below are several strategies that can be used to help the participant to reduce their smoking. Discuss these strategies with the participant and develop a plan and together decide which of the strategies to follow. Document in your note what the behavior plan.

<u>Basic tips</u>

- Purchasing and carrying only a certain number of cigarettes each day- base this number on the target level of reduction.
- Increasing the amount of time between each cigarette
- Use ad lib NRT to help achieve reduction goals

<u>Hierarchical Reduction (Selective elimination of cigarettes)</u>

- o This involves eliminating cigarettes that are easiest for the individual to give up. For example, in the first week recommend giving up the easiest 25% of cigarettes. In the second week, they should give up the second easiest 25%.
- o Eliminate cigarettes associated with particular locations or times of the day

- Eliminate particular cigarettes throughout the day

Scheduled Reduction (Increased Inter-cigarette Interval)

This involves calculating the average time interval between cigarettes. Taking the average number of minutes the person is awake per day and dividing by the average number of cigarettes smoked per day, using data from the baseline week (smoking level prior to starting treatment). Then instructing the person to increase the interval by 25% the first week and 50% in the second week.

- Obtain a commitment/agreement from the person that they are to wait until the target interval has lapsed before having a cigarette. Of course, if they can wait more than the target interval, that is good. Also, a person should agree not "make up" for missed cigarettes. For example, a person went on a plane ride and couldn't smoke, and when they get off the plane they smoke 2-3 cigarettes to "make up" for the extra skipped cigarettes.

- Example: Person smokes two packs (40 cigarettes) per day and is awake 16 hours (960 minutes) of the day. The average interval between cigarettes is 24 minutes. Instruct person to wait on 30 minutes between smoking cigarettes in the first week. In the second week, to wait 36 minutes.

- To help increase intervals between cigarettes could use ad lib nicotine gum or lozenge to replace the cigarette. Try to continue to gradually increase the intervals as much as possible.

Follow-up Phone calls and Schedule

At each f/u call, discuss the reduction plan and how it is working. Main goals of the f/u phone call are:

1. Achieve the goal of at least 50% smoking reduction

2. Encourage greater reduction, if possible

3. **QUIT Reminder -** at each f/u phone call, the participant should be asked/advised to make a repeat quit attempt. The quit reminder should be done at least monthly Remember the ultimate goal is to **quit smoking**. And when the participant is willing assign the person to Treatment Plan REPEAT QUIT ATTEMPT.

The phone call schedule is flexible and will depend upon the reduction plan developed with the participant. Weekly calls are recommended for the first month. After that, it is up to you and the participant. But the longest interval between calls should be no more than one month. In other words, the f/u phone call schedule should at least be monthly.

D. Treatment Plan: Relapse, No Plans to Change

If the person has smoked and has no desire to quit again, congratulate them for any successes they have had (if they did quit for a short time, they now know they can quit, etc.). Empathize with how difficult it is and discuss possible future attempts to quit. Explore the reasons for their decision. Encourage them to think of their experience as a learning opportunity, reminding them that quitting smoking is a process, and that they did quit for weeks. Remind them that you will check in with them every month (or every 3 months if randomized to TLC-Quarterly).

Sample questions/comments

f. What has helped you not smoke during the time you did quit?

g. If you decided to quit again in the future, what do you think you would do differently?

h. What are some of the pros and cons of your decision to continue smoking?

i. How can I be most helpful for you in the monthly counseling follow up calls?

VII. Interaction with Medication Therapy Management

Participants who are randomized to TLC + MTM will be referred to the pharmacist for an initial in-person visit. In that case, the pharmacist will be responsible for smoking cessation medication choices and prescriptions. They may continue to interact with the patient as clinically indicated until the 12 months of treatment (from initial quit date) is complete). The tobacco counselor is expected to continue to call the participant at least monthly, or more often as indicated above to complement MTM. They will be able to access pharmacist clinic records through the EMR to follow plans, and interact with the pharmacist at weekly meetings.

27.0 Appendix C. Medication Therapy Management: Pharmacist Assisted Medication Therapy Guide

I. Overview of Pharmacist-Assisted Medication Therapy Management
Participants who are incomplete responders to treatment, assessed at 4 vs. 8 weeks depending on treatment group, will be randomly assigned to TLC or TLC+MTM. For those assigned to TLC+MTM the counselor will complete a referral to the pharmacist, who will contact the participant to schedule the first in-person visit.

II. Collaborative Practice Agreements
Many states now allow the pharmacist to directly initiate, adjust and monitor medication therapy under written protocols and agreements with providers, called Collaborative Drug Therapy Management. Most require a detailed description of the collaborative role that the pharmacist shall play, including written protocols for specific drugs pursuant to which the pharmacist will base drug therapy decisions. Often the signature of physicians participating in the Collaborative Drug Therapy Management Guidelines must be kept on file. In addition, most states require a process for communication between the pharmacist and participating practitioners including requirements for documentation. At the UMN the clinical pharmacists who will see study participants have Collaborative Drug Therapy Management Guidelines with the Primary Care Center physicians. At the Minneapolis VA the research pharmacist will have a Collaborative Drug Therapy Management Guideline with Dr. Fu.
TOBACCO CESSATION: Sample COLLABORATIVE PRACTICE AGREEMENT
The Pharmacy Practice Act allows pharmacists to practice under a Collaborative Practice Agreement with individual physicians. Pharmacists may participate in the practice of managing and modifying drug therapy according to a written protocol between the specific pharmacist and the individual physician(s) who is/are responsible for the patient's care and authorized to prescribe drugs.
By signing this document, the named physicians agree that the named pharmacist may enter into a Collaborative Practice with them for the management of tobacco cessation in patients according to the attached protocol. By signing this document, the physician agrees with the tobacco cessation management outlined in the attached protocol.
TOBACCO CESSATION PROTOCOL AND COLLABORATIVE AGREEMENT APPROVED BY:

PHARMACIST CLINICIAN:

____
[INSERT PHARMACIST NAME] R.Ph., Pharm.D.

PHYSICIANS:

_______________________________	_______________________________
[INSERT PHYSICIAN NAME, M.D.]	[INSERT PHYSICIAN NAME, M.D.]
_______________________________	_______________________________
[INSERT PHYSICIAN NAME, M.D.]	[INSERT PHYSICIAN NAME, M.D.]

etc.

DATE OF IMPLEMENTATION: ___________
DATES ANNUAL REVIEW COMPLETED:

III. Visit Schedule and Content

A. **First in-person visit**

1. During the first in-person meeting the pharmacist will take a smoking history, with particular attention to the recent quit attempt and the participants reasons for incomplete response.
2. They will screen the participant for contraindications to medications. They will educate the participant about the menu of drug treatment options.
3. The participant and pharmacist will select medication(s) on the basis of contraindications, past experience, and patient preference.
4. The pharmacist and participant will review the participant's entire medication list to look for potential drug interactions and to be sure there are not other pressing medication issues, consistent with MTM principles.
5. The pharmacist will prescribe the medication(s) selected under the Collaborative Practice Agreement.
6. The pharmacist will use a template note in the electronic medical record to document information from the visit.
7. The electronic note will be sent electronically to the participant's primary care provider and the TLC tobacco counselor.
8. The pharmacist will arrange for a telephone follow-up visit 3 days, 1 week, and 4 weeks after the quit date.
9. The participant will receive contact information and an invitation to contact the pharmacist at any time with concerns, inadequate control of nicotine withdrawal, or medication side effects.
10. Complete Tobacco Treatment Form.

B. Follow-up telephone visits

1. Follow-up visits will usually be by telephone, but can be in-person if the participant prefers.
2. Review smoking since the last visit.
3. Review medication use/adherence.
4. Review potential side effects or adverse events from medications.
5. Review nicotine withdrawal status.
6. Review concerns/questions about other medications the participant might be taking.
7. The pharmacist will use a template note in the electronic medical record to document information from the visit.
8. Complete Tobacco Treatment Form.

C. Communication with Tobacco Longitudinal Care Counselor

1. The TLC tobacco treatment counselor will have access to MTM notes in the EMR.
2. The pharmacist will attend weekly care conferences and can interact with counselors about specific participants there.
3. The pharmacist can communicate with counselors regarding individual cases using the EMR mail function.

IV. FDA-Approved Medications for Smoking Cessation

The pharmacist will promote use of FDA-approved smoking cessation medications. Prescription drugs including bupropion, varenicline, nicotine nasal spray and nicotine inhaler will be available through MTM, in addition to nicotine patch, gum and lozenge.

ADVANTAGES	DISADVANTAGES	CONTRAINDICATIONS	USUAL DOSAGE
Non-nicotine tablet; easy to use; may be used with NRTs	May cause insomnia, dry mouth, headache, tremors, nausea, or anxiety	Pregnancy category B<br>Avoid in patients with seizure disorders, bulimia or anorexia nervosa, or history of head trauma and in patients currently using bupropion or an MAOI.	150 mg per day for three days, then twice per day for seven to 12 weeks. Start treatment one to two weeks before quit date.
Over-the-counter availability; flexible dosing; delivers nicotine faster than the patch.	No food or drink 15 minutes before use; frequent dosing<br>May cause jaw pain, mouth soreness, dyspepsia, or hiccups	Pregnancy category D; may use in pregnant women if nonpharmacologic measures fail and if the benefit outweighs the risk<br>Avoid in patients with dental problems or temporomandibular joint syndrome.<br>Cardiovascular precautions†	Patients who smoke < 15 cigarettes per day: one 2-mg piece of gum every one to two hours<br>Patients who smoke ≥15 cigarettes per day: one 4-mg piece of gum every one to two hours
Flexible dosing; mimics hand-to-mouth action of smoking; few side effects	Frequent dosing necessary<br>May cause mouth and throat irritation	Pregnancy category D<br>Cardiovascular precautions†	Six to 16 10-mg (only delivers 4 mg of nicotine) cartridges per day for three months; taper dosages over six to 12 weeks.
Over-the-counter availability; flexible dosing; delivers nicotine faster than the patch	May cause mouth soreness or dyspepsia<br>Frequent dosing necessary; no food or drink 15 minutes before use	Pregnancy category D<br>Cardiovascular precautions†	Weeks 1 to 6: one lozenge every one to two hours<br>Weeks 7 to 9: one lozenge every two to four hours<br>Weeks 10 to 12: One lozenge every four to eight hours<br>Patients who smoke their first cigarette of the day within 30 minutes of awakening should use the 4-mg dose; others should use the 2-mg dose.

ADVANTAGES	DISADVANTAGES	CONTRAINDICATIONS	USUAL DOSAGE
Over-the-counter availability; daily application; overnight use may reduce early morning cravings; few side effects	Less flexible dosing; slow delivery of nicotine<br>May cause skin irritation or sleep problems if worn at night	Pregnancy category D<br>Cardiovascular precautions†	Patients who smoke more than 10 cigarettes per day: 21 mg every 24 hours for six to eight weeks, step down to 14 mg every 24 hours for two to four weeks, then to 7 mg every 24 hours for two to four weeks<br>Patients who smoke 10 cigarettes or less per day: 15 mg every 16 hours for six weeks
Flexible dosing; fastest delivery of nicotine among NRTs; reduces cravings within a few minutes	Frequent dosing necessary<br>May cause nose and eye irritation or cough<br>Most addictive of the NRTs	Pregnancy category D<br>Cardiovascular precautions†	One or two 0.5-mg doses in each nostril every hour for three to six months; taper doses over four to six weeks

V. Assessment of potential contraindications to smoking cessation medications

A. General

1. Do you have heart disease (angina, heart attack)? Yes No
2. Do you have emphysema or chronic bronchitis? Yes No
3. Do you have asthma? Yes No
4. Have you had a stroke or a warning stroke (TIA)? Yes No
5. Do you have blockages in any of your arteries? Yes No
6. Have you had any vascular surgery? Yes No
7. Have you ever had any form of cancer? Yes No
8. Do you have high blood pressure? Yes No

B. Nicotine Replacement Therapy

1. Are you currently pregnant? Yes No
2. Are you currently breastfeeding? Yes No
3. Have you had chest pains, angina, or a heart attack in the past two weeks? Yes No
4. Have you had side effects from nicotine patch, gum, inhaler, or spray? Yes No
5. Do you smoke less than 10 cigarettes per day? Yes No
6. Do you weigh less than 100 pounds? Yes No

C. Buproprion

1. Have you ever had a seizure? Yes No
2. Are you currently taking bupropion (Wellbutrin, Zyban®)? Yes No
3. Have you ever had an eating disorder, anorexia, bulimia? Yes No
4. Have you ever taken a monoamine oxidase (MAO) inhibitor? Yes No
5. Are you chemically dependent on alcohol, prescription medication, marijuana, cocaine, heroin, or stimulants? Yes No
6. Have you ever had a major head injury or concussion? Yes No
7. Are you currently taking an antidepressant or anxiety/nerve medication? Yes No
8. Are you taking theophylline? Yes No
9. Have you had brain surgery or a brain tumor? Yes No
10. Do you have liver or kidney disease? Yes No
11. If you are female, are you pregnant, planning to get pregnant or nursing? Yes No

D. Varenicline

1. Do you have kidney disease (other than urinary tract infections)? Yes No
2. Are you on kidney dialysis? Yes No
3. Have you ever seen a psychiatrist regularly?
4. Do you have depression?
5. Have you ever been hospitalized for a psychiatric disorder?
6. Do you take any medications for depression, anxiety, or other mental health issues?

If participant is interested in varenicline:

1. Are you feeling hopeless about the present or future?
2. Have you ever had a suicide attempt?
3. Have you had thoughts about taking your life or harming others in the past 12 months? (if yes, ask question 4)
4. Do you have a plan to take your life?

VI. Treatment Directions

The clinical pharmacist is authorized to initiate any of the therapies listed per this protocol. The following information will be taken into consideration when selecting therapy. Patients will be educated on the variety of products listed below that are available for treatment of Tobacco cessation. The final decision will be made based on the patient's desires and the clinical judgment of the pharmacist. In some cases, a combination regimen may be used. Examples of combination therapies include: bupropion and nicotine patch, bupropion and nicotine gum, or nicotine patch and nicotine gum (PRN only). The second line agents, clonidine or nortriptyline, will be possible alternatives, and the clinical pharmacist will consult the referring physician if these are deemed to be the best options for a particular patient.

VI. Treatment Guidelines

The research protocol includes a potential randomization to consultation with a Pharm D, or not (for nonresponders to initial treatment). The experimental treatment condition is the consultation (not the medications per se). The Pharm D might (or might not) recommend bupropiion or varenciline, but

under the clinical practice agreements from their practice, and not the study. The PharmD practice is guided by an algorithm that includes screening for contraindications to medications but is not experimental in nature. All the prescription practices are evidence-based and consistent with FDA-approved recommendations. Therefore, we describe the possibility of medication prescription in a more general sense than would be appropriate for a drug treatment trial.

A. Varenicline

Dose: 0.5mg qDay x3 days, then 0.5mg BID x 4 days, then 1mg BID x 2-3 months. Some patients may require longer maintenance therapy for up to 6 months. Quit date is set for 1 week after starting therapy, but may be set for later in therapy based on agreed upon treatment plan. Some patients may begin Varenicline and then reduce smoking by 50% from baseline within first 4 weeks, by an additional 50% in the next 4 weeks, and continue to reduce with the goal of complete abstinence by week 12.

*Dose and dosing frequency must be reduced in renal impairment. For CrCl < 30mL/minute: initiate at 0.5mg qDay, with a maximum dose of 0.5mg BID. In end-stage renal disease (receiving hemodialysis): maximum daily dose of 0.5mg qDay.

Administration: Twice daily dosing should be taken approximately 12hours apart with a full glass of water after eating.

Contraindications/Precautions: Varenicline will be used cautiously in individuals with preexisting psychiatric diseases, seizure disorders, and those with cardiovascular risk factors.

Other Drug Interactions: Varenicline is a substrate of the OCT2 transporter. Concomitant administration of Varenicline with either inhibitors of the OCT2 transporter or other OCT2 substrates may impact serum concentrations of Varenicline. Monitor concomitant use of Varenicline with the following drugs and consider dose adjustments if clinically indicated: bupropion, histamine (H2) receptor antagonists, quinolones, trimethoprim. Patients should be cautioned about use of alcohol with Varenicline and counseled to minimize their alcohol consumption due to increased risk for adverse effects.

Side Effects: Gastrointestinal (nausea, vomiting), insomnia, abnormal dreams, headaches, potential worsening mood/irritability

B. **Bupropion**
Dose: 150 mg QD x 3 days, then BID for 2-3 months. Some patients may require longer maintenance therapy for up to 6 months. Quit date is set for within the first 1-2 weeks of therapy.
*A lower dose of 150 mg QD may be used for elderly patients and/or those who are concerned about insomnia effects.
*Dose should not exceed 300 mg QD due to a dose-dependent increased risk of seizure
*Dosing frequency must be reduced in hepatic impairment and renal impairment. There are no exact recommendations for this dosing reduction; clinical judgment will be used. In severe hepatic cirrhosis, the dose should be 150 mg QOD.

Administration: Twice daily dosing should be taken 12 hours apart, but if insomnia occurs, the doses may be taken 8 hours apart. Dose may be decreased to 150 mg QD if side effects occur. If patient has made no progress after 7 weeks, may consider discontinuing therapy.
Contraindications: Bupropion will not be used in patients with seizure disorders, a history of or a current diagnosis of anorexia or bulimia, or with concomitant MAOIs.
Other Drug Interactions: Bupropion inhibits cytochrome P450 2D6 isoenzyme, therefore it may be necessary to adjust doses of those drugs that are metabolized through this enzyme. These drugs include: amitriptyline, bisoprolol, carvedilol, clozapine, desipramine, dextromethorphan, flecainide, fluoxetine, haloperidol, imipramine, labetolol, metoprolol, nortriptyline, oxycodone, paroxetine, promethazine, propafenone, propranolol, risperidone, sertraline, tamoxifen, thioridazine, timolol, tramadol, trazodone, venlafaxine. In addition, codeine and hydrocodone may be ineffective if used with bupropion. If a patient is receiving any of these medications, the situation will be discussed with the referring physician so that any necessary adjustments can be made. Bupropion should be used with caution in conjunction with other drugs that may decrease the seizure threshold (i.e. SSRI's, tramadol, theophylline, antipsychotics, systemic steroids, use/cessation of alcohol use), however, they may still be used together in this protocol, if deemed appropriate by the clinical pharmacist/pharmacy resident.
Side Effects: insomnia, dry mouth.[1, 6]

C. Nicotine Patch

Dose:
Nicoderm CQ (24 hours) OTC 21 mg (4 wk), 14 mg (2 wk), 7 mg (2 wk)
Nicotrol (16 hr) OTC 15 mg (8 wk)
Habitrol (24 hr) Rx: 21 (4 wk), 14 mg (2 wk), 7 mg (2 wk)
ProStep (24 hr) OTC 22 mg (4 wk), 11 mg (4 wk)

**Different or longer regimens may be used, depending on patient specific issues and based on clinical judgement

**Patient's starting patch dose will be correlated as best as possible to their current cigarette intake; one cigarette = 1 mg nicotine. The max dose of patches proven to be safe and effective is 44 mg.

If current Tobacco is <15 CPD, may consider starting with 14 mg patches
If current Tobacco is >30 CPD, may consider starting with a higher dose of nicotine patches.[2, 3]

Administration: Apply patch to hairless area between neck and waist, rotating sites. If vivid dreams occur with the 24 hour patch, this effect may be less with the 16 hour patch.
Contraindications: Tobacco while on the patch; immediately post-MI (within 2 weeks), serious arrhythmias, or serious or worsening angina
Side Effects: skin irritation, vivid dreams, insomnia, GI complaints

D. Nicotine gum (OTC)

Dose: 30 2 mg pieces/day (max) or 20 4 mg pieces/day (max). Patients with high nicotine dependence (previous severe withdrawal symptoms, Tobacco >1 PPD, or Tobacco first cigarette within 30 minutes of waking) or those Tobacco >25 CPD should be advised to use 4 mg gum.
Administration: Chew until peppery taste or "tingling" is felt, then "park" the gum between gum and cheek until sensation is gone (usually 1-3 minutes). Rechew every few minutes and "park" again. Chew each piece for 30 minutes, with 1 piece every 1-2 hours. Do not eat or drink anything except

water 15 minutes before or during chewing. Taper dose slowly. Not to be used for more than 6 months. Also may be useful for prn use only, especially in combination with other agents.
<u>Contraindications</u>: immediately post-MI (within 2 weeks), serious arrhythmias, or serious or worsening angina
<u>Side effects</u>: mouth and jaw soreness, hiccups, stomach discomfort, indigestion

E. Nicotine nasal spray (Rx)
<u>Dose</u>: 1-2 sprays per hour (1 in each nostril), not to exceed 5 sprays per hour or 40 sprays in 24 hours. 2 sprays (1 in each nostril) = 1 mg nicotine
<u>Administration</u>: Tilt head slightly back, and do not inhale, sniff, or swallow while spray is being administered. A minimum of 8 doses (16 sprays) should be used each day. Has not been studied for use >6 months.
<u>Precautions</u>: chronic nasal problems (polyps, allergies, etc.) or severe reactive airway disease. There is a higher abuse potential than with other NRT because of the quick absorption.
<u>Contraindications</u>: immediately post-MI (within 2 weeks), serious arrhythmias, or serious or worsening angina
<u>Side effects</u>: nasal & throat irritation, runny nose, sneezing

F. Nicotine Inhaler (Rx)
<u>Dose</u>: ~80 puffs = 4 mg nicotine = one cartridge
<u>Administration</u>: 3-4 puffs/minute (continuous puffing) for 20-30 minutes; will use a minimum of 4 cartridges/day; recommended to use at least 6 cartridges/day. Eating and drinking should be avoided for the 15 minutes prior to and during use. Recommended duration of therapy is 6 months with gradual reduction over the last 12 weeks. Inhaler should be stored at temperatures >40°F, as delivery of nicotine will significantly decrease at lower temperatures.
<u>Contraindications</u>: immediately post-MI (within 2 weeks), serious arrhythmias, or serious or worsening angina
<u>Side effects</u>: cough, mouth/throat irritation[1,]

G. Nicotine lozenge (OTC)
<u>Dose</u>: 2 mg lozenge is for patients who have their first cigarette 30 or more minutes after waking; 4 mg lozenge is for patients who have their first cigarette within the first 30 minutes after waking.
1 lozenge q 1-2 hours for 6 weeks (at least 9 lozenges/day), then 1 lozenge q 2-4 hours for 3 weeks, then 1 lozenge q 4-8 hours for 2 weeks. Should not exceed 5 lozenges in 6 hours or 20 lozenges in 24 hours.
<u>Administration</u>: Place lozenge in mouth and allow to dissolve slowly; this will take about 20-30 minutes. Do not chew or swallow it. Shift the lozenge from side to side in your mouth. Do not eat or drink 15 minutes before using or while the lozenge is in your mouth.
<u>Contraindications</u>: immediately post-MI (within 2 weeks), serious arrhythmias, or serious or worsening angina
<u>Side effects</u>: heartburn, indigestion, insomnia, nausea, hiccups, coughing, headache, and flatulence

H. Second-line therapies (use not authorized by this protocol; must be approved by physician)

Clonidine

Dose: 0.15 –0.75 mg/day PO or 0.1 – 0.2 mg/day transdermal

Administration: can use patch or PO form for 3-10 weeks

Side Effects: orthostatic hypotension, drowsiness, dizziness, dry mouth, constipation

Nortriptyline

Dose: 75-100 mg/day

Administration: can be used for 12 weeks

Contraindications: risk of arrhythmias, recovery after MI, concomitant use of MAOI's, known hypersensitivity

Side Effects: dry mouth, drowsiness, constipation, orthostatic hypotension, confusion, arrythmias

VII. Sample Template Note

QUIT DATE: ________________________________ Support:

____________________________________ and North Clinic: Pharm.D. 612-320-3100

Coping Strategies:____________________________ Follow-up: □ Phone call □ Clinic visit

____ □

RTC:____________

____ RX: □ given to patient □ called into pharmacy by ______________

MEDICATION PLAN			
√	PRODUCT	DOSE	DIRECTIONS
□	Nicotine Patch	□ 21 mg □ 14 mg □ 7 mg<br>______ mg daily for _____ days / weeks<br>______ mg daily for _____ days / weeks<br>______ mg daily for _____ days / weeks<br>______ mg daily for _____ days / weeks	Start Date: ________________<br>Use daily: □ every morning<br>□ every night
□	Nicotine Gum	□ 2 mg □ 4 mg<br>Flavor: □ Original □ Mint □ Orange	Start Date: ________________<br>Use up to _____ pieces per day for _____ days or _____ weeks. Taper use starting at _____ weeks. Discontinue use at _____ weeks.
□	Nicotine Lozenge	□ 2 mg □ 4 mg	Start Date: _________________<br>Use up to _____ pieces per day for _____ days or _____ weeks. Taper use starting at _____ weeks. Discontinue use at _____ weeks.
□	Nicotine Nasal Spray	One dose = one spray to each nostril<br>(10ml bottle = 100 doses)	Start Date: _________________<br>Use a minimum of _____ doses per day to maximum of _____ per day for _____ days or _____ weeks. Taper use starting at _____ weeks. Discontinue use at _____ weeks.

□	Nicotine Inhaler	One box = 42 cartridges	Start Date: ___________________<br>Use up to _____ cartridges per day for _____ days or _____ weeks. Taper use starting at _____ weeks. Discontinue use at _____ weeks.
□	Buproprion (Zyban®)	150 mg Tablet	Start Date: ___________________<br>Take one tablet by mouth daily for 3 days. Then take one tablet twice a day (at least 8 hours apart) for _____ months.

VIII. Pharmacist Handouts

<u>Patient Information for nicotine patches</u>

How it works

• Nicotine patches replace the nicotine that used to be obtained from cigarettes, thus reducing withdrawal symptoms (cravings, mood changes, headaches, sleeplessness or drowsiness).

What dose should you use?

• Depending on the number of cigarettes you smoked, your weight, and presence of cardiovascular disease, you will be started on either.

ii. 21mg patches: for > 10 cigarettes per day
iii. 14mg patches: for < 10 cigarettes per day, weight < 100 lbs, or presence of cardiovascular disease

• A seven-day supply of patches will be dispensed at each weekly class.

Dosing Regimen

• If you start with the 21mg patch, you will use the 21mg patch for 4 (four) weeks, then 14mg patch for 2 (two) weeks, then 7mg patch for 2 (two) weeks.
• If started on the 14mg patch, you will use the 14mg patch for 4 (four) weeks, then 7mg patch for 2 (two) weeks.
• Wear patch 24 hours a day if you crave cigarettes when you wake up.
• Wear patch 16 hours a day (place patch in morning, remove at bedtime) if you do not have cravings for cigarettes when you wake up.
• Start the patches on your quit date. Do not smoke while using nicotine patches.

How to apply the patch

• Apply the patch at the start of each new day (rotate site used each day).
• Select a relatively hairless area between your neck and waist (upper arm is good for most people).

• Open the sealed pouch.
• Take out the patch. The sticky side is covered with clear plastic.
• Pull the clear plastic off, one side at a time. Do not touch the silver part.
• Press the patch firmly on your skin with the heel of your hand for at least 10 seconds.
• Wash your hands after applying or removing a patch.
• Dispose of used patches carefully to assure children and pets will not be exposed.

Possible side effects

• Skin reactions or irritation (usually mild and self limiting)
• Hives (discontinue patch)
• Gastro-intestinal symptoms: nausea, diarrhea, constipation dry mouth
• Cardiovascular: Flushing, high blood pressure, palpitations, sweating
• Miscellaneous: insomnia, abnormal dreams

Patient Information for bupropion tablets

How it works

• Exact method of action is not known.
• Helps reduce nicotine withdrawal symptoms (anxiety, irritability, restlessness etc).
• Bupropion is believed to affect pathways in the brain that may be involved with nicotine addiction and withdrawal.
• Will only work with intensive patient counseling and behavior modification.

Who can't take bupropion

• People who have or have had seizures.
• People who have or have had an eating disorder.
• If you are taking Wellbutrin, Wellbutrin SR/XL or any other medication which contains bupropion.
• If you are taking an MAO inhibitor (eg. isocarboxazid (Marplan), phenelzine (Nardil), tranylcypromine (Parnate), deprenyl (Selegiline).
• If you are pregnant or planning to become pregnant, or are nursing.
• People who have significant liver or kidney disease
• If you are taking certain prescription medications that may interact

What dose should you take?

• On days 1, 2 and 3 take bupropion once a day in the morning.
• Starting on day 4 take bupropion twice daily (doses 8 hours apart)
• Until your quit date, you can smoke, but at your quit date you should stop using tobacco containing products
• Avoid alcohol

How long do you take bupropion?

• Take bupropion for 7 to 12 weeks to optimize long-term success rates.

Possible side effects

• Dry mouth, constipation
• Difficulty sleeping
• Dizziness, increased sweating
• Seizures (rare)

Managing side effects

• Keep water on hand and sip frequently
• Suck on hard candy and chew gum to keep mouth moist
• Take 1st pill in early morning and second pill in late afternoon or early evening.
• Keep doses 8 hours apart
• Avoid caffeine-containing foods and drinks

Patient information for varenicline

How it works

1. Varenicline interacts with nicotine receptors in the brain
2. It blocks some effects of nicotine
3. It promotes some effects of nicotine

Who can't take varenicline?

1. People who have a history of depression, especially suicide
2. Other mental health problems may play a role in deciding if this medication is good for you

What dose do you take?

1. 0.5 mg orally once daily for 3 days
2. Then 0.5 mg orally twice a day for 4 days
3. Then 1 mg orally twice a day for 11 weeks

How long do you take varenicline?

1. A total of 12 weeks

Possible side effects

- Nausea
- Changes in thinking or mood
- Headache
- Sleep disturbance
- Varenicline may impair your thinking or reactions. You may also have mood or behavior changes when you quit smoking.

Managing side effects

1. **Stop using this medication and call your doctor at once if you have:** any mood or behavior changes, confusion, anxiety, panic attacks, hallucinations, extreme fear, or if you feel impulsive, agitated, aggressive, restless, hostile, depressed, hyperactive (mentally or physically), or have thoughts about suicide or hurting yourself. Varenicline may impair your thinking or reactions. You may also have mood or behavior changes when you quit smoking. Until you know how varenicline and the smoking cessation process is going to affect you, be careful if you drive or do anything that requires you to be cautious and alert.
