# Supplementary material for: Optimizing Longitudinal Tobacco Cessation Treatment in Lung Cancer Screening: A Sequential, Multiple Assignment, Randomized Trial
Source: JAMA Netw Open. 2023 Aug 24;6(8):e2329903. doi: 10.1001/jamanetworkopen.2023.29903 (PMC10450571; doi:10.1001/jamanetworkopen.2023.29903)
Supplement: Supplement 2. — eTable. Observed and Adjusted Abstinence Rates and Sensitivity Analyses for Early Treatment Responders [file jamanetwopen-e2329903-s002.pdf]

Supplemental Online Content

Fu SS, Rothman AJ, Vock DM, et al. Optimizing longitudinal tobacco cessation treatment in lung cancer screening: a sequential, multiple assignment, randomized trial. *JAMA Netw Open.* 2023;6(8):e2329903. doi:10.1001/jamanetworkopen.2023.29903

eTable. Observed and Adjusted Abstinence Rates and Sensitivity Analyses for Early Treatment Responders

This supplemental material has been provided by the authors to give readers additional information about their work.

© 2023 Fu SS et al. *JAMA Network Open.*

eTable. Observed and Adjusted Abstinence Rates and Sensitivity Analyses for Early Treatment Responders

	TLC (n = 113)[d]	TLC-Quarterly (n = 113)[d]	Adjusted odds ratio (n = 113)[d]	TLC-quarterly compared with TLC (95% CI) (n = 125)[e]	
**Early treatment responders**					
Week 78: 6M prolonged abstinence	34/58 (58.6)	24/55 (43.6)	0.54 (0.25-1.17)	0.53 (0.26-1.11)	
Week 78: 7-day point-prevalent abstinence	40/58 (69.0)	32/55 (58.2)	0.64 (0.29-1.40)	0.69 (0.32-1.47)	
Week 52 (end of treatment): 7-day point-prevalent abstinence	42/60 (70.0)	30/54 (55.6)	0.53 (0.24-1.17)	0.63 (0.29-1.35)	

© 2023 Fu SS et al. *JAMA Network Open.*
