# Supplementary material for: Optimizing Longitudinal Tobacco Cessation Treatment in Lung Cancer Screening: A Sequential, Multiple Assignment, Randomized Trial
Source: JAMA Netw Open. 2023 Aug 24;6(8):e2329903. doi: 10.1001/jamanetworkopen.2023.29903 (PMC10450571; doi:10.1001/jamanetworkopen.2023.29903)
Supplement: Supplement 3. — Data Sharing Statement [file jamanetwopen-e2329903-s003.pdf]

Data Sharing Statement

Fu. Optimizing Longitudinal Tobacco Cessation Treatment in Lung Cancer Screening. *JAMA Netw Open*. Published August 24, 2023. doi:10.1001/jamanetworkopen.2023.29903

Data
Data available: Yes
Data types: Deidentified participant data
How to access data: Deidentified data underlying this article will be shared on reasonable request to the corresponding author (steven.fu@va.gov). A data sharing agreement may be required.
When available: With publication

Supporting Documents
Document types: None

Additional Information
Who can access the data: Additional information regarding the trial can be requested from the corresponding author (steven.fu@va.gov)
Types of analyses: De-identified data will be made available for research purposes.
Mechanisms of data availability: Without investigator support, with a signed data use agreement, and documentation of IRB approval or IRB-approved exemption.
